# Prevalence and incidence of fibromyalgia and associated severity in the United States using MarketScan

**DOI:** 10.1097/PR9.0000000000001451

**Published:** 2026-06-01

**Authors:** Moninder Kaur, Janek Rudnik, Paul Meisner, Andrew Williams, Bernard Lauwerys

**Affiliations:** a UCB Pharma Ltd, Slough, United Kingdom; b Department of Non-communicable Disease Epidemiology, London School of Hygiene and Tropical Medicine, London, United Kingdom; c UCB Biosciences Inc, Morrisville, NC, USA; d UCB Biopharma SRL, Brussels, Belgium

**Keywords:** Fibromyalgia, Epidemiology, Prevalence, Incidence, MarketScan, Severity

## Abstract

**Objective:**

Fibromyalgia is characterized by a range of symptoms, the most prominent being chronic widespread pain, fatigue, and unrefreshing sleep. This study estimated the diagnosed annual prevalence, incidence, and severity of fibromyalgia among adults (≥18 years) and the pediatric population (<18 years).

**Methods:**

This study used United States (US) claims data obtained from the Merative™ MarketScan^®^ Commercial (Commercial), Medicare (Medicare), and Multi-State Medicaid (Medicaid) databases.

**Results:**

In 2020, the annual prevalence of diagnosed fibromyalgia in adults and the pediatric population (0 to <18 years) was 0.44% (95% confidence interval [CI]: 0.44–0.45) and 0.01% (95% CI: 0.00–0.01), respectively, in Commercial + Medicare and 0.90% (95% CI: 0.89–0.91) and 0.003% (95% CI: 0.002–0.003) in Medicaid. The annual incidence of diagnosed fibromyalgia in adults and the pediatric population (0 to <18 years) in 2020 was 0.12% (95% CI: 0.12–0.12) and 0.003% (95% CI: 0.003–0.004), respectively, in Commercial + Medicare and 0.25% (95% CI: 0.25–0.26) and 0.002% (95% CI: 0.001–0.002) in Medicaid. Among adults with incident diagnosed fibromyalgia and ≥12, ≥24, or ≥36 months of follow-up, the proportion with severe fibromyalgia was 30.8%, 29.2%, and 30.1%, respectively, in Commercial + Medicare, and 37.8%, 39.4%, and 41.7%, respectively, in Medicaid.

**Conclusion:**

This study provides an updated overview of the diagnosed annual prevalence, incidence, and severity of fibromyalgia in the US and represents the largest study of this kind using the MarketScan database. Further research is needed to validate algorithms for identifying severe fibromyalgia using real-world data.

## Abstract 2

Supplemental Digital Content is Available in the Text.

Annual prevalence and incidence, and severity of diagnosed fibromyalgia in US pediatric and adult populations, reported using Commercial, Medicare, and Medicaid claims databases.

## 1. Introduction

Fibromyalgia is a syndrome characterized by a variety of symptoms that significantly affect quality of life, most prominently chronic widespread pain, fatigue, and unrefreshing sleep.^27,49^ Reported impacts include lower sexual intercourse frequency in women,^22^ work disability (34.8% of affected individuals),^50^ and suicide risk rate of 0.31 per 1000 person-years.^1^ In addition, fibromyalgia is associated with reduced physical activity,^42^ which is linked to increased cardiovascular mortality.^4,31^

Estimates of fibromyalgia prevalence in the general population range from 0.2% in Venezuela^18^ to 6.4% in the United States,^43^ with higher prevalence in middle-aged women.^29^ Incidence of physician-diagnosed fibromyalgia in the general population ranges from 0.33 per 1000 persons in the United Kingdom^11^ to 5.99 per 1000 person-years in South Korea.^25^ Among individuals with fibromyalgia, the reported proportion with severe fibromyalgia ranges from 38.8% in Italy, based on the revised Fibromyalgia Impact Questionnaire (FIQR),^35^ and 66.0% in the United States, based on the Fibromyalgia Impact Questionnaire.^23^ With increasing severity of fibromyalgia, pain increases and mood and sleep quality deteriorate.^39^

Several studies report the survey-based prevalence of fibromyalgia,^16,21,40,44,48^ but studies reporting the diagnosed prevalence are limited.^36,43^ This study presents the results from the largest observational descriptive study of diagnosed annual prevalence, incidence, and severity of fibromyalgia using US claims databases (Commercial, Medicare, and Medicaid).

## 2. Methods

We conducted an observational cohort study from January 01, 2016, to December 31, 2021, using de-identified administrative Merative™ MarketScan^®^ Research Databases (“MarketScan”) claims data. The study period was based on the introduction of the International Classification of Diseases 10th Revision (ICD-10) code for fibromyalgia in September 2015. MarketScan captures longitudinal, individual-level, administrative US claims data. This study included data from 3 MarketScan components: the Merative™ MarketScan^®^ (Commercial), the Merative™ MarketScan^®^ Medicare Database (Medicare), and the Merative™ MarketScan^®^ Multi-State Medicaid Database (Medicaid). Commercial consists of medical and drug data from several large US private employers, health plans, and government and public organizations, covering employees and dependents; therefore, employees from smaller companies or organizations may be underrepresented.

Medicare contains data on healthcare encounters and prescription claims of retirees with employer-sponsored supplemental insurance and includes only records in which both the Medicare-paid and employer-paid portions are linked.

Medicaid provides claims for low-income people, families and children, pregnant women, the elderly, and people with disabilities. It also contains data on aid category (eg, blind, disabled, Medicare-eligible) and race. As the Medicaid data structure differs from that of Commercial and Medicare, separate analyses for Commercial + Medicare and Medicaid were conducted.

### 2.1. Fibromyalgia diagnostic code

ICD-10 code M79.7 was used to define fibromyalgia.

### 2.2. Annual diagnosed fibromyalgia prevalence (2016–2020)

Annual diagnosed fibromyalgia prevalence in Commercial + Medicare and Medicaid was calculated by calendar year (2016–2020) and stratified by age (all ages, 0 to <2, 2 to <6, 6 to <12, 0 to <12, 12 to <18, 0 to <18, ≥18, 18 to <40, 40 to <50, 50 to <60, 60 to <70, 70 to <80 and ≥80), sex, region (Northeast, North Central, South, West, and unknown), and race/ethnicity (White, Black, Hispanic, Other, and Missing), where possible. Regional data were only available in Commercial + Medicare, and data on race/ethnicity were only available in Medicaid. Annual prevalence was defined as the proportion of individuals with ≥2 diagnostic codes of fibromyalgia in the enrolled general population with continuous enrollment in the specific calendar year. A single diagnosis code could be incorrectly coded or used as a rule-out criterion, rather than an actual condition; thus ≥2 diagnosis codes were required to confirm fibromyalgia and reduce false positives.

First diagnosis was the first observed date of fibromyalgia diagnostic code within the specific calendar year, from January 01, 2016, to December 31, 2020 (fibromyalgia identification period). The second diagnosis was within 1 year of the index date but separated by ≥1 day from the first diagnosis. The second diagnosis period ran from January 02, 2016, to December 31, 2021 (fibromyalgia confirmation period).

As some individuals with fibromyalgia may not seek repeated clinical care within the same year, annual prevalence estimates based on ≥1 diagnosis code were also reported.

### 2.3. Annual diagnosed fibromyalgia incidence (2017–2020)

Annual diagnosed fibromyalgia incidence in Commercial + Medicare and Medicaid was calculated by calendar year (2017–2020), stratified by age, sex, region, and race/ethnicity. Incidence was defined as the proportion of individuals newly diagnosed with fibromyalgia (those with a first-ever recorded diagnostic code for fibromyalgia) in that specific year, out of the general population enrolled on January 1 of the specific calendar year who: (1) did not have a fibromyalgia diagnostic code recorded during the 12 months preceding the index date; (2) had ≥12 months of continuous enrollment. As for fibromyalgia prevalence, 2 diagnosis codes were used to confirm the presence of fibromyalgia and calculate incidence.

The index date for incident cases of fibromyalgia was the first observed date of diagnosis within the index calendar year, from January 01, 2017, to December 31, 2020 (fibromyalgia identification period). The second diagnosis had to occur within 1 year of the index date, separated by ≥1 day, and could occur between January 02, 2017, and December 31, 2021 (fibromyalgia confirmation period).

Similar to annual prevalence, annual incidence estimates based on ≥1 diagnosis code were also reported.

### 2.4. Severity inferred from consultations and medications

Severity of fibromyalgia was estimated among incident individuals with fibromyalgia (2017–2020) with ≥12, ≥24, and ≥36 months of follow-up in adults (≥18 years) and adolescents (12 to <18 years). It was not possible to replicate validated self-reported severity definitions, eg, American College of Rheumatology (ACR) 2016 criteria, using electronic health record data.

Individuals were inferred as having severe fibromyalgia if they met any of the following criteria from the index date (see Table 1, supplemental digital content, http://links.lww.com/PR9/A410): >5, >10, or >15 diagnostic codes/claims within 12, 24, or 36 months of first diagnosis, respectively, incident events of disability, incident hospitalization at pain clinics, incident visits to pain clinics, ≥5 concomitant predefined medications potentially prescribed for fibromyalgia along with pregabalin, duloxetine, and gabapentin.

### 2.5. Comorbidities

Comorbidities and Charlson Comorbidity Index (CCI) categories were described among incident individuals with fibromyalgia (2017–2020) at baseline (12 months before index date) and with ≥12, ≥24, and ≥36 months of follow-up in adults (≥18 years). Charlson Comorbidity Index measure was defined based on Deyo et al. (1992)'s adaptation of the CCI for use with ICD-9 administrative databases.^13^ ICD-9 codes were identified from the publication by Deyo et al., and scores were calculated using the original CCI by Charlson et al. (1987).^7,13^ ICD-10 codes were obtained from Quan et al. (2011).^33^

### 2.6. Statistical analysis

Data were analyzed using descriptive statistics. Categorical variables were summarized using frequencies and proportions. All analyses were conducted using SAS 9.4 (SAS Institute, Inc). Prevalence and incidence estimates were generally reported to 2 decimal places, with additional decimal places included for values close to zero, as appropriate.

## 3. Results

### 3.1. Annual prevalence of diagnosed fibromyalgia with ≥2 diagnostic codes (2016–2020)

#### 3.1.1. Commercial and Medicare

**Figure 1.**
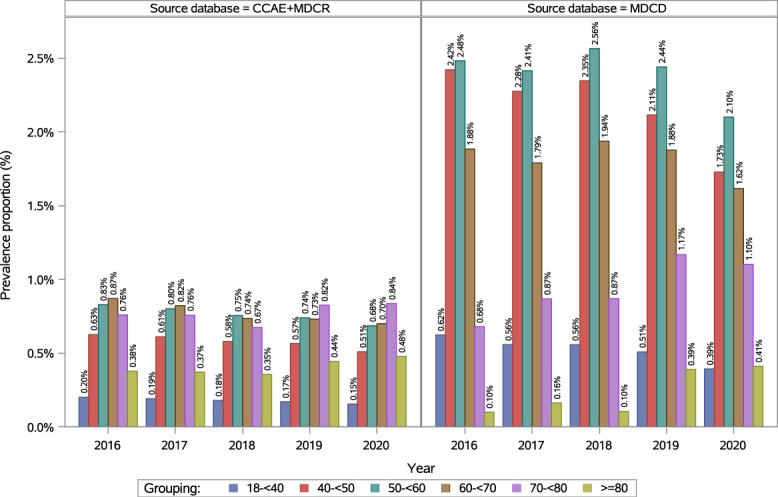
Annual prevalence of diagnosed fibromyalgia in the adult population in Commercial + Medicare and Medicaid databases. CCAE, Commercial Claims and Encounters database; MDCR, Medicare Supplemental and Coordination of Benefits Database; MDCD, Medicaid Database.

In adults (≥18 years), the annual prevalence of fibromyalgia decreased steadily each year from 0.55% (95% CI: 0.54–0.55) in 2016 to 0.44% (95% CI: 0.44–0.45) in 2020 (see Table 2, supplemental digital content, http://links.lww.com/PR9/A410). Prevalence increased with age, peaking in the 70 to 79 years age group in 2020 (Fig. 1; see Table 2, supplemental digital content, http://links.lww.com/PR9/A410). In 2020, prevalence was 0.80% (95% CI: 0.79–0.81) in female adults and 0.06% (95% CI: 0.06–0.06) in male adults, resulting in a female-to-male ratio of 13.61 in 2020 (see Figure 1, supplemental digital content, http://links.lww.com/PR9/A410; see Table 3, supplemental digital content, http://links.lww.com/PR9/A410). Among regions, the highest prevalence was observed in the North Central region, ie, 0.62% (95% CI: 0.61–0.63) in 2016 and 0.55% (95% CI: 0.54–0.56) in 2020 (see Figure 3, supplemental digital content, http://links.lww.com/PR9/A410; see Table 4, supplemental digital content, http://links.lww.com/PR9/A410).

**Figure 2.**
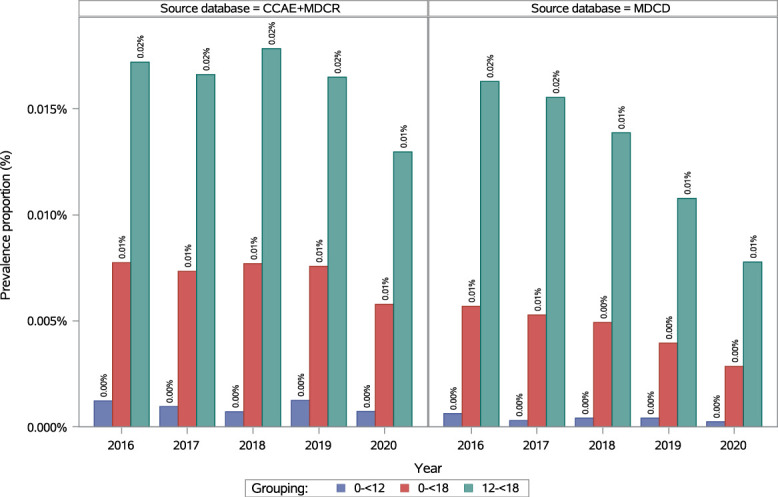
Annual prevalence of diagnosed fibromyalgia in the pediatric population in Commercial + Medicare and Medicaid databases. CCAE, Commercial Claims and Encounters database; MDCR, Medicare Supplemental and Coordination of Benefits Database; MDCD, Medicaid Database.

**Figure 3.**
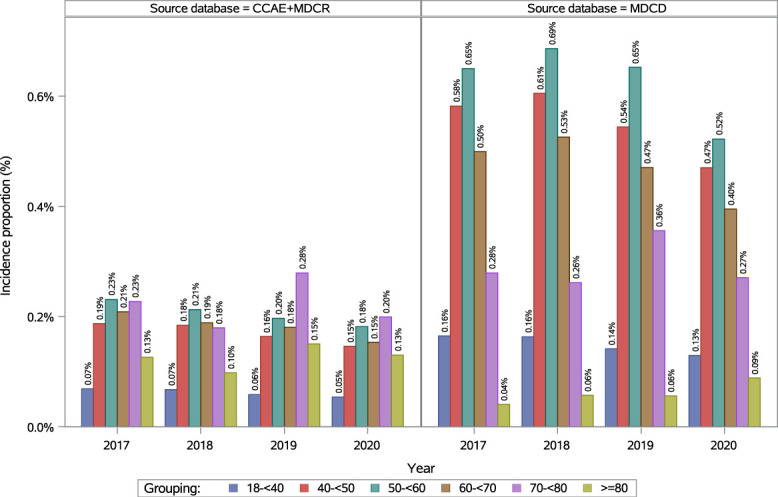
Annual incidence of diagnosed fibromyalgia in the adult population in Commercial + Medicare and Medicaid databases. CCAE, Commercial Claims and Encounters database; MDCR, Medicare Supplemental and Coordination of Benefits Database; MDCD, Medicaid Database.

In the pediatric population (0 to <18 years), the annual prevalence of fibromyalgia remained consistent from 2016 to 2020: 0.01% (95% CI: 0.01–0.01; Fig. 2; see Table 2, supplemental digital content, http://links.lww.com/PR9/A410). In 2020, prevalence was 0.01% (95% CI: 0.01–0.01) in female individuals and 0.0020% (95% CI: 0.0014–0.0029) in male individuals, resulting in a female-to-male ratio of 4.74 (see Figure 2, supplemental digital content, http://links.lww.com/PR9/A410; see Table 3, supplemental digital content, http://links.lww.com/PR9/A410). Few cases were observed in children aged 0 to <12 years, with a prevalence of 0.0007% (95% CI: 0.0004–0.0012) in 2020 (see Figure 2, supplemental digital content, http://links.lww.com/PR9/A410; see Table 2, supplemental digital content, http://links.lww.com/PR9/A410). Among adolescents (12 to <18 years), prevalence was 0.01% (95% CI: 0.01–0.02) in 2020, with 0.02% (95% CI: 0.02–0.03) in female adolescents and 0.0040% (95% CI: 0.0026–0.0059) in male adolescents (female-to-male ratio of 5.54; see Figure 2, supplemental digital content, http://links.lww.com/PR9/A410; see Table 3, supplemental digital content, http://links.lww.com/PR9/A410). In 2020, prevalence among the pediatric population (0 to <18 years) was 0.00% (95% CI: 0.00–0.01) in the Northeast, 0.01% (95% CI: 0.00–0.01) in the North Central and South regions, and 0.01% (95% CI: 0.01–0.01) in the West (see Figure 3, supplemental digital content, http://links.lww.com/PR9/A410; see Table 4, supplemental digital content, http://links.lww.com/PR9/A410).

#### 3.1.2. Medicaid

**Figure 4.**
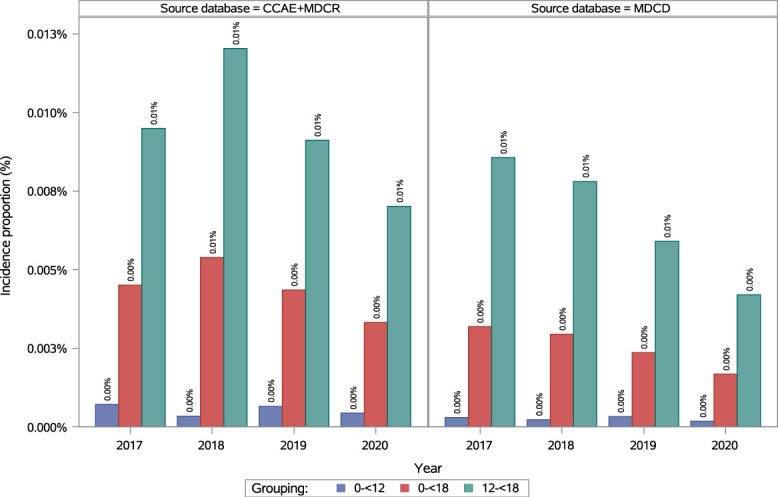
Annual incidence of diagnosed fibromyalgia in the pediatric population in Commercial + Medicare and Medicaid databases. CCAE, Commercial Claims and Encounters database; MDCR, Medicare Supplemental and Coordination of Benefits Database; MDCD, Medicaid Database.

In adults (≥18 years), annual prevalence of fibromyalgia generally decreased over time, from 1.22% (95% CI: 1.20–1.23) in 2016 to 0.90% (95% CI: 0.89–0.91) in 2020 (see Table 2, supplemental digital content, http://links.lww.com/PR9/A410). Prevalence increased with age, with the 50 to <60 years age group presenting the highest annual prevalence throughout the study period (Fig. 1). In 2020, prevalence was 1.33% (95% CI: 1.31–1.34) in female adults and 0.15% (95% CI: 0.14–0.16) in male adults (female-to-male ratio of 8.80; see Figure 1, supplemental digital content, http://links.lww.com/PR9/A410; see Table 3, supplemental digital content, http://links.lww.com/PR9/A410). Among races/ethnicities, Whites had the highest prevalence, at 1.22% (95% CI: 1.20–1.24) in 2020 (see Figure 4, supplemental digital content, http://links.lww.com/PR9/A410; see Table 5, supplemental digital content, http://links.lww.com/PR9/A410).

In the pediatric population (0 to <18 years), the annual prevalence of fibromyalgia was 0.01% (95% CI: 0.01–0.01) in 2016 and 0.003% (95% CI: 0.002–0.003) in 2020 (Fig. 2; see Table 2, supplemental digital content, http://links.lww.com/PR9/A410). In 2020, prevalence was 0.005% (95% CI: 0.004–0.006) in female individuals and 0.001% (95% CI: 0.001–0.001) in male individuals (female-to-male ratio of 5.99; see Figure 2, supplemental digital content, http://links.lww.com/PR9/A410; see Table 3, supplemental digital content, http://links.lww.com/PR9/A410). Among races/ethnicities, Whites had the highest prevalence, at 0.005% (95% CI: 0.004–0.01) in 2020 (see Figure 4, supplemental digital content, http://links.lww.com/PR9/A410; see Table 5, supplemental digital content, http://links.lww.com/PR9/A410). Few fibromyalgia cases were observed in children aged 0 to <12 years, with prevalence of 0.0002% (95% CI: 0.0001–0.0005) in 2020 and 0.0006% (95% CI: 0.0004–0.0010) in 2016. Among adolescents (12 to <18 years), prevalence was 0.02% (95% CI: 0.02–0.02) in 2016 and 0.01% (95% CI: 0.01–0.01) in 2020 (Fig. 2; see Table 2, supplemental digital content, http://links.lww.com/PR9/A410). In 2020, prevalence in adolescents was 0.01% (95% CI: 0.01–0.02) in female adolescents and 0.002% (95% CI: 0.001–0.003) in male adolescents (female-to-male ratio of 7.40; see supplemental digital content, Figure 2, http://links.lww.com/PR9/A410; see Table 3, supplemental digital content, http://links.lww.com/PR9/A410). Among races/ethnicities in the pediatric population (<18 years), Whites had the highest prevalence, at 0.003% (95% CI: 0.002–0.004) in 2020 (see Figure 4, supplemental digital content, http://links.lww.com/PR9/A410; see Table 5, supplemental digital content, http://links.lww.com/PR9/A410).

Annual prevalences of fibromyalgia with ≥1 diagnostic code are detailed in Supplemental digital content (see Tables 6, 7, 8, 9, and 10, http://links.lww.com/PR9/A410).

### 3.2. Annual incidence of diagnosed fibromyalgia with ≥2 diagnostic codes (2017–2020)

#### 3.2.1. Commercial and Medicare

**Figure 5.**
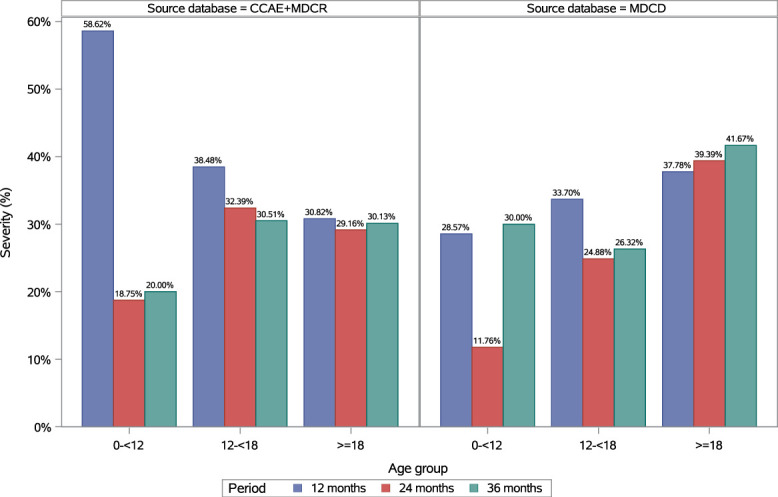
Disease severity of diagnosed fibromyalgia by ethnicities/races in the pediatric and adult populations in Commercial + Medicare and Medicaid databases. CCAE, Commercial Claims and Encounters database; MDCR, Medicare Supplemental and Coordination of Benefits Database; MDCD, Medicaid Database.

In adults (≥18 years), the annual incidence of fibromyalgia slightly decreased from 0.16% (95% CI: 0.16–0.16) in 2017 to 0.12% (95% CI: 0.12–0.12) in 2020 (see Table 10, supplemental digital content, http://links.lww.com/PR9/A410). In 2020, the incidence of fibromyalgia increased with age, peaking in the 50 to <60 years age group at 0.18% (95% CI: 0.18–0.19; Fig. 3; Table 10, supplemental digital content, http://links.lww.com/PR9/A410). Incidence of fibromyalgia was 0.21% (95% CI: 0.21–0.22) in female adults and 0.02% (95% CI: 0.02–0.02) in male adults, resulting in a female-to-male ratio of 9.72 in 2020 (see Figure 5, supplemental digital content, http://links.lww.com/PR9/A410; see Table 11, supplemental digital content, http://links.lww.com/PR9/A410). Among the regions, the highest incidence was observed in the North Central region, ie, 0.18% (95% CI: 0.18–0.19) in 2016 and 0.14% (95% CI: 0.13–0.14) in 2020; see Table 12, supplemental digital content, http://links.lww.com/PR9/A410).

In the pediatric population (0 to <18 years), the annual incidence of fibromyalgia fluctuated over time and slightly decreased from 0.005% (95% CI: 0.004–0.005) in 2017 to 0.003% (95% CI: 0.003–0.004) in 2020 (Fig. 4; see Table 10, supplemental digital content, http://links.lww.com/PR9/A410). In 2020, the annual incidence of fibromyalgia was 0.01% (95% CI: 0.00–0.01) in female individuals and 0.0013% (95% CI: 0.0007–0.0021) in male individuals, resulting in a female-to-male ratio of 4.24 (see Figure 6, supplemental digital content, http://links.lww.com/PR9/A410; see Table 11, supplemental digital content, http://links.lww.com/PR9/A410). Few incident fibromyalgia cases were observed in children aged 0 to <12 years, with an annual incidence of 0.0007% (95% CI: 0.0004–0.0012) in 2017 and 0.0004% (95% CI: 0.0002–0.0010) in 2020. Among adolescents (12 to <18 years), the annual incidence of fibromyalgia was 0.01% (95% CI: 0.01–0.01) in both 2017 and 2020 (Fig. 4; see Table 12, supplemental digital content, http://links.lww.com/PR9/A410). In 2020, incidence in female adolescents was 0.01% (95% CI: 0.01–0.01) and 0.0026% (95% CI: 0.0014–0.0043) in male adolescents, resulting in a female-to-male ratio of 4.54 (see Figure 6, supplemental digital content, http://links.lww.com/PR9/A410; see supplemental digital content, Table 11, http://links.lww.com/PR9/A410). In 2020, incidence among the pediatric population (0 to <18 years) was 0.00% in 2017, 2019 and 2020 and 0.01%. in 2018 Incidence was 0.00% (95% CI: 0.00–0.00) in the Northeast, North Central, and South regions, and 0.01% (95% CI: 0.00–0.01) in the West (see Figure 7, supplemental digital content, http://links.lww.com/PR9/A410; see supplemental digital content, Table 12, http://links.lww.com/PR9/A410).

#### 3.2.2. Medicaid

In adults (≥18 years), the annual incidence of fibromyalgia decreased from 0.31% (95% CI: 0.30–0.31) in 2017 to 0.25% (95% CI: 0.25–0.26) in 2020 (see Table 10, supplemental digital content, http://links.lww.com/PR9/A410). Incidence increased with age, with the 50 to <60 years age group presenting the highest annual incidence throughout the study period, reaching 0.52% (95% CI: 0.50–0.54) in 2020 (Fig. 3; see supplemental digital content, Table 10, http://links.lww.com/PR9/A410). In 2020, incidence was 0.37% (95% CI: 0.36–0.38) in female adults and 0.05% (95% CI: 0.05–0.05) in male adults, resulting in a female-to-male ratio of 7.49 (see supplemental digital content, Figure 5, http://links.lww.com/PR9/A410; see supplemental digital content, Table 11, http://links.lww.com/PR9/A410). Among races/ethnicities, Whites had the highest incidence at 0.35% (95% CI: 0.34–0.36) in 2020 (see supplemental digital content, Figure 8, http://links.lww.com/PR9/A410; see supplemental digital content, Table 13, http://links.lww.com/PR9/A410).

In the pediatric population (0 to <18 years), the annual incidence of fibromyalgia was 0.003% (95% CI: 0.003–0.004) in 2017 and 0.002% (95% CI: 0.001–0.002) in 2020 (Fig. 4; see supplemental digital content, Table 10, http://links.lww.com/PR9/A410). In 2020, incidence was 0.0030% (95% CI: 0.0022–0.0039) in female individuals and 0.0005% (95% CI: 0.0002–0.0009) in male individuals, resulting in a female-to-male ratio of 6.17 (see supplemental digital content, Figure 6, http://links.lww.com/PR9/A410; see supplemental digital content, Table 11, http://links.lww.com/PR9/A410). Few cases were observed in the 0 to <12 years age group, with an incidence of 0.0003% (95% CI: 0.0001–0.0006) in 2017 and 0.0002% (95% CI: 0.0000–0.0004) in 2020. Among adolescents (12 to <18 years), incidence was 0.01% (95% CI: 0.01–0.01) in 2017 and 0.004% (95% CI: 0.003–0.005) in 2020 (Fig. 4; see supplemental digital content, Table 10). In 2020, incidence in female adolescents was 0.01% (95% CI: 0.01–0.01) and 0.0007% (95% CI: 0.0002–0.0017) in male adolescents (female-to-male ratio of 11.17; see supplemental digital content, Figure 6, http://links.lww.com/PR9/A410; see supplemental digital content, Table 11, http://links.lww.com/PR9/A410). Among races/ethnicities in the pediatric population (<18 years), Whites had the highest incidence at 0.003% (95% CI: 0.002–0.004) in 2020 (see supplemental digital content, Figure 8, http://links.lww.com/PR9/A410; see supplemental digital content, Table 13, http://links.lww.com/PR9/A410).

Annual incidences of fibromyalgia with ≥1 diagnostic code are detailed in supplemental digital content (see Tables 14, 15, 16, and 17, http://links.lww.com/PR9/A410).

### 3.3. Severity inferred from consultations and medications among individuals with incident fibromyalgia (≥2 diagnosis codes)

#### 3.3.1. Commercial and Medicare

In adults (≥18 years) with incident fibromyalgia and ≥12 (n = 41,802), ≥24 (n = 24,527) and ≥36 (n = 13,340) months of follow-up, the proportions described as having severe fibromyalgia were 30.8%, 29.2% and 30.1%, respectively (Fig. 5; see supplemental digital content, Table 18, http://links.lww.com/PR9/A410).

In adolescents (12 to <18 years) with incident fibromyalgia and ≥12 (n = 356), ≥24 (n = 213) and ≥36 (n = 118) months of follow-up, the proportions described as having severe fibromyalgia were 38.5%, 32.4% and 30.5%, respectively (Fig. 5; see supplemental digital content, Table 19, http://links.lww.com/PR9/A410).

#### 3.3.2. Medicaid

In adults (≥18 years) with incident fibromyalgia and ≥12 (n = 23,758), ≥24 (n = 15,965), and ≥36 (n = 10,134) months of follow-up, the proportions described as having severe fibromyalgia were 37.8%, 39.4%, and 41.7%, respectively (Fig. 5; see supplemental digital content, Table 18, http://links.lww.com/PR9/A410).

In adolescents (12 to <18 years) with incident fibromyalgia and ≥12 (n = 276), ≥24 (n = 209) and ≥36 (n = 133) months of follow-up, the proportions described as having severe fibromyalgia were 33.7%, 24.9%, and 26.3%, respectively (Fig. 5; see supplemental digital content, Table 19, http://links.lww.com/PR9/A410).

Severity inferred from consultations and medication among individuals with fibromyalgia (≥1 diagnostic code) is detailed in supplemental digital content (see Tables 20 and 21, http://links.lww.com/PR9/A410).

### 3.4. Comorbidities

#### 3.4.1. Commercial and Medicare, and Medicaid

In adults with incident fibromyalgia at baseline (12 months before index date) and ≥12, ≥24, and ≥36 months of follow-up, the most common comorbidities along with CCI categories are provided in supplemental digital content (see Tables 22 [≥2 diagnosis codes] and 23 [≥1 diagnosis code], http://links.lww.com/PR9/A410).

## 4. Discussion

Epidemiology data on diagnosed fibromyalgia in the US are scant and outdated. This is the largest population-based study reporting updated estimates of the diagnosed annual prevalence, incidence, and severity of fibromyalgia in the US, leveraging 3 large claims databases.

Fibromyalgia incidence and prevalence in this study reflected only the subset of individuals who sought medical care and received a morbidity code by clinicians, rather than the true population burden.^10,17^ Incidence did not represent true condition onset because patients often experienced symptoms for years before diagnosis; those ultimately diagnosed might have had persistent multisystem symptoms and frequent healthcare contact.^5,47^ Diagnostic patterns might have been influenced by clinician recognition,^2,32,41^ and increased diagnosis among individuals with rheumatic comorbidities.^14^

The annual prevalence of fibromyalgia in adults was approximately twice as high in Medicaid as in Commercial + Medicare. This may reflect socioeconomic differences, as Medicaid is a joint federal and state program covering people with limited income and resources. A United Kingdom (UK) based study reported higher fibromyalgia prevalence in populations with low socioeconomic status assisted by the public primary healthcare system.^3^ Socioeconomic disadvantage has also been linked to chronic widespread pain (a prominent fibromyalgia symptom).^26^ By contrast, fibromyalgia itself imposes substantial health and socioeconomic burdens on affected individuals, their families, and society (Amris et al., 2024); thus, fibromyalgia may contribute to lower socioeconomic status by limiting individuals' ability to work.

Prevalence estimates in adults from this study are consistent with published medical chart review studies, ranging from 1.07% (95% CI: 1.06–1.08)^36^ in Spain to 1.12% (95% CI: 1.05–1.18)^43^ in the US. Lower prevalence has been reported in primary care records in the UK (0.36% [95% CI: 0.32–0.40]).^28^ By contrast, US cross-sectional survey studies^16,18,43,44^ have reported a higher prevalence of fibromyalgia in the general population, ranging from 1.75%^44^ to 5.30%.^43^ This estimate discrepancy between data sources likely reflects underdiagnosis and underreporting of fibromyalgia, and the proportion of individuals who experience symptoms or do not receive diagnostic code by the clinicians.

Consistent with the previously published studies, prevalence was substantially higher in female individuals than male individuals (female-to-male ratio: ∼14 in Commercial + Medicare and ∼9 in Medicaid).^6,16,21,36,43,44,48^ Women are more likely to be coded with fibromyalgia than men, reflecting both genuine prevalence differences and potential gender bias in symptom recognition.^36^

Prevalence increased with age, peaking at 70 to <80 years in Commercial + Medicare and 50 to <60 years in Medicaid, similar to cross-sectional survey studies in the US reporting peaks at 50 to <60 years,^45^ 70 to <80 years,^24^ and 75 to <85 years.^6^ The earlier peak in Medicaid may have reflected transitions to Medicaid among individuals whose fibromyalgia began at an earlier age and reduced their work capacity, whereas the later peak in Commercial and Medicare aligns with a second rise in incidence at older ages.

In pediatric populations, prevalence was higher in adolescents (12 to <18 years) than in younger children (0 to <12 years) in Commercial + Medicare and Medicaid, with very few cases observed under 6 years, likely due to the limited ability of children <6 years to express symptoms. Previously reported estimated prevalence of juvenile primary fibromyalgia syndrome using questionnaires ranges from 1.2% in Mexico to 6.2% in Israel.^9^

Whites had the highest annual prevalence and incidence. Lower estimates in minority groups could be attributed to clinicians distrusting racial/ethnic minority patients reporting pain.^19^

Annual incidence estimates in adults from this study are aligned with published studies.^8,11,12,36^ Similar to prevalence, the annual incidence of fibromyalgia was approximately twice as high in Medicaid as in Commercial + Medicare, likely reflecting socioeconomic factors. No US studies were found reporting the incidence of fibromyalgia in the general population; however, there was a study reporting cumulative incidence (1998–2002) in male adults (5.99 per 1000 person-years) and female adults (10.91 per 1000 person-years) aged <65 years using a claims database.^46^ In the US, the calculated annual incidence of fibromyalgia in older adults treated for pain within 60 days on or after any diagnostic claim for fibromyalgia, diabetic peripheral neuropathy, or post-herpetic neuralgia was 0.48%^20^; among women ≥25 years old with self-reported fibromyalgia from 1976 to 2002, cumulative incidence was 4.3% and mean annual incidence.^8^ In Spain, annual incidence of fibromyalgia in the adult general population was 0.10% in 2017.^36^ In the Netherlands, the cumulative incidence of fibromyalgia over the study period of 2.4 years was 0.5%, the calculated mean annual incidence, and the incidence rate was 24 per 10,000 person-years.^12^ In the UK, the average annual incidence of fibromyalgia was 0.0333% (95% CI: 0.0328%–0.0338%).^11^

Incidence was higher in female adults than in male adults (female-to-male ratio: ∼10 in the Commercial + Medicare database and ∼7 in the Medicaid database). Similarly, a previous study from the UK reported that women had a 6-fold higher incidence of fibromyalgia than men.^11^

In 2020, the annual incidence increased with age up to 50 to <60 years, then declined, with a secondary peak at 70 to <80 years in Commercial + Medicare and Medicaid. Similarly, a study from the UK reported that fibromyalgia incidence rates peaked between ages 40 and 59 years.^11^

Incidence of fibromyalgia was higher in those aged 12 to <18 years than in those aged 0 to <12 years; this may also be related to the reduced ability for children <6 years to express symptoms. Similar findings were reported in the US; cumulative incidence (1998–2002) was found to be higher in those aged 10 to 19 years than in those aged <10 years.^46^

The proportion of adults with severe fibromyalgia at ≥36 months of follow-up was higher in Medicaid (41.7%) compared with Commercial + Medicare (30.1%), consistent with evidence that people with fibromyalgia and lower socioeconomic status experience greater symptom severity and functional impairment.^15^ Some individuals may move from Commercial into Medicaid following reduced income or ability to work, and disability-related aid categories recorded in Medicaid may also contribute to the higher severity.

Our severity algorithm classified patients as severe if they met any of several criteria (see Table 1, supplemental digital content, http://links.lww.com/PR9/A410). By contrast, the ACR 2016 criteria describe severity based on somatic symptoms. Fibromyalgia severity reported in this study aligns with published literature. In the US, the proportion of adult patients with severe fibromyalgia ranged from 40.3%^38^ to 66%.^23^ A higher proportion was reported when a validated questionnaire was used to categorize severity level^23,30,37^ compared with self-report.^38^ In Europe, proportions varied from 38.8% (Italy^35^) to 48.4% (Spain^34^). No data were available for pediatric populations in Europe and the US. The high proportion of severity observed in this study and previously published studies highlights significant unmet needs among people with fibromyalgia. Our definition of severity, which links fibromyalgia to increased consumption of medical resources, underscores that fibromyalgia also imposes substantial burdens on healthcare systems and communities.

Our study had some limitations. The study population was limited to patients with Commercial, Medicare, or Medicaid insurance; therefore, the results may not be representative of the entire US population. Two diagnostic codes were used to confirm prevalence and incidence, which may underestimate fibromyalgia among individuals who do not return for care within a year of their first diagnosis. Therefore, prevalence and incidence estimates based on ≥1 diagnostic code were also reported. In clinical practice, the ACR 2016 criteria can be used to define fibromyalgia severity, which cannot be operationalized in studies using electronic health record data. Hence, we described the individuals based on consultations with physicians and prescriptions; however, consultations may be influenced by the health-seeking behavior of the individuals, and prescription adherence is unknown. Fibromyalgia severity was determined in individuals with ≥12, ≥24, and ≥36 months of follow-up, which may have introduced a selection bias as individuals with shorter follow-up were not included.

## 5. Conclusion

This observational cohort study provides an up-to-date description of the prevalence, incidence, and severity of fibromyalgia. This is the largest US study using the MarketScan databases, solely reporting epidemiology of fibromyalgia in the general population, notably including pediatric populations. Epidemiological knowledge is a crucial first step for effective healthcare management of patients with fibromyalgia. Further studies are required to validate algorithms for identifying severe fibromyalgia in real-world settings.

## Disclosures

The authors have no conflict of interest to declare.

## Supplemental digital content

Supplemental digital content associated with this article can be found online at http://links.lww.com/PR9/A410.

## Supplementary Material
