# Supplementary material for: Prevalence and incidence of fibromyalgia and associated severity in the United States using MarketScan
Source: Pain Rep. 2026 Jun 1;11(4):e1451. doi: 10.1097/PR9.0000000000001451 (PMC13229459; doi:10.1097/PR9.0000000000001451)
Supplement: Supplementary file 1 [file painreports-11-e1451-s001.pdf]

SUPPLEMENTARY MATERIALS

Supplementary Figures

Supplementary Figure 1: Annual prevalence of diagnosed fibromyalgia by sex in the adult population in Commercial + Medicare and Medicaid databases

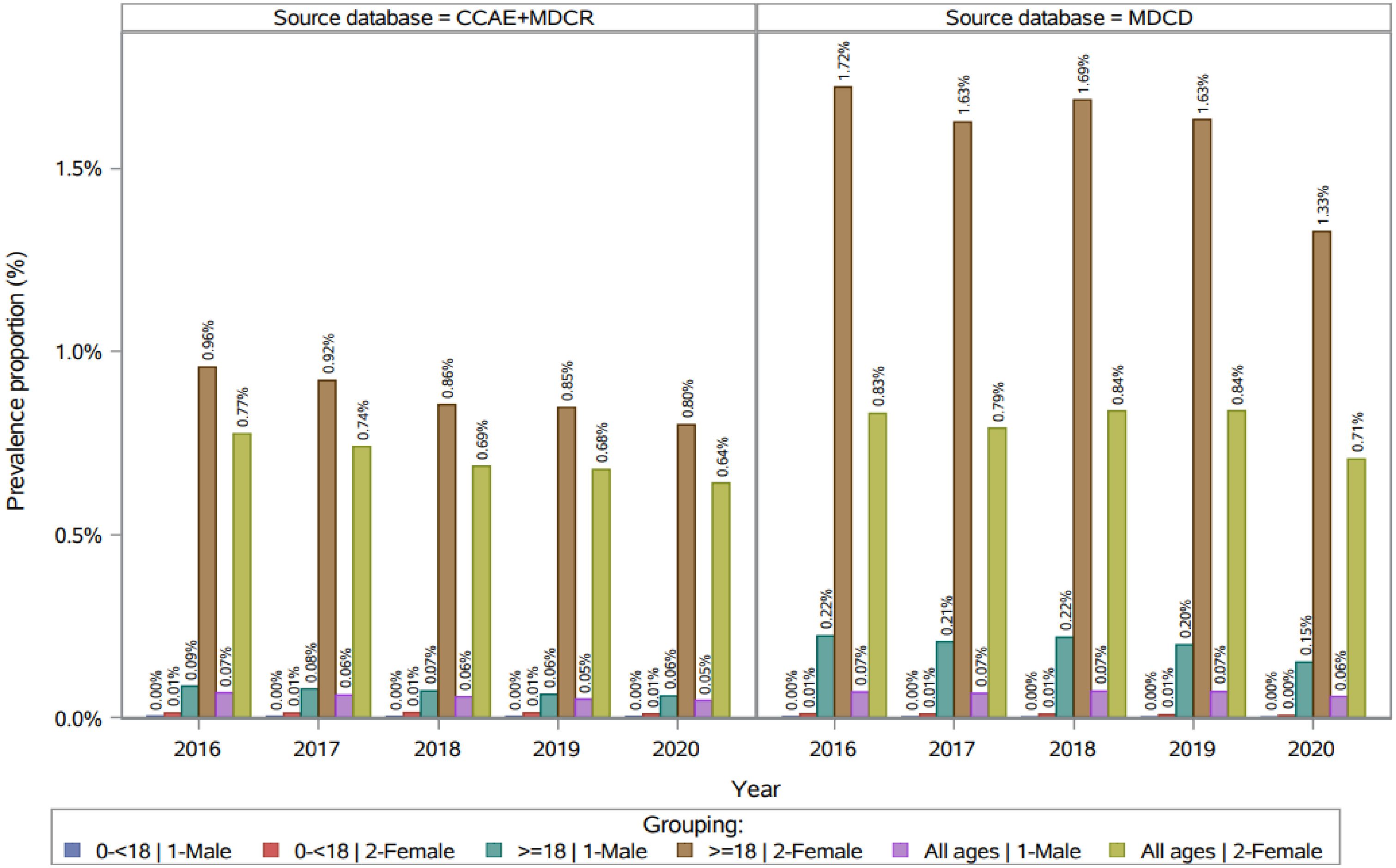

CCAE=Commercial Claims and Encounters database; MDCR= Medicare Supplemental and Coordination of Benefits Database; MDCD=Medicaid Database

Supplementary Figure 2: Annual prevalence of diagnosed fibromyalgia by sex in the pediatric population in Commercial + Medicare and Medicaid databases

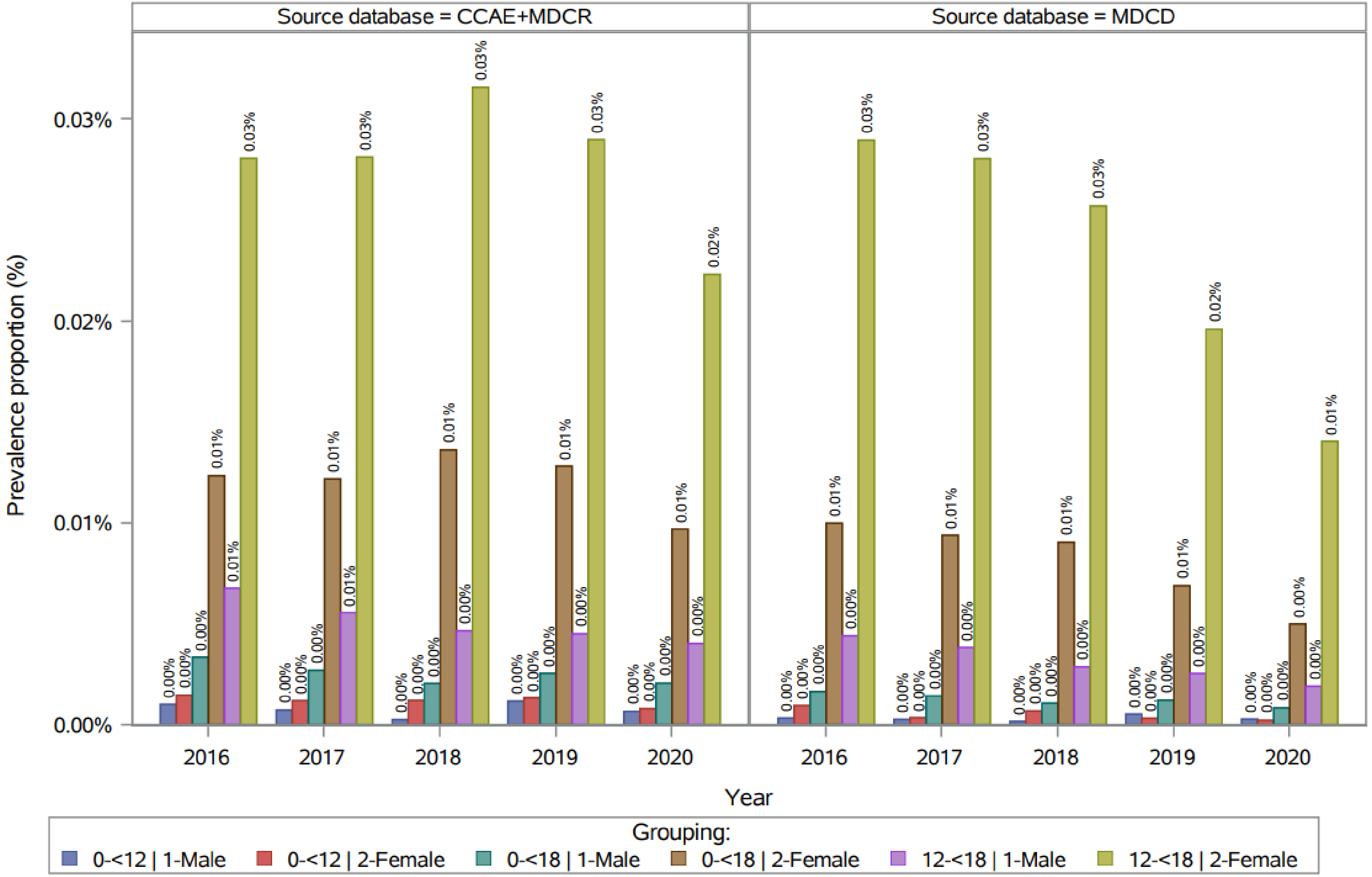

CCAE=Commercial Claims and Encounters database; MDCR= Medicare Supplemental and Coordination of Benefits Database; MDCD=Medicaid Database

Supplementary Figure 3: Annual prevalence of diagnosed fibromyalgia by geographical regions in the pediatric and adult populations in Commercial + Medicare databases

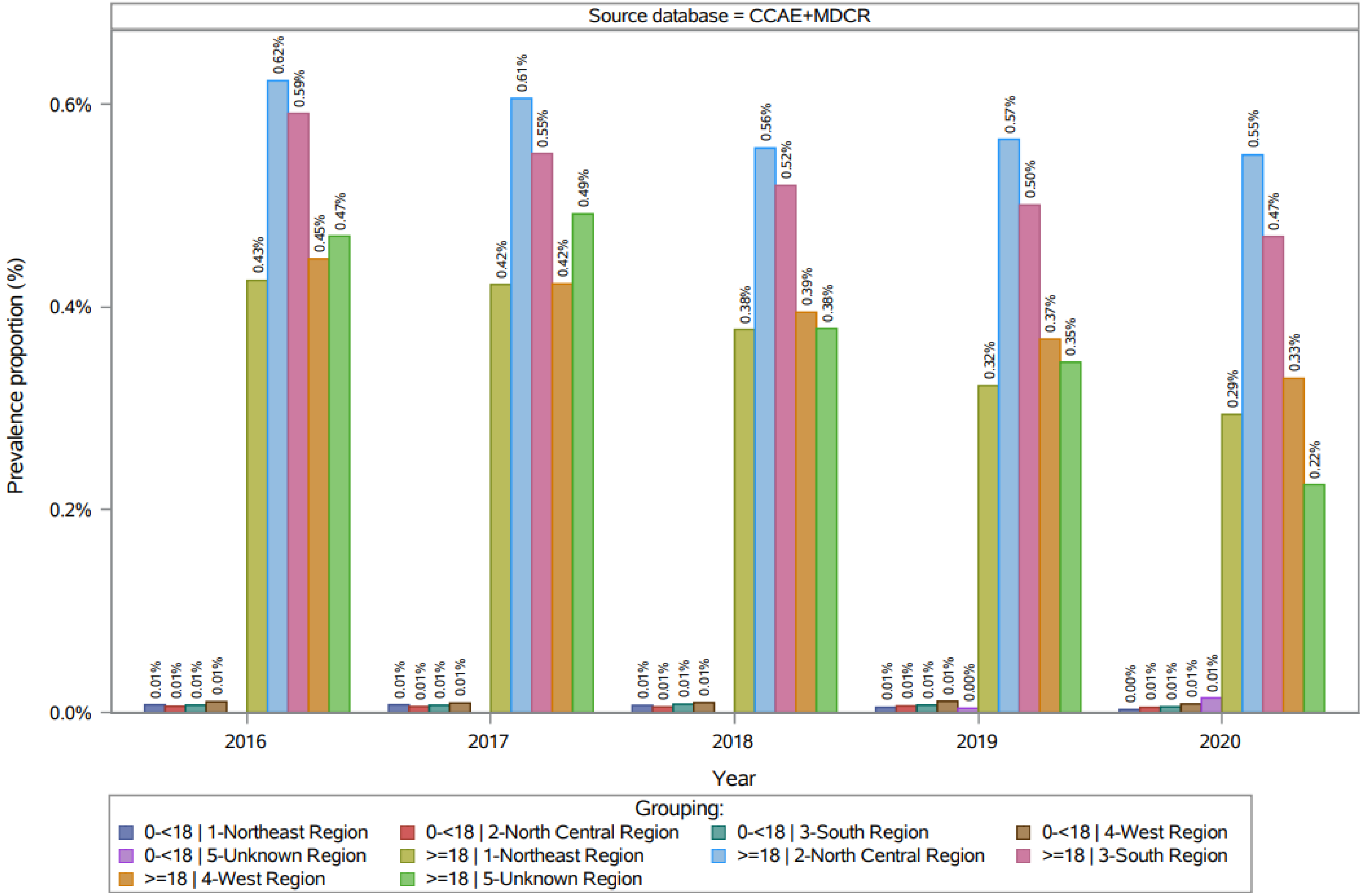

CCAE=Commercial Claims and Encounters database; MDCR= Medicare Supplemental and Coordination of Benefits Database

Supplementary Figure 4: Annual prevalence of diagnosed fibromyalgia by ethnicities/races in the pediatric and adult populations in the Medicaid database

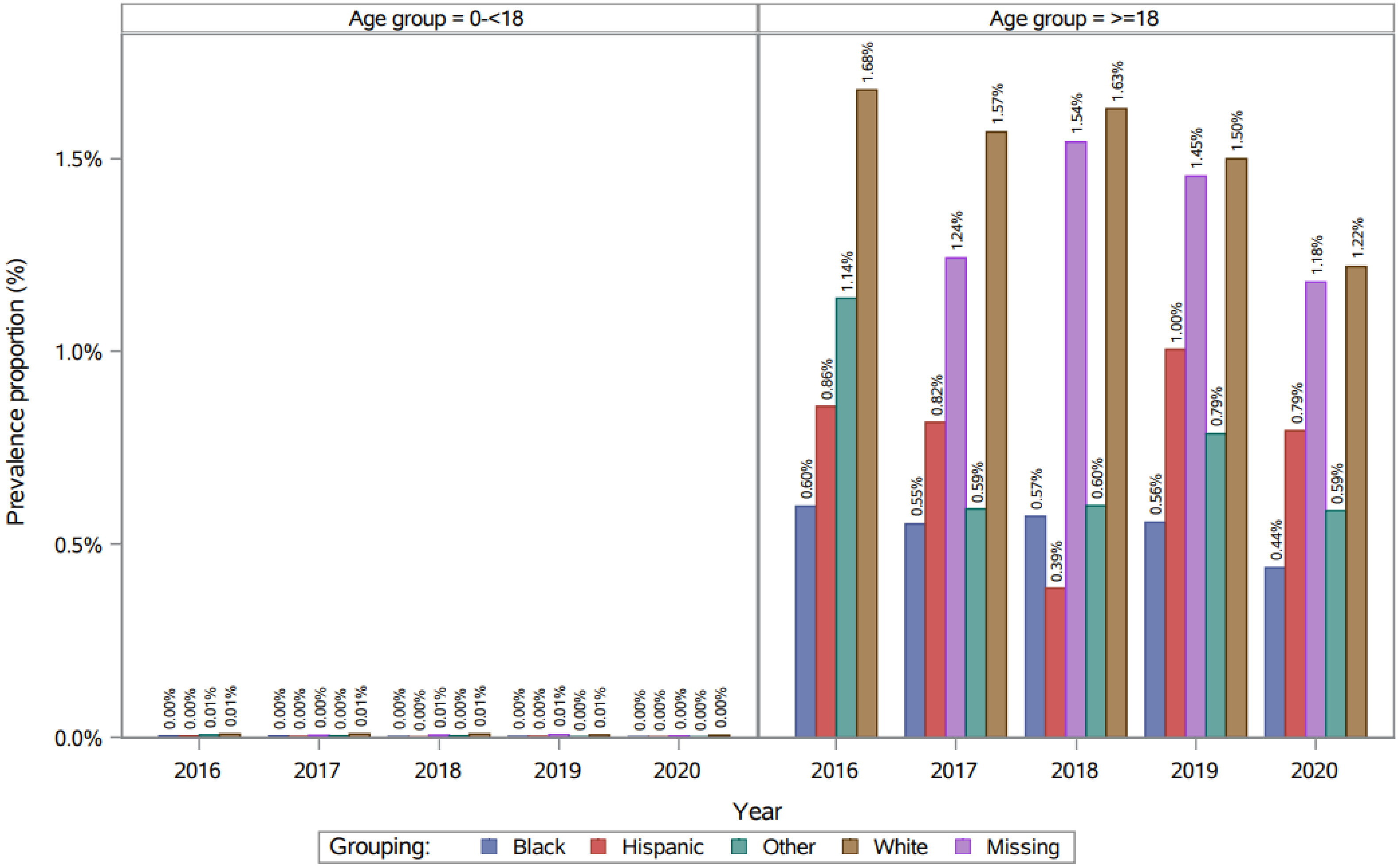

Supplementary Figure 5: Annual incidence of diagnosed fibromyalgia by age and sex in Commercial + Medicare and Medicaid databases

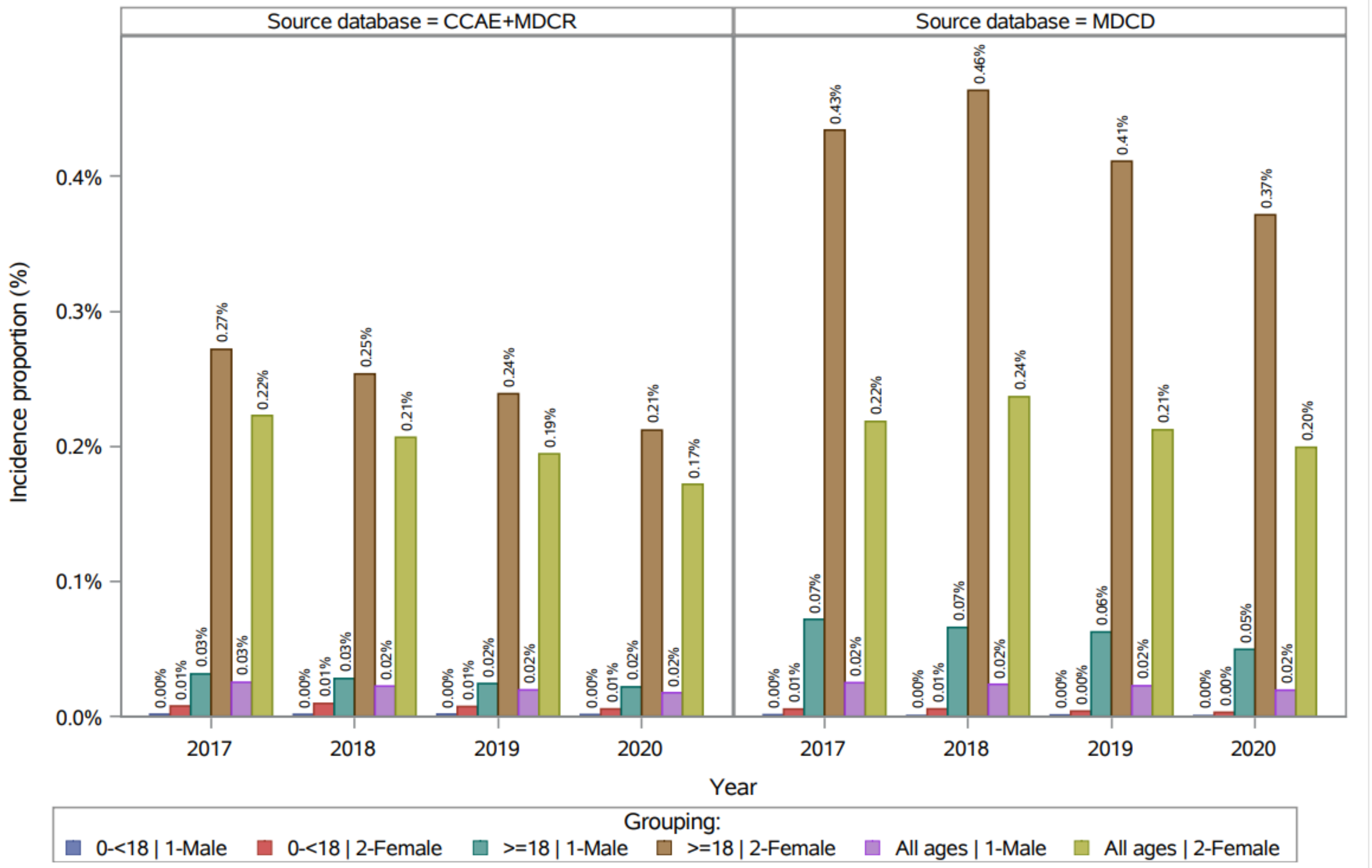

CCAE=Commercial Claims and Encounters database; MDCR= Medicare Supplemental and Coordination of Benefits Database; MDCD=Medicaid Database

Supplementary Figure 6: Annual incidence of diagnosed fibromyalgia in the pediatric population by sex in Commercial + Medicare and Medicaid databases

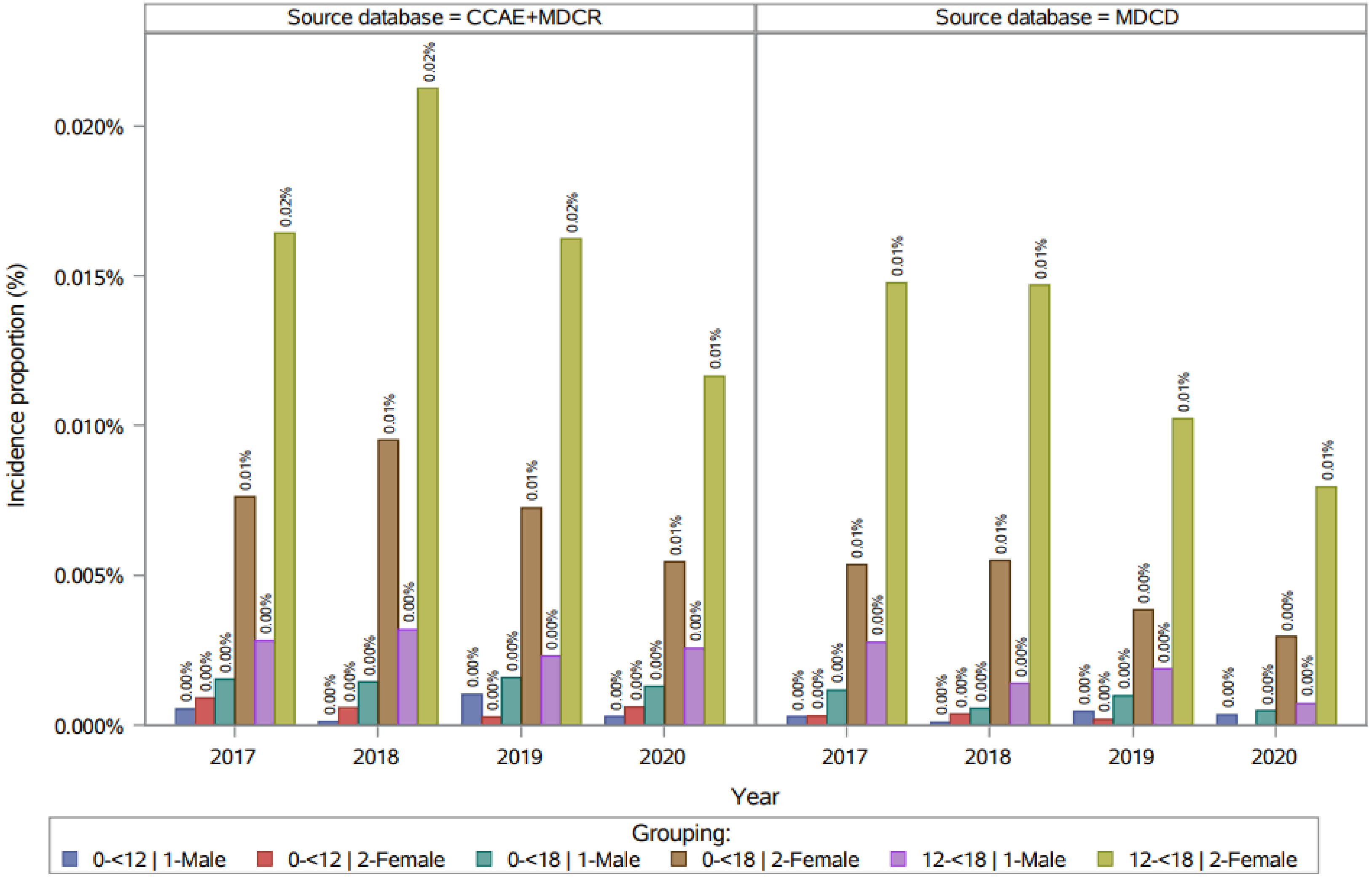

CCAE=Commercial Claims and Encounters database; MDCR= Medicare Supplemental and Coordination of Benefits Database; MDCD=Medicaid Database

Supplementary Figure 7: Annual incidence of diagnosed fibromyalgia by geographical regions in the pediatric and adult populations in Commercial + Medicare databases

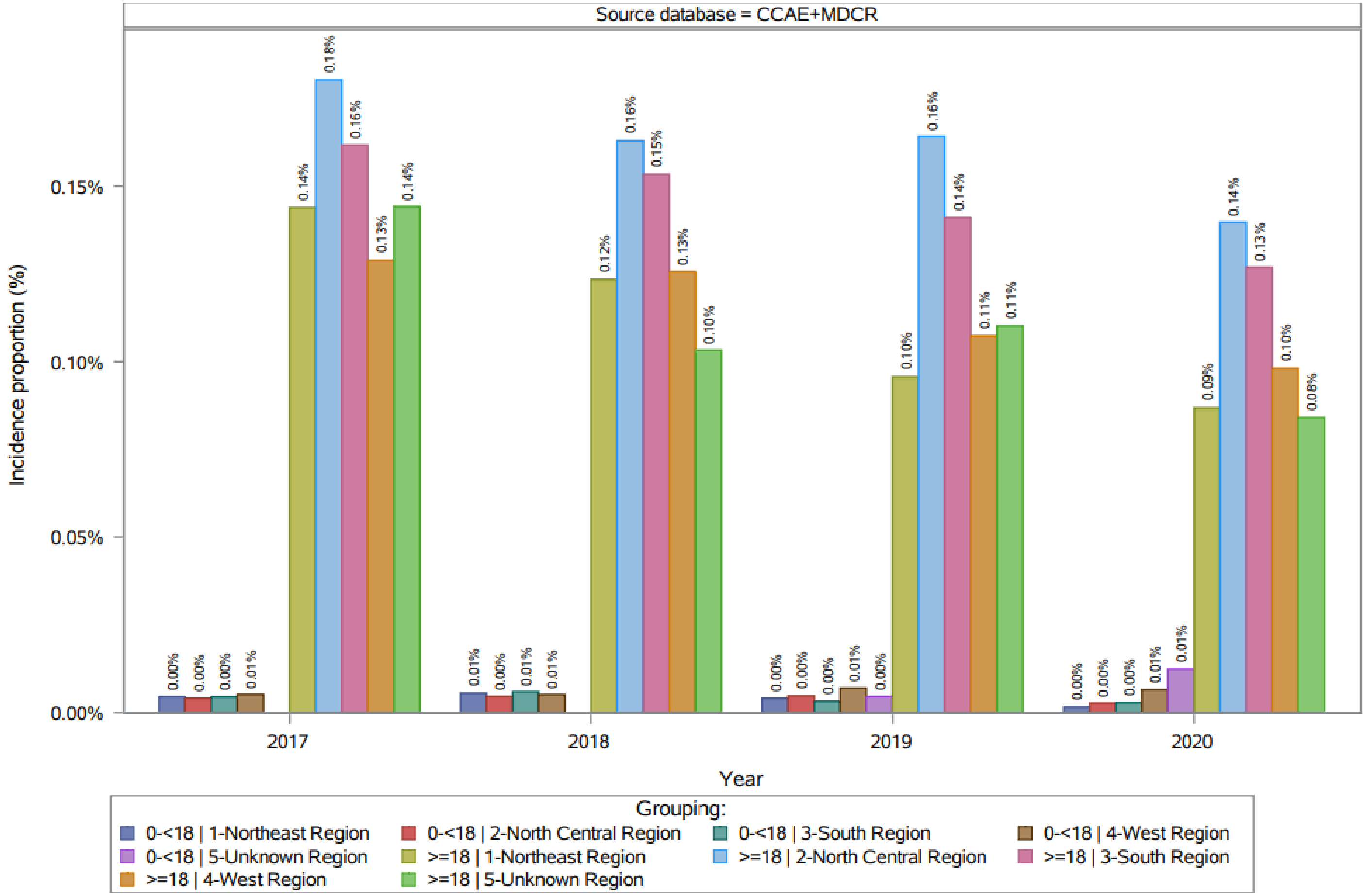

CCAE=Commercial Claims and Encounters database; MDCR= Medicare Supplemental and Coordination of Benefits Database

Supplementary Figure 8: Annual incidence of diagnosed fibromyalgia by ethnicities/races in the pediatric and adult populations in the Medicaid database

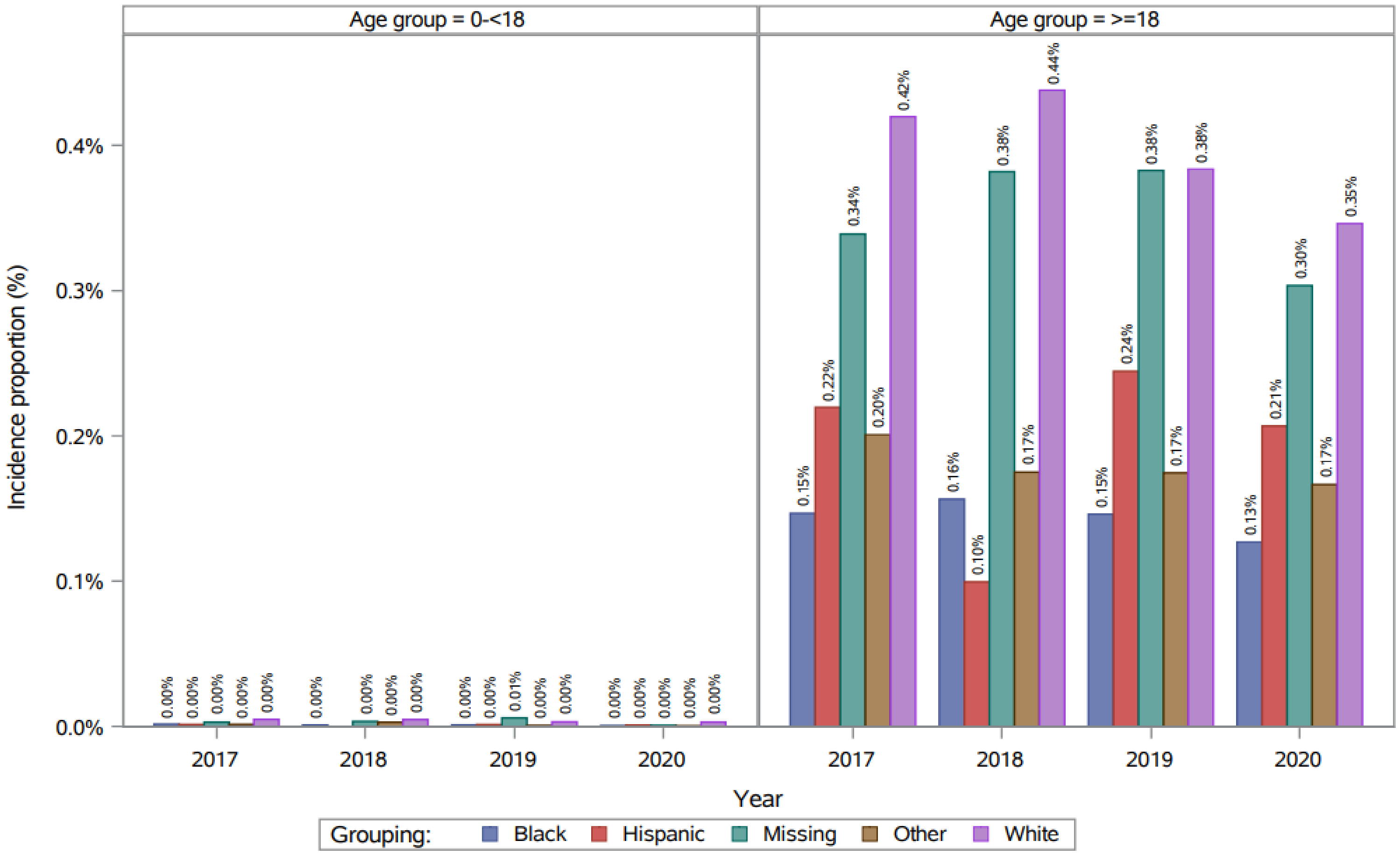

Supplementary Tables

Supplementary Table 1. Criteria for inferring severe fibromyalgia

**Severe fibromyalgia criteria**	**Definition**
>5, >10, or >15 diagnostic codes/claims within 12, 24, or 36 months of first diagnosis, respectively	People with >5, >10, or >15 fibromyalgia diagnosis codes/claims on different dates within 12, 24, or 36 months after the index date
Incident events of disability in people with fibromyalgia	People with any ICD-9 or -10 disability diagnosis codes
Incident events of hospitalization at pain clinics in people with fibromyalgia	Any medical claim for hospitalization at pain clinics
Incident events of people seen at pain clinics	Any medical claim for people seen at pain clinics
≥5 concomitant predefined medications potentially prescribed for fibromyalgia treatment with fibromyalgia approved treatments in the US- pregabalin, duloxetine, milnacipran	Concomitant medication use was defined as any overlap of ≥5 fibromyalgia medications differentiated by generic names<br>Medications included were:<br>Selective serotonin reuptake inhibitors<br>Fluoxetine, sertraline, paroxetine, citalopram, escitalopram, fluvoxamine<br>Serotonin-norepinephrine reuptake inhibitors<br>Duloxetine, milnacipran, venlafaxine<br>Tricyclic antidepressants<br>Amitriptyline, nortriptyline<br>Anticonvulsants<br>Pregabalin, gabapentin<br>Muscle relaxants<br>Cyclobenzaprine<br>Analgesics<br>Hydrocodone/acetaminophen, oxycodone/acetaminophen, tramadol, oxycodone<br>NSAIDs and COX inhibitors<br>Ibuprofen, naproxen, diclofenac, meloxicam, celecoxib<br>Sedatives, hypnotics and anxiolytics<br>Diazepam, zolpidem, trazodone

COX=Cyclo-Oxygenase; ICD=International Classification of Diseases; NSAIDs=Non-Steroidal Anti-Inflammatory Drugs

Supplementary Table 2: Annual prevalence of diagnosed fibromyalgia (≥2 diagnosis codes) by age groups

		2016		2017		2018		2019		2020	
**Age**	**Statistic**	**CCAE+MDCR**	**MDCD**	**CCAE+MDCR**	**MDCD**	**CCAE+MDCR**	**MDCD**	**CCAE+MDCR**	**MDCD**	**CCAE+MDCR**	**MDCD**
All ages	% (95% CI)	0.44 (0.4-0.44)	0.49 (0.49-0.50)	0.41 (0.41-0.42)	0.47 (0.46-0.47)	0.38 (0.38-0.38)	0.50 (0.49-0.50)	0.37 (0.37-0.37)	0.50 (0.49-0.50)	0.35 (0.35-0.35)	0.42 (0.41-0.42)
	n	90,530	37,185	79,965	35,661	72,923	31,085	55,188	35,652	51,012	34,298
	N	20,778,989	7,542,183	19,377,240	7,616,988	19,149,305	6,248,097	14,894,447	7,188,437	14,581,615	8,209,563
0-<2	% (95% CI)	0.0016 (0.0003-0.0045)	0.0004 (0.0000-0.0020)	0.0000 (0.0000-0.0020)	0.0000 (0.0000-0.0013)	0.0000 (0.0000-0.0020)	0.0000 (0.0000-0.0017)	0.0000 (0.0000-0.0027)	0.0000 (0.0000-0.0016)	0.0000 (0.0000-0.0027)	0.0004 (0.0000-0.0020)
	n	3	1	0	0	0	0	0	0	0	1
	N	192,782	275,338	186,628	275,260	181,968	214,376	137,976	232,280	135,103	275,345
2-<6	% (95% CI)	0.0004 (0.0001-0.0010)	0.0002 (0.0000-0.0007)	0.0002 (0.0000-0.0009)	0.0001 (0.0000-0.0005)	0.0004 (0.0001-0.0011)	0.0000 (0.0000-0.0004)	0.0002 (0.0000-0.0009)	0.0002 (0.0000-0.0008)	0.0002 (0.0000-0.0009)	0.0002 (0.0000-0.0007)
	n	3	2	2	1	3	0	1	2	1	2
	N	837,271	1,093,157	805,744	1,087,197	804,695	873,261	617,286	959,488	600,929	1,033,527
6-<12	% (95% CI)	0.0017 (0.0011-0.0025)	0.0010 (0.0005-0.0015)	0.0015 (0.0009-0.0023)	0.0005 (0.0002-0.0009)	0.0010 (0.0005-0.0017)	0.0008 (0.0004-0.0014)	0.0020 (0.0013-0.0030)	0.0006 (0.0003-0.0012)	0.0011 (0.0006-0.0020)	0.0003 (0.0001-0.0006)
	n	25	16	21	8	14	10	22	9	12	4
	N	1,501,203	1,680,020	1,424,573	1,672,837	1,407,714	1,332,984	1,094,124	1,461,830	1,061,422	1,584,642
0-<12	% (95% CI)	0.0012 (0.0008-0.0017)	0.0006 (0.0004-0.0010)	0.0010 (0.0006-0.0014)	0.0003 (0.0001-0.0006)	0.0007 (0.0004-0.0011)	0.0004 (0.0002-0.0008)	0.0012 (0.0008-0.0019)	0.0004 (0.0002-0.0007)	0.0007 (0.0004-0.0012)	0.0002 (0.0001-0.0005)
	n	31	19	23	9	17	10	23	11	13	7
	N	2,531,256	3,048,515	2,416,945	3,035,294	2,394,377	2,420,621	1,849,386	2,653,598	1,797,454	2,893,514

		2016		2017		2018		2019		2020	
**Age**	**Statistic**	**CCAE+MDCR**	**MDCD**	**CCAE+MDCR**	**MDCD**	**CCAE+MDCR**	**MDCD**	**CCAE+MDCR**	**MDCD**	**CCAE+MDCR**	**MDCD**
12-<18	% (95% CI)	0.02 (0.02-0.02)	0.02 (0.01-0.02)	0.02 (0.01-0.02)	0.02 (0.01-0.02)	0.02 (0.02-0.02)	0.01 (0.01-0.02)	0.02 (0.01-0.02)	0.01 (0.01-0.01)	0.01 (0.01-0.02)	0.01 (0.01-0.01)
	n	300	237	276	229	294	169	216	148	164	119
	N	1,743,482	1,455,064	1,661,043	1,473,919	1,647,781	1,218,432	1,309,533	1,373,394	1,264,884	1,530,994
0-<18	% (95% CI)	0.01 (0.01-0.01)	0.01 (0.01-0.01)	0.01 (0.01-0.01)	0.01 (0.00-0.01)	0.01 (0.01-0.01)	0.0049 (0.0042-0.0057)	0.01 (0.01-0.01)	0.0039 (0.0034-0.0046)	0.01 (0.00-0.01)	0.0028 (0.0024-0.0034)
	n	331	256	299	238	311	179	239	159	177	126
	N	4,274,738	4,503,579	4,077,988	4,509,213	4,042,158	3,639,053	3,158,919	4,026,992	3,062,338	4,424,508
≥18	% (95% CI)	0.55 (0.54-0.55)	1.22 (1.20-1.23)	0.52 (0.52-0.52)	1.14 (1.13-1.15)	0.48 (0.48-0.48)	1.18 (1.17-1.20)	0.47 (0.46-0.47)	1.12 (1.11-1.13)	0.44 (0.44-0.45)	0.90 (0.89-0.91)
	n	90,199	36,929	79,666	35,423	72,612	30,906	54,949	35,493	50,835	34,172
	N	16,504,251	3,038,604	15,299,252	3,107,775	15,107,147	2,609,044	11,735,528	3,161,445	11,519,277	3,785,055
18-<40	% (95% CI)	0.20 (0.20-0.20)	0.62 (0.61-0.63)	0.19 (0.19-0.19)	0.56 (0.55-0.57)	0.18 (0.18-0.18)	0.56 (0.54-0.57)	0.17 (0.17-0.17)	0.51 (0.50-0.52)	0.15 (0.15-0.16)	0.39 (0.38-0.40)
	n	12,186	12,307	11,120	11,250	10,606	9,366	8,131	9,999	7,096	9,499
	N	6,088,855	1,977,530	5,851,418	2,019,808	5,941,650	1,684,069	4,790,328	1,969,922	4,596,179	2,419,040
40-<50	% (95% CI)	0.63 (0.62-0.63)	2.42 (2.38-2.47)	0.61 (0.60-0.62)	2.28 (2.23-2.32)	0.58 (0.57-0.59)	2.35 (2.30-2.39)	0.57 (0.56-0.58)	2.11 (2.08-2.16)	0.51 (0.50-0.52)	1.73 (1.69-1.76)
	n	19,902	11,266	18,620	10,854	17,715	9,415	13,689	10,513	11,604	10,324
	N	3,182,149	465,342	3,051,515	477,078	3,061,095	401,316	2,419,720	497,084	2,282,837	597,621

		2016		2017		2018		2019		2020	
**Age**	**Statistic**	**CCAE+MDCR**	**MDCD**	**CCAE+MDCR**	**MDCD**	**CCAE+MDCR**	**MDCD**	**CCAE+MDCR**	**MDCD**	**CCAE+MDCR**	**MDCD**
50-<60	% (95% CI)	0.83 (0.82-0.84)	2.48 (2.43-2.53)	0.80 (0.79-0.81)	2.41 (2.37-2.46)	0.75 (0.75-0.76)	2.56 (2.51-2.62)	0.74 (0.73-0.75)	2.44 (2.39-2.49)	0.68 (0.67-0.69)	2.10 (2.06-2.14)
	n	30,752	10,221	27,935	10,061	26,121	9,003	19,840	10,535	17,305	10,049
	N	3,713,033	411,900	3,489,291	416,834	3,462,674	351,044	2,683,239	431,721	2,527,364	478,394
60-<70	% (95% CI)	0.87 (0.86-0.88)	1.88 (1.82-1.95)	0.82 (0.81-0.83)	1.79 (1.73-1.85)	0.74 (0.72-0.75)	1.94 (1.87-2.01)	0.73 (0.72-0.74)	1.88 (1.82-1.93)	0.70 (0.69-0.71)	1.62 (1.57-1.67)
	n	20,009	3,033	16,689	3,131	14,324	3,009	10,646	4,031	10,322	3,879
	N	2,299,949	161,079	2,032,407	174,917	1,948,744	155,415	1,458,030	214,798	1,475,150	240,174
70-<80	% (95% CI)	0.76 (0.74-0.78)	0.68 (0.55-0.83)	0.76 (0.73-0.78)	0.87 (0.72-1.04)	0.67 (0.65-0.70)	0.87 (0.71-1.05)	0.82 (0.79-0.86)	1.17 (1.05-1.30)	0.84 (0.81-0.86)	1.10 (0.99-1.22)
	n	5,471	93	4,040	118	2,933	108	2,035	343	3,412	345
	N	721,425	13,697	533,952	13,595	435,589	12,426	246,856	29,386	408,061	31,312
≥80	% (95% CI)	0.38 (0.36-0.39)	0.10 (0.05-0.19)	0.37 (0.35-0.39)	0.16 (0.07-0.31)	0.35 (0.33-0.38)	0.10 (0.03-0.24)	0.44 (0.41-0.48)	0.39 (0.30-0.49)	0.48 (0.45-0.51)	0.41 (0.32-0.51)
	n	1,879	9	1,262	9	913	5	608	72	1,096	76
	N	498,840	9,056	340,669	5,543	257,395	4,774	137,355	18,534	229,686	18,514

CCAE=Commercial Claims and Encounters database; MDCR= Medicare Supplemental and Coordination of Benefits Database; MDCD=Medicaid Database

Supplementary Table 3: Annual prevalence of diagnosed fibromyalgia (≥2 diagnosis codes) by age groups and sex

Age	Sex	Statistic	2016		2017		2018		2019		2020	
			CCAE+MDCR	MDCD	CCAE+MDCR	MDCD	CCAE+MDCR	MDCD	CCAE+MDCR	MDCD	CCAE+MDCR	MDCD
All ages	Male	% (95% CI)	0.07 (0.07-0.07)	0.07 (0.07-0.07)	0.06 (0.06-0.06)	0.07 (0.06-0.07)	0.06 (0.05-0.06)	0.07 (0.07-0.07)	0.05 (0.05-0.05)	0.07 (0.07-0.07)	0.05 (0.04-0.05)	0.06 (0.05-0.06)
		n	6,728	2,323	5,724	2,237	5,245	1,978	3,634	2,257	3,302	2,080
		N	9,961,085	3,346,491	9,347,153	3,389,741	9,288,753	2,773,596	7,288,876	3,203,462	7,133,603	3,645,712
	Female	% (95% CI)	0.77 (0.77-0.78)	0.83 (0.82-0.84)	0.74 (0.73-0.75)	0.79 (0.78-0.80)	0.69 (0.68-0.69)	0.84 (0.83-0.85)	0.68 (0.67-0.68)	0.84 (0.83-0.85)	0.64 (0.63-0.65)	0.71 (0.70-0.71)
		n	83,802	34,862	74,241	33,424	67,678	29,107	51,554	33,395	47,710	32,218
		N	10,817,904	4,195,692	10,030,087	4,227,247	9,860,552	3,474,501	7,605,571	3,984,975	7,448,012	4,563,851
	Female /Male	% (95% CI)	11.47	11.97	12.09	11.98	12.16	11.75	13.60	11.89	13.84	12.37
0-<2	Male	% (95% CI)	0.0020 (0.0002-0.0073)	0.0007 (0.0000-0.0040)	0.0000 (0.0000-0.0039)	0.0000 (0.0000-0.0026)	0.0000 (0.0000-0.0040)	0.0000 (0.0000-0.0034)	0.0000 (0.0000-0.0052)	0.0000 (0.0000-0.0031)	0.0000 (0.0000-0.0054)	0.0000 (0.0000-0.0026)
		n	2	1	0	0	0	0	0	0	0	0
		N	99,163	140,756	95,576	140,505	92,999	109,715	70,272	118,477	68,871	140,386
	Female	% (95% CI)	0.0011 (0.0000-0.0060)	0.0000 (0.0000-0.0027)	0.0000 (0.0000-0.0041)	0.0000 (0.0000-0.0027)	0.0000 (0.0000-0.0041)	0.0000 (0.0000-0.0035)	0.0000 (0.0000-0.0054)	0.0000 (0.0000-0.0032)	0.0000 (0.0000-0.0056)	0.0007 (0.0000-0.0041)
		n	1	0	0	0	0	0	0	0	0	1
		N	93,619	134,582	91,052	134,755	88,969	104,661	67,704	113,803	66,232	134,959

Age	Sex	Statistic	2016		2017		2018		2019		2020	
			CCAE+MDCR	MDCD	CCAE+MDCR	MDCD	CCAE+MDCR	MDCD	CCAE+MDCR	MDCD	CCAE+MDCR	MDCD
	Female /Male	% (95% CI)	0.53	0.00								
2-<6	Male	% (95% CI)	0.0002 (0.0000-0.0013)	0.0002 (0.0000-0.0010)	0.0002 (0.0000-0.0014)	0.0002 (0.0000-0.0010)	0.0000 (0.0000-0.0009)	0.0000 (0.0000-0.0008)	0.0000 (0.0000-0.0012)	0.0004 (0.0000-0.0015)	0.0000 (0.0000-0.0012)	0.0004 (0.0000-0.0014)
		n	1	1	1	1	0	0	0	2	0	2
		N	428,385	560,386	412,335	557,638	411,664	448,411	315,396	493,015	307,596	530,797
	Female	% (95% CI)	0.0005 (0.0001-0.0018)	0.0002 (0.0000-0.0010)	0.0003 (0.0000-0.0014)	0.0000 (0.0000-0.0007)	0.0008 (0.0002-0.0022)	0.0000 (0.0000-0.0009)	0.0003 (0.0000-0.0018)	0.0000 (0.0000-0.0008)	0.0003 (0.0000-0.0019)	0.0000 (0.0000-0.0007)
		n	2	1	1	0	3	0	1	0	1	0
		N	408,886	532,771	393,409	529,559	393,031	424,850	301,890	466,473	293,333	502,730
	Female /Male	% (95% CI)	2.10	1.05	1.05	0.00				0.00		0.00
6-<12	Male	% (95% CI)	0.0013 (0.0006-0.0024)	0.0003 (0.0001-0.0010)	0.0011 (0.0005-0.0022)	0.0003 (0.0001-0.0010)	0.0004 (0.0001-0.0012)	0.0003 (0.0000-0.0010)	0.0020 (0.0010-0.0035)	0.0007 (0.0002-0.0015)	0.0011 (0.0004-0.0024)	0.0002 (0.0000-0.0009)
		n	10	3	8	3	3	2	11	5	6	2
		N	767,373	867,592	728,167	864,912	719,459	690,947	558,701	756,572	542,641	818,232
	Female	% (95% CI)	0.0020 (0.0011-0.0034)	0.0016 (0.0009-0.0027)	0.0019 (0.0010-0.0032)	0.0006 (0.0002-0.0014)	0.0016 (0.0008-0.0029)	0.0012 (0.0005-0.0025)	0.0021 (0.0010-0.0037)	0.0006 (0.0002-0.0015)	0.0012 (0.0004-0.0025)	0.0003 (0.0000-0.0009)
		n	15	13	13	5	11	8	11	4	6	2
		N	733,830	812,428	696,406	807,925	688,255	642,037	535,423	705,258	518,781	766,410
	Female /Male	% (95% CI)	1.57	4.63	1.70	1.78	3.83	4.30	1.04	0.86	1.05	1.07

Age	Sex	Statistic	2016		2017		2018		2019		2020	
			CCAE+MDCR	MDCD	CCAE+MDCR	MDCD	CCAE+MDCR	MDCD	CCAE+MDCR	MDCD	CCAE+MDCR	MDCD
0-<12	Male	% (95% CI)	0.0010 (0.0005-0.0017)	0.0003 (0.0001-0.0007)	0.0007 (0.0003-0.0014)	0.0003 (0.0001-0.0007)	0.0002 (0.0001-0.0007)	0.0002 (0.0000-0.0006)	0.0012 (0.0006-0.0021)	0.0005 (0.0002-0.0011)	0.0007 (0.0002-0.0014)	0.0003 (0.0001-0.0007)
		n	13	5	9	4	3	2	11	7	6	4
		N	1,294,921	1,568,734	1,236,078	1,563,055	1,224,122	1,249,073	944,369	1,368,064	919,108	1,489,415
	Female	% (95% CI)	0.0015 (0.0009-0.0023)	0.0009 (0.0005-0.0016)	0.0012 (0.0006-0.0020)	0.0003 (0.0001-0.0008)	0.0012 (0.0007-0.0020)	0.0007 (0.0003-0.0013)	0.0013 (0.0007-0.0023)	0.0003 (0.0001-0.0008)	0.0008 (0.0003-0.0016)	0.0002 (0.0000-0.0006)
		n	18	14	14	5	14	8	12	4	7	3
		N	1,236,335	1,479,781	1,180,867	1,472,239	1,170,255	1,171,548	905,017	1,285,534	878,346	1,404,099
	Female /Male	% (95% CI)	1.45	2.97	1.63	1.33	4.88	4.26	1.14	0.61	1.22	0.80
12-<18	Male	% (95% CI)	0.01 (0.01-0.01)	0.0044 (0.0030-0.0062)	0.01 (0.00-0.01)	0.0038 (0.0026-0.0055)	0.0046 (0.0033-0.0063)	0.0029 (0.0017-0.0045)	0.0045 (0.0030-0.0064)	0.0025 (0.0015-0.0040)	0.0040 (0.0026-0.0059)	0.0019 (0.0011-0.0031)
		n	60	33	47	29	39	18	30	18	26	15
		N	887,961	750,403	846,485	760,611	839,984	630,669	667,651	709,555	646,102	790,425
	Female	% (95% CI)	0.03 (0.02-0.03)	0.03 (0.03-0.03)	0.03 (0.02-0.03)	0.03 (0.02-0.03)	0.03 (0.03-0.04)	0.03 (0.02-0.03)	0.03 (0.02-0.03)	0.02 (0.02-0.02)	0.02 (0.02-0.03)	0.01 (0.01-0.02)
		n	240	204	229	200	255	151	186	130	138	104
		N	855,521	704,661	814,558	713,308	807,797	587,763	641,882	663,839	618,782	740,569
	Female /Male	% (95% CI)	4.15	6.58	5.06	7.35	6.80	9.00	6.45	7.72	5.54	7.40

Age	Sex	Statistic	2016		2017		2018		2019		2020	
			CCAE+MDCR	MDCD	CCAE+MDCR	MDCD	CCAE+MDCR	MDCD	CCAE+MDCR	MDCD	CCAE+MDCR	MDCD
0-<18	Male	% (95% CI)	0.0033 (0.0026-0.0042)	0.00 (0.00-0.00)	0.0027 (0.0020-0.0035)	0.00 (0.00-0.00)	0.0020 (0.0015-0.0028)	0.0011 (0.0006-0.0016)	0.0025 (0.0018-0.0035)	0.0012 (0.0008-0.0018)	0.0020 (0.0014-0.0029)	0.0008 (0.0005-0.0013)
		n	73	38	56	33	42	20	41	25	32	19
		N	2,182,882	2,319,137	2,082,563	2,323,666	2,064,106	1,879,742	1,612,020	2,077,619	1,565,210	2,279,840
	Female	% (95% CI)	0.01 (0.01-0.01)	0.01 (0.01-0.01)	0.01 (0.01-0.01)	0.01 (0.01-0.01)	0.01 (0.01-0.02)	0.01 (0.01-0.01)	0.01 (0.01-0.01)	0.01 (0.01-0.01)	0.01 (0.01-0.01)	0.005 (0.004-0.01)
		n	258	218	243	205	269	159	198	134	145	107
		N	2,091,856	2,184,442	1,995,425	2,185,547	1,978,052	1,759,311	1,546,899	1,949,373	1,497,128	2,144,668
	Female /Male	% (95% CI)	3.69	6.09	4.53	6.60	6.68	8.49	5.03	5.71	4.74	5.99
≥18	Male	% (95% CI)	0.09 (0.08-0.09)	0.22 (0.21-0.23)	0.08 (0.08-0.08)	0.21 (0.20-0.22)	0.07 (0.07-0.07)	0.22 (0.21-0.23)	0.06 (0.06-0.07)	0.20 (0.19-0.21)	0.06 (0.06-0.06)	0.15 (0.14-0.16)
		n	6,655	2,285	5,668	2,204	5,203	1,958	3,593	2,232	3,270	2,061
		N	7,778,203	1,027,354	7,264,590	1,066,075	7,224,647	893,854	5,676,856	1,125,843	5,568,393	1,365,872
	Female	% (95% CI)	0.96 (0.95-0.96)	1.72 (1.70-1.74)	0.92 (0.91-0.93)	1.63 (1.61-1.64)	0.86 (0.85-0.86)	1.69 (1.67-1.71)	0.85 (0.84-0.85)	1.63 (1.62-1.65)	0.80 (0.79-0.81)	1.33 (1.31-1.34)
		n	83,544	34,644	73,998	33,219	67,409	28,948	51,356	33,261	47,565	32,111
		N	8,726,048	2,011,250	8,034,662	2,041,700	7,882,500	1,715,190	6,058,672	2,035,602	5,950,884	2,419,183

Age	Sex	Statistic	2016		2017		2018		2019		2020	
			CCAE+MDCR	MDCD	CCAE+MDCR	MDCD	CCAE+MDCR	MDCD	CCAE+MDCR	MDCD	CCAE+MDCR	MDCD
	Female /Male	% (95% CI)	11.19	7.74	11.80	7.87	11.87	7.70	13.39	8.24	13.61	8.80
18-<40	Male	% (95% CI)	0.04 (0.04-0.04)	0.11 (0.10-0.12)	0.03 (0.03-0.04)	0.09 (0.09-0.10)	0.03 (0.03-0.03)	0.10 (0.09-0.11)	0.03 (0.02-0.03)	0.09 (0.08-0.10)	0.02 (0.02-0.03)	0.07 (0.06-0.07)
		n	1,114	657	939	605	898	519	596	588	547	561
		N	2,948,705	614,788	2,839,524	641,114	2,899,079	536,666	2,379,254	655,086	2,282,260	824,123
	Female	% (95% CI)	0.35 (0.35-0.36)	0.85 (0.84-0.87)	0.34 (0.33-0.34)	0.77 (0.76-0.79)	0.32 (0.31-0.33)	0.77 (0.76-0.79)	0.31 (0.31-0.32)	0.72 (0.70-0.73)	0.28 (0.28-0.29)	0.56 (0.55-0.57)
		n	11,072	11,650	10,181	10,645	9,708	8,847	7,535	9,411	6,549	8,938
		N	3,140,150	1,362,742	3,011,894	1,378,694	3,042,571	1,147,403	2,411,074	1,314,836	2,313,919	1,594,917
	Female /Male	% (95% CI)	9.33	8.00	10.22	8.18	10.30	7.97	12.48	7.97	11.81	8.23
40-<50	Male	% (95% CI)	0.09 (0.09-0.10)	0.46 (0.43-0.49)	0.09 (0.09-0.10)	0.42 (0.39-0.46)	0.08 (0.08-0.09)	0.45 (0.42-0.49)	0.07 (0.07-0.08)	0.37 (0.34-0.40)	0.06 (0.05-0.06)	0.28 (0.26-0.31)
		n	1,416	730	1,305	686	1,223	603	851	634	638	593
		N	1,495,617	158,865	1,440,946	162,431	1,454,380	133,424	1,160,165	173,093	1,093,123	210,508
	Female	% (95% CI)	1.10 (1.08-1.11)	3.44 (3.37-3.50)	1.08 (1.06-1.09)	3.23 (3.17-3.29)	1.03 (1.01-1.04)	3.29 (3.22-3.36)	1.02 (1.00-1.04)	3.05 (2.99-3.11)	0.92 (0.90-0.94)	2.51 (2.46-2.56)
		n	18,486	10,536	17,315	10,168	16,492	8,812	12,838	9,879	10,966	9,731
		N	1,686,532	306,477	1,610,569	314,647	1,606,715	267,892	1,259,555	323,991	1,189,714	387,113

Age	Sex	Statistic	2016		2017		2018		2019		2020	
			CCAE+MDCR	MDCD	CCAE+MDCR	MDCD	CCAE+MDCR	MDCD	CCAE+MDCR	MDCD	CCAE+MDCR	MDCD
	Female/Male	% (95% CI)	11.58	7.48	11.87	7.65	12.21	7.28	13.90	8.32	15.79	8.92
50-<60	Male	% (95% CI)	0.12 (0.12-0.13)	0.40 (0.37-0.43)	0.11 (0.11-0.12)	0.38 (0.35-0.41)	0.11 (0.10-0.11)	0.41 (0.38-0.45)	0.10 (0.09-0.10)	0.39 (0.36-0.42)	0.09 (0.09-0.10)	0.32 (0.29-0.34)
		n	2,138	695	1,870	674	1,776	610	1,261	718	1,108	648
		N	1,733,395	175,141	1,641,470	177,094	1,646,485	147,371	1,281,760	183,685	1,209,493	203,896
	Female	% (95% CI)	1.45 (1.43-1.46)	4.02 (3.94-4.10)	1.41 (1.39-1.43)	3.92 (3.84-3.99)	1.34 (1.32-1.36)	4.12 (4.03-4.21)	1.33 (1.31-1.34)	3.96 (3.88-4.04)	1.23 (1.21-1.25)	3.42 (3.36-3.49)
		n	28,614	9,526	26,065	9,387	24,345	8,393	18,579	9,817	16,197	9,401
		N	1,979,638	236,759	1,847,821	239,740	1,816,189	203,673	1,401,479	248,036	1,317,871	274,498
	Female/Male	% (95% CI)	11.72	10.14	12.38	10.29	12.43	9.96	13.47	10.13	13.42	10.78
60-<70	Male	% (95% CI)	0.14 (0.13-0.15)	0.27 (0.24-0.31)	0.12 (0.12-0.13)	0.29 (0.25-0.33)	0.11 (0.11-0.12)	0.30 (0.27-0.35)	0.11 (0.10-0.11)	0.27 (0.24-0.31)	0.10 (0.09-0.11)	0.21 (0.18-0.24)
		n	1,474	195	1,178	229	1,038	215	727	266	693	233
		N	1,069,282	71,665	956,188	79,065	923,785	70,646	690,570	98,315	698,287	111,001
	Female	% (95% CI)	1.51 (1.48-1.53)	3.17 (3.06-3.29)	1.44 (1.42-1.46)	3.03 (2.92-3.14)	1.30 (1.27-1.32)	3.30 (3.18-3.42)	1.29 (1.27-1.32)	3.23 (3.13-3.34)	1.24 (1.21-1.26)	2.82 (2.73-2.91)
		n	18,535	2,838	15,511	2,902	13,286	2,794	9,919	3,765	9,629	3,646
		N	1,230,667	89,414	1,076,219	95,852	1,024,959	84,769	767,460	116,483	776,863	129,173
	Female/Male	% (95% CI)	10.93	11.66	11.70	10.45	11.54	10.83	12.28	11.95	12.49	13.45

Age	Sex	Statistic	2016		2017		2018		2019		2020	
			**CCAE+MDCR**	**MDCD**	**CCAE+MDCR**	**MDCD**	**CCAE+MDCR**	**MDCD**	**CCAE+MDCR**	**MDCD**	**CCAE+MDCR**	**MDCD**
70-<80	Male	% (95% CI)	0.11 (0.10-0.12)	0.13 (0.05-0.29)	0.11 (0.10-0.12)	0.22 (0.10-0.40)	0.10 (0.09-0.12)	0.26 (0.13-0.46)	0.11 (0.09-0.13)	0.20 (0.13-0.31)	0.11 (0.09-0.13)	0.20 (0.12-0.30)
		n	366	6	271	10	202	11	118	21	208	22
		N	333,765	4,506	249,760	4,614	197,632	4,241	109,623	10,289	190,250	11,062
	Female	% (95% CI)	1.32 (1.28-1.35)	0.95 (0.76-1.17)	1.33 (1.28-1.37)	1.20 (0.99-1.45)	1.15 (1.11-1.19)	1.19 (0.96-1.44)	1.40 (1.34-1.46)	1.69 (1.51-1.88)	1.47 (1.42-1.52)	1.60 (1.43-1.78)
		n	5,105	87	3,769	108	2,731	97	1,917	322	3,204	323
		N	387,660	9,191	284,192	8,981	237,957	8,185	137,233	19,097	217,811	20,250
	Female /Male	% (95% CI)	12.01	7.11	12.22	5.55	11.23	4.57	12.98	8.26	13.45	8.02
≥80	Male	% (95% CI)	0.07 (0.06-0.09)	0.08 (0.01-0.30)	0.08 (0.06-0.09)	0.00 (0.00-0.21)	0.06 (0.05-0.08)	0.00 (0.00-0.24)	0.07 (0.05-0.10)	0.09 (0.03-0.22)	0.08 (0.06-0.10)	0.08 (0.02-0.19)
		n	147	2	105	0	66	0	40	5	76	4
		N	197,439	2,389	136,702	1,757	103,286	1,506	55,484	5,375	94,980	5,282
	Female	% (95% CI)	0.57 (0.55-0.60)	0.10 (0.04-0.22)	0.57 (0.54-0.60)	0.24 (0.11-0.45)	0.55 (0.51-0.59)	0.15 (0.05-0.36)	0.69 (0.64-0.75)	0.51 (0.39-0.65)	0.76 (0.71-0.80)	0.54 (0.43-0.68)
		n	1,732	7	1,157	9	847	5	568	67	1,020	72
		N	301,401	6,667	203,967	3,786	154,109	3,268	81,871	13,159	134,706	13,232
	Female /Male	% (95% CI)	7.72	1.25	7.39		8.60		9.62	5.47	9.46	7.19

CCAE=Commercial Claims and Encounters database; MDCR= Medicare Supplemental and Coordination of Benefits Database; MDCD=Medicaid Database

Supplementary Table 4: Annual prevalence of diagnosed fibromyalgia (≥2 diagnosis codes) by age groups and geographical regions in Commercial + Medicare databases

		2016			2017			2018			2019			2020		
**Regions**		**0-<18 years**	**≥18 years**	**All ages**	**0-<18 years**	**≥18 years**	**All ages**	**0-<18 years**	**≥18 years**	**All ages**	**0-<18 years**	**≥18 years**	**All ages**	**0-<18 years**	**≥18 years**	**All ages**
Northeast Region	% (95% CI)	0.01 (0.01-0.01)	0.43 (0.42-0.43)	0.35 (0.34-0.35)	0.01 (0.01-0.01)	0.42 (0.41-0.43)	0.34 (0.33-0.35)	0.01 (0.01-0.01)	0.38 (0.37-0.39)	0.30 (0.30-0.31)	0.01 (0.00-0.01)	0.32 (0.31-0.33)	0.26 (0.25-0.27)	0.00 (0.00-0.01)	0.29 (0.29-0.30)	0.24 (0.23-0.24)
North Central Region	% (95% CI)	0.01 (0.00-0.01)	0.62 (0.61-0.63)	0.50 (0.49-0.50)	0.01 (0.00-0.01)	0.61 (0.60-0.61)	0.48 (0.47-0.48)	0.01 (0.00-0.01)	0.56 (0.55-0.56)	0.44 (0.43-0.44)	0.01 (0.00-0.01)	0.57 (0.56-0.57)	0.45 (0.44-0.45)	0.01 (0.00-0.01)	0.55 (0.54-0.56)	0.44 (0.43-0.45)
South Region	% (95% CI)	0.01 (0.01-0.01)	0.59 (0.59-0.60)	0.47 (0.47-0.48)	0.01 (0.01-0.01)	0.55 (0.55-0.56)	0.44 (0.43-0.44)	0.01 (0.01-0.01)	0.52 (0.51-0.53)	0.41 (0.41-0.42)	0.01 (0.01-0.01)	0.50 (0.49-0.51)	0.40 (0.39-0.40)	0.01 (0.00-0.01)	0.47 (0.46-0.48)	0.37 (0.37-0.38)
West Region	% (95% CI)	0.01 (0.01-0.01)	0.45 (0.44-0.46)	0.35 (0.34-0.35)	0.01 (0.01-0.01)	0.42 (0.41-0.43)	0.33 (0.32-0.34)	0.01 (0.01-0.01)	0.39 (0.39-0.40)	0.31 (0.30-0.32)	0.01 (0.01-0.01)	0.37 (0.36-0.38)	0.29 (0.28-0.29)	0.01 (0.01-0.01)	0.33 (0.32-0.34)	0.26 (0.25-0.26)
Unknown Region	% (95% CI)	0.00 (0.00-0.02)	0.47 (0.41-0.54)	0.32 (0.28-0.37)	0.00 (0.00-0.02)	0.49 (0.42-0.57)	0.33 (0.28-0.38)	0.00 (0.00-0.01)	0.38 (0.33-0.43)	0.26 (0.22-0.30)	0.00 (0.00-0.02)	0.35 (0.30-0.40)	0.23 (0.20-0.27)	0.01 (0.00-0.05)	0.22 (0.18-0.27)	0.17 (0.14-0.21)

CCAE=Commercial Claims and Encounters database; MDCR= Medicare Supplemental and Coordination of Benefits Database; MDCD=Medicaid Database

Supplementary Table 5: Annual prevalence of diagnosed fibromyalgia (≥2 diagnosis codes) by age groups and race/ethnicity in the Medicaid database

Race/Ethnicity	Statistic	2016		2017		2018		2019		2020	
		0-<18 years	≥18 years	0-<18 years	≥18 years	0-<18 years	≥18 years	0-<18 years	≥18 years	0-<18 years	≥18 years
Missing	% (95% CI)	0.01 (0.01-0.01)		0.005 (0.003-0.01)	1.24 (1.21-1.28)	0.01 (0.00-0.01)	1.54 (1.49-1.60)	0.01 (0.00-0.01)	1.45 (1.41-1.50)	0.002 (0.001-0.005)	1.18 (1.14-1.22)
	n			24	4729	14	3280	20	3531	9	3,076
	N			503,206	380,842	256,850	212,536	293,180	242,905	363,176	260,748
White	% (95% CI)	0.003 (0.002-0.004)	1.68 (1.66-1.70)	0.01 (0.01-0.01)	1.57 (1.55-1.59)	0.01 (0.01-0.01)	1.63 (1.61-1.65)	0.01 (0.00-0.01)	1.50 (1.48-1.52)	0.005 (0.004-0.01)	1.22 (1.20-1.24)
	n	162	25,037	163	23,683	137	21600	103	22581	94	22,270
	N	2,062,526	1,492,863	2,019,501	1,510,102	1,722,962	1,325,858	1,733,216	1,506,126	1,881,321	1,825,088
Black	% (95% CI)	0.003 (0.001-0.005)	0.60 (0.58-0.61)	0.003(0.002-0.004)	0.55 (0.54-0.57)	0.002 (0.001-0.003)	0.57 (0.56-0.59)	0.002 (0.001-0.003)	0.56 (0.54-0.57)	0.001 (0.001-0.002)	0.44 (0.43-0.45)
	n	48	6043	39	5,769	21	5282	23	5383	16	5165
	N	1,469,794	1,010,222	1,451,080	1,043,220	1,226,642	923,301	1,244,990	966,034	1,383,986	1,175,173
Hispanic	% (95% CI)	0.01 (0.00-0.01)	0.86 (0.80-0.92)	0.002 (0.001-0.004)	0.82 (0.76-0.87)	0.001 (0.000-0.003)	0.39 (0.34-0.44)	0.002 (0.001-0.004)	1.00 (0.96-1.05)	0.001 (0.000-0.002)	0.79 (0.76-0.83)
	n	11	740	7	783	2	252	10	2246	5	2247
	N	394,860	86,297	382,616	95,984	281,553	65,287	491,031	223,514	519,068	283,027
Others	% (95% CI)	0.01 (0.01-0.01)	1.14 (1.11-1.17)	0.003 (0.001-0.01)	0.59 (0.54-0.65)	0.003 (0.001-0.01)	0.60 (0.55-0.65)	0.001 (0.00-0.003)	0.79 (0.75-0.82)	0.001 (0.000-0.003)	0.59 (0.56-0.62)

Race/Ethnicity	Statistic	2016		2017		2018		2019		2020	
		0-<18 years	≥18 years	0-<18 years	≥18 years	0-<18 years	≥18 years	0-<18 years	≥18 years	0-<18 years	≥18 years
	n	35	5109	5	459	5	492	3	1752	2	1414
	N	576,574	449,222	152,965	77,627	151,152	82,062	264,629	222,866	276,957	241,019

Supplementary Table 6: Annual prevalence of diagnosed fibromyalgia (≥1 diagnosis code) by age groups

		2016		2017		2018		2019		2020	
	**Statistics**	**CCAE+MDCR**	**MDCD**	**CCAE+MDCR**	**MDCD**	**CCAE+MDCR**	**MDCD**	**CCAE+MDCR**	**MDCD**	**CCAE+MDCR**	**MDCD**
All ages	% (95% CI)	0.64 (0.63-0.64)	0.66 (0.65-0.67)	0.60 (0.60-0.60)	0.63 (0.63-0.64)	0.56 (0.56-0.57)	0.66 (0.66-0.67)	0.54 (0.54-0.54)	0.67 (0.67-0.68)	0.51 (0.51-0.51)	0.57 (0.56-0.58)
	n	131,948	49,765	2	48,156	107,554	41,493	80,291	48,373	74,207	46,782
	N	20,778,989	7,542,183	186,628	7,616,988	19,149,305	6,248,097	14,894,447	7,188,437	14,581,615	8,209,563
0-<12	% (95% CI)	0.00 (0.00-0.00)	0.00 (0.00-0.00)	0.00 (0.00-0.00)	0.00 (0.00-0.00)	0.00 (0.00-0.00)	0.00 (0.00-0.00)	0.00 (0.00-0.00)	0.00 (0.00-0.00)	0.00 (0.00-0.00)	0.00 (0.00-0.00)
	n	93	101	11	47	71	38	38	25	26	28
	N	2,531,256	3,048,515	805,744	3,035,294	2,394,377	2,420,621	1,849,386	2,653,598	1,797,454	2,893,514
12-<18		0.03 (0.03-0.04)	0.03 (0.03-0.03)	0.03 (0.03-0.03)	0.03 (0.02-0.03)	0.03 (0.03-0.03)	0.02 (0.02-0.03)	0.03 (0.02-0.03)	0.02 (0.02-0.02)	0.02 (0.02-0.02)	0.01 (0.01-0.01)
		584	456	54	423	479	276	346	282	269	195
		1,743,482	1,455,064	1,424,573	4,509,213	1,647,781	1,218,432	1,309,533	1,373,394	1,264,884	1,530,994
0-<18		0.02 (0.01-0.02)	0.01 (0.01-0.01)	0.01 (0.01-0.01)	0.01 (0.01-0.01)	0.01 (0.01-0.01)	0.01 (0.01-0.01)	0.01 (0.01-0.01)	0.01 (0.01-0.01)	0.01 (0.01-0.01)	0.01 (0.00-0.01)
		677	557	475	4,509,213	550	314	384	307	295	223
		4,274,738	4,503,579	1,661,043	3,107,775	4,042,158	3,639,053	3,158,919	4,026,992	3,062,338	4,424,508
>=18		0.80 (0.79-0.80)	1.62 (1.61-1.63)	0.76 (0.75-0.76)	1.54 (1.52-1.55)	0.71 (0.70-0.71)	1.58 (1.56-1.59)	0.68 (0.68-0.69)	1.52 (1.51-1.53)	0.64 (0.64-0.65)	1.23 (1.22-1.24)

		2016		2017		2018		2019		2020	
	**Statistics**	**CCAE+MDCR**	**MDCD**	**CCAE+MDCR**	**MDCD**	**CCAE+MDCR**	**MDCD**	**CCAE+MDCR**	**MDCD**	**CCAE+MDCR**	**MDCD**
		131,271	49,208	115,748	47,733	4,042,158	41,179	79,907	0	73,912	46,559
		16,504,251	3,038,604	15,299,252	3,107,775	15,107,147	2,609,044	11,735,528	232,280	11,519,277	3,785,055
0-<2		0.00 (0.00-0.01)	0.00 (0.00-0.00)	0.00 (0.00-0.00)	0.00 (0.00-0.00)	0.00 (0.00-0.00)	0.00 (0.00-0.00)	0.00 (0.00-0.00)	0.00 (0.00-0.00)	0.00 (0.00-0.00)	0.00 (0.00-0.00)
		7	6	2	1	0	1	1	0	0	4
		192,782	275,338	186,628	275,260	181,968	214,376	137,976	232,280	135,103	275,345
2-<6		0.00 (0.00-0.00)	0.00 (0.00-0.00)	0.00 (0.00-0.00)	0.00 (0.00-0.00)	0.00 (0.00-0.00)	0.00 (0.00-0.00)	0.00 (0.00-0.00)	0.00 (0.00-0.00)	0.00 (0.00-0.00)	0.00 (0.00-0.00)
		15	15	11	8	16	2	2	4	5	8
		837,271	1,093,157	805,744	1,087,197	804,695	873,261	617,286	959,488	600,929	1,033,527
6-<12		0.00 (0.00-0.01)	0.00 (0.00-0.01)	0.00 (0.00-0.00)	0.00 (0.00-0.00)	0.00 (0.00-0.01)	0.00 (0.00-0.00)	0.00 (0.00-0.00)	0.00 (0.00-0.00)	0.00 (0.00-0.00)	0.00 (0.00-0.00)
		71	80	54	38	55	35	35	21	21	16
		1,501,203	1,680,020	1,424,573	1,672,837	1,407,714	1,332,984	1,094,124	1,461,830	1,061,422	1,584,642
12-<18		0.03 (0.03-0.04)	0.03 (0.03-0.03)	0.03 (0.03-0.03)	0.03 (0.02-0.03)	0.03 (0.03-0.03)	0.02 (0.02-0.03)	0.03 (0.02-0.03)	0.02 (0.02-0.02)	0.02 (0.02-0.02)	0.01 (0.01-0.01)
		584	456	475	376	479	276	346	282	269	195
		1,743,482	1,455,064	1,661,043	1,473,919	1,647,781	1,218,432	1,309,533	1,373,394	1,264,884	1,530,994

		2016		2017		2018		2019		2020	
	**Statistics**	**CCAE+MDCR**	**MDCD**	**CCAE+MDCR**	**MDCD**	**CCAE+MDCR**	**MDCD**	**CCAE+MDCR**	**MDCD**	**CCAE+MDCR**	**MDCD**
18-<40		0.30 (0.30-0.30)	0.84 (0.83-0.85)	0.28 (0.28-0.29)	0.76 (0.75-0.77)	0.27 (0.27-0.27)	0.75 (0.74-0.77)	0.25 (0.25-0.26)	0.70 (0.68-0.71)	0.23 (0.23-0.23)	0.54 (0.54-0.55)
		18,262	16,645	16,450	15,386	16,012	12,677	12,123	13,713	10,577	13,183
		6,088,855	1,977,530	5,851,418	2,019,808	5,941,650	1,684,069	4,790,328	1,969,922	4,596,179	2,419,040
40-<50		0.89 (0.88-0.90)	3.15 (3.10-3.20)	0.86 (0.85-0.87)	3.00 (2.95-3.05)	0.83 (0.82-0.84)	3.07 (3.01-3.12)	0.80 (0.79-0.81)	2.81 (2.76-2.85)	0.72 (0.71-0.74)	2.28 (2.25-2.32)
		28,180	14,637	26,286	14,301	25,441	12,311	19,446	13,944	16,527	13,645
		3,182,149	465,342	3,051,515	477,078	3,061,095	401,316	2,419,720	497,084	2,282,837	597,621
50-<60		1.17 (1.16-1.18)	3.32 (3.27-3.38)	1.14 (1.13-1.15)	3.25 (3.20-3.30)	1.09 (1.08-1.10)	3.40 (3.34-3.46)	1.06 (1.05-1.07)	3.29 (3.23-3.34)	0.98 (0.97-0.99)	2.88 (2.83-2.93)
		43,537	13,678	39,768	13,545	37,801	11,923	28,437	14,193	24,775	13,768
		3,713,033	411,900	3,489,291	416,834	3,462,674	351,044	2,683,239	431,721	2,527,364	478,394
60-<70		1.29 (1.27-1.30)	2.54 (2.47-2.62)	1.22 (1.20-1.23)	2.46 (2.39-2.54)	1.11 (1.10-1.13)	2.64 (2.56-2.72)	1.09 (1.07-1.10)	2.62 (2.55-2.68)	1.04 (1.03-1.06)	2.25 (2.19-2.31)
		29,600	4,095	24,760	4,307	21,659	4,099	15,845	5,620	15,385	5,393
		2,299,949	161,079	2,032,407	174,917	1,948,744	155,415	1,458,030	214,798	1,475,150	240,174
70-<80		1.19 (1.17-1.22)	1.00 (0.84-1.18)	1.20 (1.17-1.23)	1.29 (1.10-1.49)	1.05 (1.02-1.08)	1.28 (1.09-1.49)	1.25 (1.21-1.30)	1.66 (1.52-1.81)	1.23 (1.20-1.27)	1.49 (1.36-1.63)
		8,594	137	6,409	175	4,592	159	3,088	488	5,033	467
		721,425	13,697	533,952	13,595	435,589	12,426	246,856	29,386	408,061	31,312

		2016		2017		2018		2019		2020	
	**Statistics**	**CCAE+MDCR**	**MDCD**	**CCAE+MDCR**	**MDCD**	**CCAE+MDCR**	**MDCD**	**CCAE+MDCR**	**MDCD**	**CCAE+MDCR**	**MDCD**
>=80		0.62 (0.60-0.64)	0.18 (0.10-0.29)	0.61 (0.58-0.64)	0.34 (0.21-0.53)	0.58 (0.55-0.61)	0.21 (0.10-0.38)	0.70 (0.66-0.75)	0.58 (0.48-0.70)	0.70 (0.67-0.74)	0.56 (0.45-0.67)
		3,098	16	2,075	19	1,499	10	968	108	1,615	103
		498,840	9,056	340,669	5,543	257,395	4,774	137,355	18,534	229,686	18,514

CCAE=Commercial Claims and Encounters database; MDCR= Medicare Supplemental and Coordination of Benefits Database; MDCD=Medicaid Database

Supplementary Table 7: Annual prevalence of diagnosed fibromyalgia (≥1 diagnosis code) by age groups and sex

Age	Sex	Statistic	2016		2017		2018		2019		2020	
			CCAE+MDCR	MDCD	CCAE+MDCR	MDCD	CCAE+MDCR	MDCD	CCAE+MDCR	MDCD	CCAE+MDCR	MDCD
All ages	Male	% (95% CI)	0.12 (0.11-0.12)	0.11 (0.10-0.11)	0.10 (0.10-0.11)	0.10 (0.10-0.10)	0.10 (0.10-0.10)	0.10 (0.10-0.11)	0.08 (0.08-0.09)	0.11 (0.10-0.11)	0.08 (0.08-0.08)	0.09 (0.08-0.09)
		n	11,655	3,609	9,627	3,339	9,027	2,905	6,179	3,365	5,546	3,123
		N	9,961,085	3,346,491	9,347,153	3,389,741	9,288,753	2,773,596	7,288,876	3,203,462	7,133,603	3,645,712
	Female	% (95% CI)	1.11 (1.11-1.12)	1.10 (1.09-1.11)	1.06 (1.06-1.07)	1.06 (1.05-1.07)	1.00 (0.99-1.01)	1.11 (1.10-1.12)	0.97 (0.97-0.98)	1.13 (1.12-1.14)	0.92 (0.92-0.93)	0.96 (0.95-0.97)
		n	120,293	46,156	106,663	44,817	98,527	38,588	74,112	45,008	68,661	43,659
		N	10,817,904	4,195,692	10,030,087	4,227,247	9,860,552	3,474,501	7,605,571	3,984,975	7,448,012	4,563,851
	Female /Male	% (95% CI)	9.5	10.2	10.33	10.76	10.28	10.6	11.49	10.75	11.86	11.17
0-<2	Male	% (95% CI)	0.00 (0.00-0.01)	0.00 (0.00-0.01)	0.00 (0.00-0.01)	0.00 (0.00-0.00)	0.00 (0.00-0.00)	0.00 (0.00-0.00)	0.00 (0.00-0.01)	0.00 (0.00-0.00)	0.00 (0.00-0.01)	0.00 (0.00-0.01)
		n	4	3	1	1	0	0	0	0	0	2
		N	99,163	140,756	95,576	140,505	92,999	109,715	70,272	118,477	68,871	140,386
	Female	% (95% CI)	0.00 (0.00-0.01)	0.00 (0.00-0.01)	0.00 (0.00-0.01)	0.00 (0.00-0.00)	0.00 (0.00-0.00)	0.00 (0.00-0.01)	0.00 (0.00-0.01)	0.00 (0.00-0.00)	0.00 (0.00-0.01)	0.00 (0.00-0.01)
		n	3	3	1	0	0	1	1	0	0	2
		N	93,619	134,582	91,052	134,755	88,969	104,661	67,704	113,803	66,232	134,959

Age	Sex	Statistic	2016		2017		2018		2019		2020	
			CCAE+MDCR	MDCD	CCAE+MDCR	MDCD	CCAE+MDCR	MDCD	CCAE+MDCR	MDCD	CCAE+MDCR	MDCD
	Female /Male	% (95% CI)	0.79	1.05	1.05	0						1.04
2- <6	Male	% (95% CI)	0.00 (0.00-0.00)	0.00 (0.00-0.00)	0.00 (0.00-0.00)	0.00 (0.00-0.00)	0.00 (0.00-0.00)	0.00 (0.00-0.00)	0.00 (0.00-0.00)	0.00 (0.00-0.00)	0.00 (0.00-0.00)	0.00 (0.00-0.00)
		n	7	5	5	4	7	0	0	3	3	2
		N	428,385	560,386	412,335	557,638	411,664	448,411	315,396	493,015	307,596	530,797
	Female	% (95% CI)	0.00 (0.00-0.00)	0.00 (0.00-0.00)	0.00 (0.00-0.00)	0.00 (0.00-0.00)	0.00 (0.00-0.00)	0.00 (0.00-0.00)	0.00 (0.00-0.00)	0.00 (0.00-0.00)	0.00 (0.00-0.00)	0.00 (0.00-0.00)
		n	8	10	6	4	9	2	2	1	2	6
		N	408,886	532,771	393,409	529,559	393,031	424,850	301,890	466,473	293,333	502,730
	Female /Male	% (95% CI)	1.2	2.1	1.26	1.05	1.35			0.35	0.7	3.17
6- <12	Male	% (95% CI)	0.00 (0.00-0.01)	0.00 (0.00-0.01)	0.00 (0.00-0.00)	0.00 (0.00-0.00)	0.00 (0.00-0.00)	0.00 (0.00-0.00)	0.00 (0.00-0.00)	0.00 (0.00-0.00)	0.00 (0.00-0.00)	0.00 (0.00-0.00)
		n	33	31	19	15	24	12	16	11	11	9
		N	767,373	867,592	728,167	864,912	719,459	690,947	558,701	756,572	542,641	818,232
	Female	% (95% CI)	0.01 (0.00-0.01)	0.01 (0.00-0.01)	0.01 (0.00-0.01)	0.00 (0.00-0.00)	0.00 (0.00-0.01)	0.00 (0.00-0.01)	0.00 (0.00-0.01)	0.00 (0.00-0.00)	0.00 (0.00-0.00)	0.00 (0.00-0.00)
		n	38	49	35	23	31	23	19	10	10	7
		N	733,830	812,428	696,406	807,925	688,255	642,037	535,423	705,258	518,781	766,410
	Female /Male	% (95% CI)	1.2	1.69	1.93	1.64	1.35	2.06	1.24	0.98	0.95	0.83

Age	Sex	Statistic	2016		2017		2018		2019		2020	
			CCAE+MDCR	MDCD	CCAE+MDCR	MDCD	CCAE+MDCR	MDCD	CCAE+MDCR	MDCD	CCAE+MDCR	MDCD
0-<12	Male	% (95% CI)	0.00 (0.00-0.00)	0.00 (0.00-0.00)	0.00 (0.00-0.00)	0.00 (0.00-0.00)	0.00 (0.00-0.00)	0.00 (0.00-0.00)	0.00 (0.00-0.00)	0.00 (0.00-0.00)	0.00 (0.00-0.00)	0.00 (0.00-0.00)
		n	44	39	25	20	31	12	16	14	14	13
		N	1,294,921	1,568,734	1,236,078	1,563,055	1,224,122	1,249,073	944,369	1,368,064	919,108	1,489,415
	Female	% (95% CI)	0.00 (0.00-0.01)	0.00 (0.00-0.01)	0.00 (0.00-0.00)	0.00 (0.00-0.00)	0.00 (0.00-0.00)	0.00 (0.00-0.00)	0.00 (0.00-0.00)	0.00 (0.00-0.00)	0.00 (0.00-0.00)	0.00 (0.00-0.00)
		n	49	62	42	27	40	26	22	11	12	15
		N	1,236,335	1,479,781	1,180,867	1,472,239	1,170,255	1,171,548	905,017	1,285,534	878,346	1,404,099
	Female /Male	% (95% CI)	1.17	1.69	1.76	1.43	1.35	2.31	1.43	0.84	0.9	1.22
12-<18	Male	% (95% CI)	0.01 (0.01-0.02)	0.01 (0.01-0.02)	0.01 (0.01-0.01)	0.01 (0.01-0.01)	0.01 (0.01-0.01)	0.01 (0.00-0.01)	0.01 (0.01-0.01)	0.01 (0.00-0.01)	0.01 (0.01-0.01)	0.00 (0.00-0.00)
		n	128	105	91	54	79	32	53	40	50	26
		N	887,961	750,403	846,485	760,611	839,984	630,669	667,651	709,555	646,102	790,425
	Female	% (95% CI)	0.05 (0.05-0.06)	0.05 (0.04-0.06)	0.05 (0.04-0.05)	0.05 (0.04-0.05)	0.05 (0.04-0.05)	0.04 (0.04-0.05)	0.05 (0.04-0.05)	0.04 (0.03-0.04)	0.04 (0.03-0.04)	0.02 (0.02-0.03)
		n	456	351	384	322	400	244	293	242	219	169
		N	855,521	704,661	814,558	713,308	807,797	587,763	641,882	663,839	618,782	740,569
	Female /Male	% (95% CI)	3.7	3.56	4.39	6.36	5.27	8.18	5.75	6.47	4.57	6.94

Age	Sex	Statistic	2016		2017		2018		2019		2020	
			CCAE+MDCR	MDCD	CCAE+MDCR	MDCD	CCAE+MDCR	MDCD	CCAE+MDCR	MDCD	CCAE+MDCR	MDCD
0-<18	Male	% (95% CI)	0.01 (0.01-0.01)	0.01 (0.01-0.01)	0.01 (0.00-0.01)	0.00 (0.00-0.00)	0.01 (0.00-0.01)	0.00 (0.00-0.00)	0.00 (0.00-0.01)	0.00 (0.00-0.00)	0.00 (0.00-0.01)	0.00 (0.00-0.00)
		n	172	144	116	74	110	44	69	54	64	39
		N	2,182,882	2,319,137	2,082,563	2,323,666	2,064,106	1,879,742	1,612,020	2,077,619	1,565,210	2,279,840
	Female	% (95% CI)	0.02 (0.02-0.03)	0.02 (0.02-0.02)	0.02 (0.02-0.02)	0.02 (0.01-0.02)	0.02 (0.02-0.02)	0.02 (0.01-0.02)	0.02 (0.02-0.02)	0.01 (0.01-0.01)	0.02 (0.01-0.02)	0.01 (0.01-0.01)
		n	505	413	426	349	440	270	315	253	231	184
		N	2,091,856	2,184,442	1,995,425	2,185,547	1,978,052	1,759,311	1,546,899	1,949,373	1,497,128	2,144,668
	Female /Male	% (95% CI)	3.06	3.04	3.83	5.01	4.17	6.56	4.76	4.99	3.77	5.02
≥18	Male	% (95% CI)	0.15 (0.14-0.15)	0.34 (0.33-0.35)	0.13 (0.13-0.13)	0.31 (0.30-0.32)	0.12 (0.12-0.13)	0.32 (0.31-0.33)	0.11 (0.10-0.11)	0.29 (0.28-0.30)	0.10 (0.10-0.10)	0.23 (0.22-0.23)
		n	11,483	3,465	9,511	3,265	8,917	2,861	6,110	3,311	5,482	3,084
		N	7,778,203	1,027,354	7,264,590	1,066,075	7,224,647	893,854	5,676,856	1,125,843	5,568,393	1,365,872
	Female	% (95% CI)	1.37 (1.37-1.38)	2.27 (2.25-2.30)	1.32 (1.31-1.33)	2.18 (2.16-2.20)	1.24 (1.24-1.25)	2.23 (2.21-2.26)	1.22 (1.21-1.23)	2.20 (2.18-2.22)	1.15 (1.14-1.16)	1.80 (1.78-1.81)
		n	119,788	45,743	106,237	44,468	98,087	38,318	73,797	44,755	68,430	43,475
		N	8,726,048	2,011,250	8,034,662	2,041,700	7,882,500	1,715,190	6,058,672	2,035,602	5,950,884	2,419,183

Age	Sex	Statistic	2016		2017		2018		2019		2020	
			CCAE+MDCR	MDCD	CCAE+MDCR	MDCD	CCAE+MDCR	MDCD	CCAE+MDCR	MDCD	CCAE+MDCR	MDCD
	Female /Male	% (95% CI)	9.3	6.74	10.1	7.11	10.08	6.98	11.32	7.48	11.68	7.96
18-<40	Male	% (95% CI)	0.07 (0.07-0.07)	0.16 (0.15-0.17)	0.06 (0.05-0.06)	0.14 (0.13-0.15)	0.06 (0.05-0.06)	0.14 (0.13-0.15)	0.05 (0.04-0.05)	0.13 (0.12-0.14)	0.04 (0.04-0.04)	0.10 (0.10-0.11)
		n	2,030	993	1,621	901	1,605	774	1,080	875	962	842
		N	2,948,705	614,788	2,839,524	641,114	2,899,079	536,666	2,379,254	655,086	2,282,260	824,123
	Female	% (95% CI)	0.52 (0.51-0.52)	1.15 (1.13-1.17)	0.49 (0.48-0.50)	1.05 (1.03-1.07)	0.47 (0.47-0.48)	1.04 (1.02-1.06)	0.46 (0.45-0.47)	0.98 (0.96-0.99)	0.42 (0.41-0.42)	0.77 (0.76-0.79)
		n	16,232	15,652	14,829	14,485	14,407	11,903	11,043	12,838	9,615	12,341
		N	3,140,150	1,362,742	3,011,894	1,378,694	3,042,571	1,147,403	2,411,074	1,314,836	2,313,919	1,594,917
	Female /Male	% (95% CI)	7.51	7.11	8.62	7.48	8.55	7.19	10.09	7.31	9.86	7.57
40-<50	Male	% (95% CI)	0.16 (0.15-0.17)	0.67 (0.63-0.71)	0.15 (0.14-0.16)	0.59 (0.55-0.63)	0.14 (0.13-0.15)	0.64 (0.60-0.69)	0.12 (0.12-0.13)	0.51 (0.48-0.55)	0.10 (0.09-0.10)	0.39 (0.37-0.42)
		n	2,398	1,068	2,151	961	2,028	860	1,417	884	1,077	829
		N	1,495,617	158,865	1,440,946	162,431	1,454,380	133,424	1,160,165	173,093	1,093,123	210,508
	Female	% (95% CI)	1.53 (1.51-1.55)	4.43 (4.35-4.50)	1.50 (1.48-1.52)	4.24 (4.17-4.31)	1.46 (1.44-1.48)	4.27 (4.20-4.35)	1.43 (1.41-1.45)	4.03 (3.96-4.10)	1.30 (1.28-1.32)	3.31 (3.25-3.37)
		n	25,782	13,569	24,135	13,340	23,413	11,451	18,029	13,060	15,450	12,816
		N	1,686,532	306,477	1,610,569	314,647	1,606,715	267,892	1,259,555	323,991	1,189,714	387,113

Age	Sex	Statistic	2016		2017		2018		2019		2020	
			CCAE+MDCR	MDCD	CCAE+MDCR	MDCD	CCAE+MDCR	MDCD	CCAE+MDCR	MDCD	CCAE+MDCR	MDCD
	Female /Male	% (95% CI)	9.53	6.59	10.04	7.17	10.45	6.63	11.72	7.89	13.18	8.41
50-<60	Male	% (95% CI)	0.20 (0.20-0.21)	0.62 (0.59-0.66)	0.18 (0.18-0.19)	0.59 (0.55-0.62)	0.18 (0.17-0.19)	0.60 (0.56-0.64)	0.16 (0.16-0.17)	0.58 (0.54-0.61)	0.15 (0.14-0.16)	0.49 (0.46-0.52)
		n	3,543	1,089	3,021	1,036	2,957	889	2,084	1,063	1,802	989
		N	1,733,395	175,141	1,641,470	177,094	1,646,485	147,371	1,281,760	183,685	1,209,493	203,896
	Female	% (95% CI)	2.02 (2.00-2.04)	5.32 (5.23-5.41)	1.99 (1.97-2.01)	5.22 (5.13-5.31)	1.92 (1.90-1.94)	5.42 (5.32-5.52)	1.88 (1.86-1.90)	5.29 (5.21-5.38)	1.74 (1.72-1.77)	4.66 (4.58-4.73)
		n	39,994	12,589	36,747	12,509	34,844	11,034	26,353	13,130	22,973	12,779
		N	1,979,638	236,759	1,847,821	239,740	1,816,189	203,673	1,401,479	248,036	1,317,871	274,498
	Female /Male	% (95% CI)	9.88	8.55	10.81	8.92	10.68	8.98	11.57	9.15	11.7	9.6
60-<70	Male	% (95% CI)	0.23 (0.22-0.24)	0.43 (0.38-0.48)	0.21 (0.20-0.22)	0.45 (0.40-0.50)	0.20 (0.19-0.21)	0.46 (0.41-0.51)	0.18 (0.17-0.19)	0.45 (0.41-0.50)	0.16 (0.16-0.17)	0.35 (0.32-0.39)
		n	2,486	306	2,023	355	1,828	323	1,247	447	1,152	393
		N	1,069,282	71,665	956,188	79,065	923,785	70,646	690,570	98,315	698,287	111,001
	Female	% (95% CI)	2.20 (2.18-2.23)	4.24 (4.11-4.37)	2.11 (2.09-2.14)	4.12 (4.00-4.25)	1.93 (1.91-1.96)	4.45 (4.32-4.60)	1.90 (1.87-1.93)	4.44 (4.32-4.56)	1.83 (1.80-1.86)	3.87 (3.77-3.98)
		n	27,114	3,789	22,737	3,952	19,831	3,776	14,598	5,173	14,233	5,000
		N	1,230,667	89,414	1,076,219	95,852	1,024,959	84,769	767,460	116,483	776,863	129,173
	Female /Male	% (95% CI)	9.48	9.92	9.99	9.18	9.78	9.74	10.53	9.77	11.11	10.93

Age	Sex	Statistic	2016		2017		2018		2019		2020	
			CCAE+MDCR	MDCD	CCAE+MDCR	MDCD	CCAE+MDCR	MDCD	CCAE+MDCR	MDCD	CCAE+MDCR	MDCD
70-<80	Male	% (95% CI)	0.21 (0.20-0.23)	0.16 (0.06-0.32)	0.19 (0.18-0.21)	0.26 (0.13-0.45)	0.19 (0.17-0.21)	0.35 (0.20-0.58)	0.19 (0.17-0.22)	0.33 (0.23-0.46)	0.19 (0.17-0.21)	0.22 (0.14-0.32)
		n	714	7	487	12	370	15	210	34	358	24
		N	333,765	4,506	249,760	4,614	197,632	4,241	109,623	10,289	190,250	11,062
	Female	% (95% CI)	2.03 (1.99-2.08)	1.41 (1.18-1.68)	2.08 (2.03-2.14)	1.81 (1.55-2.11)	1.77 (1.72-1.83)	1.76 (1.49-2.07)	2.10 (2.02-2.17)	2.38 (2.17-2.60)	2.15 (2.09-2.21)	2.19 (1.99-2.40)
		n	7,880	130	5,922	163	4,222	144	2,878	454	4,675	443
		N	387,660	9,191	284,192	8,981	237,957	8,185	137,233	19,097	217,811	20,250
	Female /Male	% (95% CI)	9.5	9.1	10.69	6.98	9.48	4.97	10.95	7.19	11.41	10.08
≥80	Male	% (95% CI)	0.16 (0.14-0.18)	0.08 (0.01-0.30)	0.15 (0.13-0.17)	0.00 (0.00-0.21)	0.12 (0.10-0.15)	0.00 (0.00-0.24)	0.13 (0.10-0.16)	0.15 (0.06-0.29)	0.14 (0.12-0.16)	0.13 (0.05-0.27)
		n	312	2	208	0	129	0	72	8	131	7
		N	197,439	2,389	136,702	1,757	103,286	1,506	55,484	5,375	94,980	5,282
	Female	% (95% CI)	0.92 (0.89-0.96)	0.21 (0.11-0.35)	0.92 (0.87-0.96)	0.50 (0.30-0.78)	0.89 (0.84-0.94)	0.31 (0.15-0.56)	1.09 (1.02-1.17)	0.76 (0.62-0.92)	1.10 (1.05-1.16)	0.73 (0.59-0.89)
		n	2,786	14	1,867	19	1,370	10	896	100	1,484	96
		N	301,401	6,667	203,967	3,786	154,109	3,268	81,871	13,159	134,706	13,232
	Female /Male	% (95% CI)	5.85	2.51	6.02		7.12		8.43	5.11	7.99	5.47

CCAE=Commercial Claims and Encounters database; MDCR= Medicare Supplemental and Coordination of Benefits Database; MDCD=Medicaid Database

Supplementary Table 8: Annual prevalence of diagnosed fibromyalgia (≥1 diagnosis code) by age groups and geographical regions in Commercial + Medicare databases

	2016			2017			2018			2019			2020		
	CCAE+MDCR			CCAE+MDCR			CCAE+MDCR			CCAE+MDCR			CCAE+MDCR		
**Region**	**0-<18 years**	**≥18 years**	**All ages**	**0-<18 years**	**≥18 years**	**All ages**	**0-<18 years**	**≥18 years**	**All ages**	**0-<18 years**	**≥18 years**	**All ages**	**0-<18 years**	**≥18 years**	**All ages**
Northeast Region	0.01 (0.01-0.02)	0.63 (0.62-0.64)	0.51 (0.50-0.52)	0.01 (0.01-0.02)	0.62 (0.61-0.63)	0.50 (0.49-0.51)	0.02 (0.01-0.02)	0.60 (0.59-0.61)	0.49 (0.48-0.49)	0.01 (0.01-0.01)	0.48 (0.47-0.49)	0.39 (0.38-0.40)	0.01 (0.00-0.01)	0.45 (0.44-0.46)	0.36 (0.35-0.37)
North Central Region	0.01 (0.01-0.01)	0.91 (0.90-0.92)	0.72 (0.72-0.73)	0.01 (0.01-0.01)	0.87 (0.86-0.88)	0.69 (0.68-0.69)	0.01 (0.01-0.01)	0.81 (0.80-0.82)	0.64 (0.63-0.65)	0.01 (0.01-0.01)	0.82 (0.81-0.83)	0.65 (0.64-0.66)	0.01 (0.01-0.01)	0.80 (0.79-0.81)	0.64 (0.63-0.65)
South Region	0.02 (0.02-0.02)	0.86 (0.85-0.86)	0.69 (0.68-0.69)	0.01 (0.01-0.02)	0.80 (0.80-0.81)	0.64 (0.64-0.65)	0.01 (0.01-0.02)	0.75 (0.74-0.75)	0.59 (0.59-0.60)	0.01 (0.01-0.01)	0.72 (0.71-0.73)	0.57 (0.57-0.58)	0.01 (0.01-0.01)	0.68 (0.67-0.68)	0.54 (0.53-0.54)
West Region	0.02 (0.01-0.02)	0.66 (0.65-0.67)	0.51 (0.50-0.52)	0.01 (0.01-0.02)	0.61 (0.60-0.62)	0.48 (0.47-0.48)	0.02 (0.01-0.02)	0.59 (0.58-0.60)	0.46 (0.46-0.47)	0.02 (0.01-0.02)	0.56 (0.55-0.57)	0.43 (0.43-0.44)	0.01 (0.01-0.01)	0.48 (0.47-0.49)	0.38 (0.37-0.38)
Unknown Region	0.00 (0.00-0.02)	0.65 (0.57-0.73)	0.44 (0.39-0.50)	0.00 (0.00-0.02)	0.65 (0.57-0.74)	0.43 (0.38-0.49)	0.01 (0.00-0.04)	0.56 (0.50-0.63)	0.39 (0.34-0.43)	0.01 (0.00-0.03)	0.54 (0.48-0.61)	0.36 (0.32-0.41)	0.02 (0.00-0.06)	0.35 (0.30-0.41)	0.27 (0.23-0.31)

CCAE=Commercial Claims and Encounters database; MDCR= Medicare Supplemental and Coordination of Benefits Database; MDCD=Medicaid Database

Supplementary Table 9: Annual prevalence of diagnosed fibromyalgia (≥1 diagnosis code) by age groups and race/ethnicity in the Medicaid database

Race/Ethnicity	Statistic	2016		2017		2018		2019		2020	
		0-<18 years	≥18 years	0-<18 years	≥18 years	0-<18 years	≥18 years	0-<18 years	≥18 years	0-<18 years	≥18 years
Missing	% (95% CI)			0.01 (0.01-0.01)	1.67 (1.63-1.72)	0.01 (0.01-0.01)	2.02 (1.96-2.08)	0.01 (0.01-0.02)	1.94 (1.89-2.00)	0.01 (0.00-0.01)	1.57 (1.52-1.62)
	n			42	6,375	23	4,294	36	4,718	25	4,094
	N			503,206	380,842	256,850	212,536	293,180	242,905	363,176	260,748
White	% (95% CI)	0.02 (0.01-0.02)	2.24 (2.21-2.26)	0.01 (0.01-0.02)	2.11 (2.09-2.14)	0.01 (0.01-0.02)	2.18 (2.15-2.20)	0.01 (0.01-0.01)	2.04 (2.02-2.06)	0.01 (0.01-0.01)	1.67 (1.65-1.68)
	n	344	33,369	291	31,896	236	28,847	196	30,715	154	30,402
	N	2,062,526	1,492,863	2,019,501	1,510,102	1,722,962	1,325,858	1,733,216	1,506,126	1,881,321	1,825,088
Black	% (95% CI)	0.01 (0.01-0.01)	0.80 (0.78-0.82)	0.00 (0.00-0.01)	0.75 (0.73-0.77)	0.00 (0.00-0.00)	0.76 (0.75-0.78)	0.00 (0.00-0.01)	0.74 (0.73-0.76)	0.00 (0.00-0.00)	0.60 (0.59-0.61)
	n	95	8,087	66	7,815	40	7,056	49	7,179	28	7,056
	N	1,469,794	1,010,222	1,451,080	1,043,220	1,226,642	923,301	1,244,990	966,034	1,383,986	1,175,173
Hispanic	% (95% CI)	0.01 (0.01-0.02)	1.13 (1.06-1.21)	0.00 (0.00-0.01)	1.04 (0.97-1.10)	0.00 (0.00-0.00)	0.49 (0.44-0.55)	0.00 (0.00-0.01)	1.33 (1.28-1.38)	0.00 (0.00-0.00)	1.06 (1.02-1.10)
	n	48	979	17	996	5	323	16	2,969	9	3,004
	N	394,860	86,297	382,616	95,984	281,553	65,287	491,031	223,514	519,068	283,027
Others	% (95% CI)	0.01 (0.01-0.02)	1.51 (1.47-1.54)	0.00 (0.00-0.01)	0.84 (0.78-0.91)	0.01 (0.00-0.01)	0.80 (0.74-0.87)	0.00 (0.00-0.01)	1.12 (1.07-1.16)	0.00 (0.00-0.01)	0.83 (0.80-0.87)

Race/Ethnicity	Statistic	2016		2017		2018		2019		2020	
		0-<18 years	≥18 years	0-<18 years	≥18 years	0-<18 years	≥18 years	0-<18 years	≥18 years	0-<18 years	≥18 years
	n	70	6,773	7	651	10	659	10	2,485	7	2,003
	N	576,574	449,222	152,965	77,627	151,152	82,062	264,629	222,866	276,957	241,019

CCAE=Commercial Claims and Encounters database; MDCR= Medicare Supplemental and Coordination of Benefits Database; MDCD=Medicaid Database

Supplementary Table 10: Annual incidence of diagnosed fibromyalgia (≥2 diagnosis codes) by age groups

Age	Statistic	2017		2018		2019		2020	
		CCAE+MDCR	MDCD	CCAE+MDCR	MDCD	CCAE+MDCR	MDCD	CCAE+MDCR	MDCD
All ages	% (95% CI)	0.13 (0.13-0.13)	0.13 (0.13-0.13)	0.12 (0.12-0.12)	0.14 (0.14-0.14)	0.11 (0.11-0.11)	0.13 (0.12-0.13)	0.10 (0.09-0.10)	0.12 (0.12-0.12)
	n	20,893	9,657	18,181	8,761	14,853	7,721	11,594	8,099
	N	16,418,267	7,310,523	15,542,874	6,221,065	13,693,718	6,049,978	12,072,441	6,822,550
0-<2	% (95% CI)	0.0000 (0.0000-0.0623)	0.0000 (0.0000-0.5027)	0.0000 (0.0000-0.0584)	0.0000 (0.0000-0.0600)	0.0000 (0.0000-0.0639)	0.0000 (0.0000-0.0594)	0.0000 (0.0000-0.0761)	0.0000 (0.0000-0.0634)
	n	0	0	0	0	0	0	0	0
	N	5,922	732	6,310	6,150	5,771	6,210	4,847	5,814
2-<6	% (95% CI)	0.0002 (0.0000-0.0009)	0.0000 (0.0000-0.5027)	0.0002 (0.0000-0.0009)	0.0000 (0.0000-0.0004)	0.0000 (0.0000-0.0007)	0.0001 (0.0000-0.0007)	0.0002 (0.0000-0.0011)	0.0002 (0.0000-0.0008)
	n	1	1	1	0	0	1	1	2
	N	645,085	1,069,098	635,731	873,454	544,061	834,187	485,027	883,002
6-<12	% (95% CI)	0.0010 (0.0005-0.0018)	0.0004 (0.0002-0.0009)	0.0004 (0.0001-0.0010)	0.0004 (0.0001-0.0009)	0.0010 (0.0005-0.0019)	0.0005 (0.0002-0.0010)	0.0006 (0.0002-0.0013)	0.0001 (0.0000-0.0005)
	n	12	7	5	5	10	6	5	2
	N	1,174,511	1,661,581	1,141,988	1,366,346	988,067	1,307,641	879,669	1,394,210
0-<12	% (95% CI)	0.0007 (0.0004-0.0012)	0.0003 (0.0001-0.0006)	0.0003 (0.0001-0.0007)	0.0002 (0.0001-0.0005)	0.0007 (0.0003-0.0012)	0.0003 (0.0001-0.0007)	0.0004 (0.0002-0.0010)	0.0002 (0.0000-0.0004)
	n	13	8	6	5	10	7	6	4
	N	1,825,518	2,731,411	1,784,029	2,245,950	1,537,899	2,148,038	1,369,543	2,283,026

Age	Statistic	2017		2018		2019		2020	
		CCAE+MDCR	MDCD	CCAE+MDCR	MDCD	CCAE+MDCR	MDCD	CCAE+MDCR	MDCD
12-<18	% (95% CI)	0.01 (0.01-0.01)	0.01 (0.01-0.01)	0.01 (0.01-0.01)	0.01 (0.01-0.01)	0.01 (0.01-0.01)	0.01 (0.00-0.01)	0.01 (0.01-0.01)	0.0042 (0.0032-0.0054)
	n	132	126	163	98	109	73	75	57
	N	1,390,394	1,470,430	1,354,031	1,255,537	1,195,072	1,237,419	1,069,484	1,357,103
0-<18	% (95% CI)	0.0045 (0.0038-0.0053)	0.0032 (0.0027-0.0038)	0.01 (0.00-0.01)	0.0029 (0.0024-0.0036)	0.0044 (0.0036-0.0052)	0.0024 (0.0019-0.0029)	0.0033 (0.0026-0.0041)	0.0017 (0.0013-0.0022)
	n	145	134	169	103	119	80	81	61
	N	3,215,912	4,201,841	3,138,060	3,501,487	2,732,971	3,385,457	2,439,027	3,640,129
≥18	% (95% CI)	0.16 (0.16-0.16)	0.31 (0.30-0.31)	0.15 (0.14-0.15)	0.32 (0.31-0.33)	0.13 (0.13-0.14)	0.29 (0.28-0.29)	0.12 (0.12-0.12)	0.25 (0.25-0.26)
	n	20,748	9,523	18,012	8,658	14,734	7,641	11,513	8,038
	N	13,202,355	3,108,682	12,404,814	2,719,578	10,960,747	2,664,521	9,633,414	3,182,421
18-<40	% (95% CI)	0.07 (0.07-0.07)	0.16 (0.16-0.17)	0.07 (0.07-0.07)	0.16 (0.16-0.17)	0.06 (0.06-0.06)	0.14 (0.14-0.15)	0.05 (0.05-0.06)	0.13 (0.12-0.13)
	n	3,321	3,393	3,194	2,912	2,543	2,477	2,069	2,597
	N	4,818,634	2,058,883	4,737,653	1,782,119	4,341,227	1,748,194	3,830,397	2,004,207
40-<50	% (95% CI)	0.19 (0.18-0.19)	0.58 (0.56-0.60)	0.18 (0.18-0.19)	0.61 (0.58-0.63)	0.16 (0.16-0.17)	0.54 (0.52-0.57)	0.15 (0.14-0.15)	0.47 (0.45-0.49)
	n	4,755	2,662	4,547	2,448	3,630	2,158	2,829	2,297
	N	2,540,403	457,212	2,466,538	404,336	2,211,576	396,514	1,936,117	488,369
50-<60	% (95% CI)	0.23 (0.23-0.24)	0.65 (0.63-0.68)	0.21 (0.21-0.22)	0.69 (0.66-0.71)	0.20 (0.19-0.20)	0.65 (0.63-0.68)	0.18 (0.18-0.19)	0.52 (0.50-0.54)
	n	6,904	2,522	6,050	2,359	4,921	2,142	4,008	2,113

Age	Statistic	2017		2018		2019		2020	
		CCAE+MDCR	MDCD	CCAE+MDCR	MDCD	CCAE+MDCR	MDCD	CCAE+MDCR	MDCD
	N	2,986,078	387,829	2,844,782	343,606	2,496,940	328,231	2,205,186	404,621
60-<70	% (95% CI)	0.21 (0.20-0.22)	0.50 (0.47-0.53)	0.19 (0.18-0.20)	0.53 (0.49-0.56)	0.18 (0.17-0.19)	0.47 (0.44-0.50)	0.15 (0.15-0.16)	0.40 (0.37-0.42)
	n	4,151	902	3,396	903	2,773	814	2,108	933
	N	1,987,732	180,585	1,797,609	171,663	1,535,449	173,035	1,374,971	236,126
70-<80	% (95% CI)	0.23 (0.21-0.24)	0.28 (0.20-0.38)	0.18 (0.17-0.19)	0.26 (0.18-0.37)	0.28 (0.26-0.30)	0.36 (0.26-0.47)	0.20 (0.18-0.22)	0.27 (0.22-0.34)
	n	1,168	40	611	33	655	47	362	81
	N	513,474	14,328	340,028	12,606	234,657	13,203	181,478	29,919
≥80	% (95% CI)	0.13 (0.11-0.14)	0.04 (0.01-0.10)	0.10 (0.09-0.11)	0.06 (0.01-0.17)	0.15 (0.13-0.17)	0.06 (0.01-0.16)	0.13 (0.11-0.15)	0.09 (0.05-0.14)
	n	449	4	214	3	212	3	137	17
	N	356,034	9,845	218,204	5,248	140,898	5,344	105,265	19,179

CCAE=Commercial Claims and Encounters database; MDCR= Medicare Supplemental and Coordination of Benefits Database; MDCD=Medicaid Database

Supplementary Table 11: Annual incidence of diagnosed fibromyalgia (≥2 diagnosis codes) by age groups and sex

Age	Sex	Statistic	2017		2018		2019		2020	
			CCAE+MDCR	MDCD	CCAE+MDCR	MDCD	CCAE+MDCR	MDCD	CCAE+MDCR	MDCD
All ages	Male	% (95% CI)	0.03 (0.02-0.03)	0.02 (0.02-0.03)	0.02 (0.02-0.02)	0.02 (0.02-0.03)	0.02 (0.02-0.02)	0.02 (0.02-0.02)	0.02 (0.02-0.02)	0.02 (0.02-0.02)
		n	2,001	813	1,692	664	1,318	611	1,037	592
		N	7,938,844	3,261,866	7,563,992	2,800,795	6,732,714	2,699,865	5,929,771	3,055,014
	Female	% (95% CI)	0.22 (0.22-0.23)	0.22 (0.21-0.22)	0.21 (0.20-0.21)	0.24 (0.23-0.24)	0.19 (0.19-0.20)	0.21 (0.21-0.22)	0.17 (0.17-0.18)	0.20 (0.19-0.20)
		n	18,892	8,844	16,489	8,097	13,535	7,110	10,557	7,507
		N	8,479,423	4,048,657	7,978,882	3,420,270	6,961,004	3,350,113	6,142,670	3,767,536
	Female/Male	% (95% CI)	8.84	8.76	9.24	9.99	9.93	9.38	9.83	10.28
0-<2	Male	% (95% CI)	0.0000 (0.0000-0.1198)	0.0000 (0.0000-0.9763)	0.0000 (0.0000-0.1150)	0.0000 (0.0000-0.1182)	0.0000 (0.0000-0.1236)	0.0000 (0.0000-0.1169)	0.0000 (0.0000-0.1482)	0.0000 (0.0000-0.1249)
		n	0	0	0	0	0	0	0	0
		N	3,077	376	3,207	3,118	2,983	3,154	2,487	2,952
	Female	% (95% CI)	0.0000 (0.0000-0.1296)	0.0000 (0.0000-1.0309)	0.0000 (0.0000-0.1188)	0.0000 (0.0000-0.1216)	0.0000 (0.0000-0.1322)	0.0000 (0.0000-0.1206)	0.0000 (0.0000-0.1562)	0.0000 (0.0000-0.1288)
		n	0	0	0	0	0	0	0	0
		N	2,845	356	3,103	3,032	2,788	3,056	2,360	2,862
	Female/Male	% (95% CI)								
2-<6	Male	% (95% CI)	0.0000 (0.0000-0.0011)	0.0002 (0.0000-0.0010)	0.0000 (0.0000-0.0011)	0.0000 (0.0000-0.0008)	0.0000 (0.0000-0.0013)	0.0002 (0.0000-0.0013)	0.0000 (0.0000-0.0015)	0.0004 (0.0001-0.0016)

Age	Sex	Statistic	2017		2018		2019		2020	
			CCAE+MDCR	MDCD	CCAE+MDCR	MDCD	CCAE+MDCR	MDCD	CCAE+MDCR	MDCD
		n	0	1	0	0	0	1	0	2
		N	330,287	547,187	325,398	447,164	278,007	427,637	248,257	452,529
	Female	% (95% CI)	0.0003 (0.0000-0.0018)	0.0000 (0.0000-0.0007)	0.0003 (0.0000-0.0018)	0.0000 (0.0000-0.0009)	0.0000 (0.0000-0.0014)	0.0000 (0.0000-0.0009)	0.0004 (0.0000-0.0024)	0.0000 (0.0000-0.0009)
		n	1	0	1	0	0	0	1	0
		N	314,798	521,911	310,333	426,290	266,054	406,550	236,770	430,473
	Female/Male	% (95% CI)		0.00				0.00		0.00
6-<12	Male	% (95% CI)	0.0008 (0.0003-0.0019)	0.0003 (0.0001-0.0010)	0.0002 (0.0000-0.0010)	0.0001 (0.0000-0.0008)	0.0016 (0.0007-0.0031)	0.0006 (0.0002-0.0015)	0.0004 (0.0001-0.0016)	0.0003 (0.0000-0.0010)
		n	5	3	1	1	8	4	2	2
		N	600,503	857,529	583,809	706,871	504,923	676,918	449,505	720,992
	Female	% (95% CI)	0.0012 (0.0005-0.0025)	0.0005 (0.0001-0.0013)	0.0007 (0.0002-0.0018)	0.0006 (0.0002-0.0016)	0.0004 (0.0001-0.0015)	0.0003 (0.0000-0.0011)	0.0007 (0.0001-0.0020)	0.0000 (0.0000-0.0005)
		n	7	4	4	4	2	2	3	0
		N	574,008	804,052	558,179	659,475	483,144	630,723	430,164	673,218
	Female/Male	% (95% CI)	1.46	1.42	4.18	4.29	0.26	0.54	1.57	0.00
0-<12	Male	% (95% CI)	0.0005 (0.0002-0.0012)	0.0003 (0.0001-0.0007)	0.0001 (0.0000-0.0006)	0.0001 (0.0000-0.0005)	0.0010 (0.0004-0.0020)	0.0005 (0.0001-0.0011)	0.0003 (0.0000-0.0010)	0.0003 (0.0001-0.0009)
		n	5	4	1	1	8	5	2	4
		N	933,867	1,405,092	912,414	1,157,153	785,913	1,107,709	700,249	1,176,473

Age	Sex	Statistic	2017		2018		2019		2020	
			CCAE+MDCR	MDCD	CCAE+MDCR	MDCD	CCAE+MDCR	MDCD	CCAE+MDCR	MDCD
	Female	% (95% CI)	0.0009 (0.0004-0.0018)	0.0003 (0.0001-0.0008)	0.0006 (0.0002-0.0013)	0.0004 (0.0001-0.0009)	0.0003 (0.0000-0.0010)	0.0002 (0.0000-0.0007)	0.0006 (0.0002-0.0015)	0.0000 (0.0000-0.0003)
		n	8	4	5	4	2	2	4	0
		N	891,651	1,326,319	871,615	1,088,797	751,986	1,040,329	669,294	1,106,553
	Female/Male	% (95% CI)	1.68	1.06	5.23	4.25	0.26	0.43	2.09	0.00
12-<18	Male	% (95% CI)	0.0028 (0.0017-0.0044)	0.0028 (0.0017-0.0042)	0.0032 (0.0020-0.0048)	0.0014 (0.0006-0.0026)	0.0023 (0.0013-0.0039)	0.0019 (0.0010-0.0033)	0.0026 (0.0014-0.0043)	0.0007 (0.0002-0.0017)
		n	20	21	22	9	14	12	14	5
		N	708,397	759,625	690,876	650,204	609,938	641,518	545,697	702,805
	Female	% (95% CI)	0.02 (0.01-0.02)	0.01 (0.01-0.02)	0.02 (0.02-0.03)	0.01 (0.01-0.02)	0.02 (0.01-0.02)	0.01 (0.01-0.01)	0.01 (0.01-0.01)	0.01 (0.01-0.01)
		n	112	105	141	89	95	61	61	52
		N	681,997	710,805	663,155	605,333	585,134	595,901	523,787	654,298
	Female/Male	% (95% CI)	5.82	5.34	6.68	10.62	7.07	5.47	4.54	11.17
0-<18	Male	% (95% CI)	0.0015 (0.0010-0.0022)	0.0012 (0.0007-0.0017)	0.0014 (0.0009-0.0022)	0.0006 (0.0003-0.0010)	0.0016 (0.0010-0.0024)	0.0010 (0.0006-0.0016)	0.0013 (0.0007-0.0021)	0.0005 (0.0002-0.0009)
		n	25	25	23	10	22	17	16	9
		N	1,642,264	2,164,717	1,603,290	1,807,357	1,395,851	1,749,227	1,245,946	1,879,278
	Female	% (95% CI)	0.01 (0.01-0.01)	0.01 (0.00-0.01)	0.01 (0.01-0.01)	0.01 (0.00-0.01)	0.01 (0.01-0.01)	0.0039 (0.0030-0.0049)	0.01 (0.00-0.01)	0.0030 (0.0022-0.0039)

Age	Sex	Statistic	2017		2018		2019		2020	
			CCAE+MDCR	MDCD	CCAE+MDCR	MDCD	CCAE+MDCR	MDCD	CCAE+MDCR	MDCD
		n	120	109	146	93	97	63	65	52
		N	1,573,648	2,037,124	1,534,770	1,694,130	1,337,120	1,636,230	1,193,081	1,760,851
	Female/Male	% (95% CI)	5.01	4.63	6.63	9.92	4.60	3.96	4.24	6.17
≥18	Male	% (95% CI)	0.03 (0.03-0.03)	0.07 (0.07-0.08)	0.03 (0.03-0.03)	0.07 (0.06-0.07)	0.02 (0.02-0.03)	0.06 (0.06-0.07)	0.02 (0.02-0.02)	0.05 (0.05-0.05)
		n	1,976	788	1,669	654	1,296	594	1,021	583
		N	6,296,580	1,097,149	5,960,702	993,438	5,336,863	950,638	4,683,825	1,175,736
	Female	% (95% CI)	0.27 (0.27-0.28)	0.43 (0.43-0.44)	0.25 (0.25-0.26)	0.46 (0.45-0.47)	0.24 (0.23-0.24)	0.41 (0.40-0.42)	0.21 (0.21-0.22)	0.37 (0.36-0.38)
		n	18,772	8,735	16,343	8,004	13,438	7,047	10,492	7,455
		N	6,905,775	2,011,533	6,444,112	1,726,140	5,623,884	1,713,883	4,949,589	2,006,685
	Female/Male	% (95% CI)	8.66	6.05	9.06	7.04	9.84	6.58	9.72	7.49
18-<40	Male	% (95% CI)	0.02 (0.01-0.02)	0.03 (0.03-0.04)	0.01 (0.01-0.02)	0.03 (0.03-0.04)	0.01 (0.01-0.01)	0.03 (0.02-0.03)	0.01 (0.01-0.01)	0.02 (0.02-0.02)
		n	374	214	335	189	233	163	217	146
		N	2,355,212	684,791	2,319,798	618,859	2,167,754	594,149	1,907,196	705,706
	Female	% (95% CI)	0.12 (0.12-0.12)	0.23 (0.22-0.24)	0.12 (0.11-0.12)	0.23 (0.23-0.24)	0.11 (0.10-0.11)	0.20 (0.19-0.21)	0.10 (0.09-0.10)	0.19 (0.18-0.20)
		n	2,947	3,179	2,859	2,723	2,310	2,314	1,852	2,451
		N	2,463,422	1,374,092	2,417,855	1,163,260	2,173,473	1,154,045	1,923,201	1,298,501
	Female/Male	% (95% CI)	7.53	7.40	8.19	7.66	9.89	7.31	8.46	9.12

Age	Sex	Statistic	2017		2018		2019		2020	
			CCAE+MDCR	MDCD	CCAE+MDCR	MDCD	CCAE+MDCR	MDCD	CCAE+MDCR	MDCD
40-<50	Male	% (95% CI)	0.04 (0.04-0.04)	0.14 (0.12-0.16)	0.04 (0.03-0.04)	0.13 (0.11-0.15)	0.03 (0.03-0.03)	0.12 (0.11-0.15)	0.02 (0.02-0.03)	0.10 (0.08-0.11)
		n	467	217	417	181	312	163	203	165
		N	1,202,404	155,431	1,172,013	140,318	1,068,158	130,683	931,348	169,236
	Female	% (95% CI)	0.32 (0.31-0.33)	0.81 (0.78-0.84)	0.32 (0.31-0.33)	0.86 (0.82-0.89)	0.29 (0.28-0.30)	0.75 (0.72-0.78)	0.26 (0.25-0.27)	0.67 (0.64-0.70)
		n	4,288	2,445	4,130	2,267	3,318	1,995	2,626	2,132
		N	1,337,999	301,781	1,294,525	264,018	1,143,418	265,831	1,004,769	319,133
	Female/Male	% (95% CI)	8.25	5.80	8.97	6.66	9.93	6.02	11.99	6.85
50-<60	Male	% (95% CI)	0.04 (0.04-0.05)	0.15 (0.13-0.17)	0.04 (0.04-0.04)	0.14 (0.12-0.16)	0.04 (0.03-0.04)	0.14 (0.12-0.16)	0.03 (0.03-0.04)	0.10 (0.09-0.12)
		n	586	249	540	203	428	193	366	178
		N	1,411,598	166,752	1,358,149	148,371	1,203,320	138,582	1,065,008	173,379
	Female	% (95% CI)	0.40 (0.39-0.41)	1.03 (0.99-1.07)	0.37 (0.36-0.38)	1.10 (1.06-1.15)	0.35 (0.34-0.36)	1.03 (0.98-1.07)	0.32 (0.31-0.33)	0.84 (0.80-0.87)
		n	6,318	2,273	5,510	2,156	4,493	1,949	3,642	1,935
		N	1,574,480	221,077	1,486,633	195,235	1,293,620	189,649	1,140,178	231,242
	Female/Male	% (95% CI)	9.67	6.89	9.32	8.07	9.76	7.38	9.29	8.15
60-<70	Male	% (95% CI)	0.04 (0.04-0.05)	0.12 (0.10-0.15)	0.04 (0.03-0.04)	0.10 (0.08-0.12)	0.04 (0.03-0.04)	0.09 (0.07-0.11)	0.03 (0.03-0.03)	0.08 (0.06-0.10)
		n	394	101	311	77	258	70	197	88
		N	939,115	82,726	862,038	79,875	734,406	80,858	657,657	110,998

Age	Sex	Statistic	2017		2018		2019		2020	
			CCAE+MDCR	MDCD	CCAE+MDCR	MDCD	CCAE+MDCR	MDCD	CCAE+MDCR	MDCD
	Female	% (95% CI)	0.36 (0.35-0.37)	0.82 (0.76-0.88)	0.33 (0.32-0.34)	0.90 (0.84-0.96)	0.31 (0.30-0.33)	0.81 (0.75-0.87)	0.27 (0.25-0.28)	0.68 (0.63-0.72)
		n	3,757	801	3,085	826	2,515	744	1,911	845
		N	1,048,617	97,859	935,571	91,788	801,043	92,177	717,314	125,128
	Female/Male	% (95% CI)	8.54	6.70	9.14	9.33	8.94	9.32	8.89	8.52
70-<80	Male	% (95% CI)	0.04 (0.03-0.05)	0.15 (0.06-0.30)	0.03 (0.02-0.04)	0.09 (0.03-0.24)	0.04 (0.03-0.05)	0.11 (0.03-0.25)	0.03 (0.02-0.05)	0.05 (0.02-0.11)
		n	104	7	45	4	41	5	26	5
		N	243,556	4,799	158,817	4,348	105,347	4,689	80,492	10,774
	Female	% (95% CI)	0.39 (0.37-0.42)	0.35 (0.24-0.49)	0.31 (0.29-0.34)	0.35 (0.24-0.50)	0.47 (0.44-0.51)	0.49 (0.36-0.67)	0.33 (0.30-0.37)	0.40 (0.31-0.50)
		n	1,064	33	566	29	614	42	336	76
		N	269,918	9,529	181,211	8,258	129,310	8,514	100,986	19,145
	Female/Male	% (95% CI)	9.23	2.37	11.02	3.82	12.20	4.63	10.30	8.55
≥80	Male	% (95% CI)	0.04 (0.03-0.05)	0.00 (0.00-0.14)	0.02 (0.01-0.04)	0.00 (0.00-0.22)	0.04 (0.03-0.06)	0.00 (0.00-0.22)	0.03 (0.01-0.05)	0.02 (0.00-0.10)
		n	51	0	21	0	24	0	12	1
		N	144,695	2,650	89,887	1,667	57,878	1,677	42,124	5,643
	Female	% (95% CI)	0.19 (0.17-0.21)	0.06 (0.02-0.14)	0.15 (0.13-0.17)	0.08 (0.02-0.24)	0.23 (0.20-0.26)	0.08 (0.02-0.24)	0.20 (0.16-0.24)	0.12 (0.07-0.19)
		n	398	4	193	3	188	3	125	16
		N	211,339	7,195	128,317	3,581	83,020	3,667	63,141	13,536

Age	Sex	Statistic	2017		2018		2019		2020	
			CCAE+MDCR	MDCD	CCAE+MDCR	MDCD	CCAE+MDCR	MDCD	CCAE+MDCR	MDCD
	Female/Male	% (95% CI)	5.34		6.44		5.46		6.95	6.67

CCAE=Commercial Claims and Encounters database; MDCR= Medicare Supplemental and Coordination of Benefits Database; MDCD=Medicaid Database

Supplementary Table 12: Annual incidence of diagnosed fibromyalgia (≥2 diagnosis codes) by age groups and geographical regions in Commercial + Medicare databases

		2017			2018			2019			2020		
**Regions**		**0-<18 years**	**≥18 years**	**All ages**	**0-<18 years**	**≥18 years**	**All ages**	**0-<18 years**	**≥18 years**	**All ages**	**0-<18 years**	**≥18 years**	**All ages**
Northeast Region	% (95% CI)	0.00 (0.00-0.01)	0.14 (0.14-0.15)	0.12 (0.11-0.12)	0.01 (0.00-0.01)	0.12 (0.12-0.13)	0.10 (0.10-0.11)	0.00 (0.00-0.01)	0.10 (0.09-0.10)	0.08 (0.07-0.08)	0.00 (0.00-0.00)	0.09 (0.08-0.09)	0.07 (0.07-0.07)
North Central Region	% (95% CI)	0.00 (0.00-0.01)	0.18 (0.18-0.19)	0.15 (0.14-0.15)	0.00 (0.00-0.01)	0.16 (0.16-0.17)	0.13 (0.13-0.13)	0.00 (0.00-0.01)	0.16 (0.16-0.17)	0.13 (0.13-0.14)	0.00 (0.00-0.00)	0.14 (0.13-0.14)	0.11 (0.11-0.12)
South Region	% (95% CI)	0.00 (0.00-0.01)	0.16 (0.16-0.17)	0.13 (0.13-0.13)	0.01 (0.00-0.01)	0.15 (0.15-0.16)	0.12 (0.12-0.13)	0.00 (0.00-0.00)	0.14 (0.14-0.14)	0.11 (0.11-0.12)	0.00 (0.00-0.00)	0.13 (0.12-0.13)	0.10 (0.10-0.10)
West Region	% (95% CI)	0.01 (0.00-0.01)	0.13 (0.12-0.13)	0.10 (0.10-0.11)	0.01 (0.00-0.01)	0.13 (0.12-0.13)	0.10 (0.10-0.10)	0.01 (0.00-0.01)	0.11 (0.10-0.11)	0.09 (0.08-0.09)	0.01 (0.00-0.01)	0.10 (0.09-0.10)	0.08 (0.07-0.08)
Unknown Region	% (95% CI)	0.00 (0.00-0.02)	0.14 (0.11-0.19)	0.10 (0.07-0.13)	0.00 (0.00-0.02)	0.10 (0.07-0.14)	0.07 (0.05-0.10)	0.00 (0.00-0.03)	0.11 (0.08-0.14)	0.08 (0.06-0.10)	0.01 (0.00-0.07)	0.08 (0.05-0.13)	0.07 (0.04-0.10)

Supplementary Table 13: Annual incidence of diagnosed fibromyalgia (≥2 diagnosis codes) by age groups and race/ethnicity in the Medicaid database

		MDCD		MDCD		MDCD		MDCD	
		2017		2018		2019		2020	
		0-<18 years	≥18 years	0-<18 years	≥18 years	0-<18 years	≥18 years	0-<18 years	≥18 years
Missing	% (95% CI)	0.003 (0.002-0.01)	0.34 (0.32-0.36)	0.003 (0.001-0.01)	0.38 (0.36-0.41)	0.01 (0.003-0.01)	0.38 (0.36-0.41)	0.001 (0.000-0.003)	0.30 (0.28-0.33)
	n	12	12	8	877	14	807	2	704
	N	404,541	357,628	231,482	229,753	239,955	210,946	260,916	232,043
White	% (95% CI)	0.005 (0.004-0.01)	0.42 (0.41-0.43)	0.005 (0.004-0.01)	0.44 (0.43-0.45)	0.003 (0.002-0.004)	0.38 (0.37-0.39)	0.003 (0.002-0.004)	0.35 (0.34-0.36)
	n	93	6402	79	6089	49	5043	47	5241
	N	1,923,407	1,525,714	1,681,032	1,390,684	1,558,037	1,314,467	1,571,982	1,513,948
Black	% (95% CI)	0.002 (0.001-0.002)	0.15 (0.14-0.15)	0.001 (0.001-0.002)	0.16 (0.15-0.16)	0.001 (0.001-0.002)	0.15 (0.14-0.15)	0.001 (0.000-0.001)	0.13 (0.12-0.13)
	n	22	1548	12	1478	12	1389	7	1261
	N	1,369,120	1,054,683	1,177,359	945,111	1,139,153	950,707	1,147,880	994,487
Hispanic	% (95% CI)	0.001 (0.000-0.003)	0.22 (0.19-0.25)	0.000 (0.000-0.001)	0.10 (0.08-0.12)	0.001 (0.000-0.003)	0.24 (0.22-0.28)	0.001 (0.000-0.002)	0.21 (0.19-0.23)
	n	5	213	0	73	4	256	4	493
	N	367,569	96,928	272,943	73,425	306,633	104,717	435,850	238,412
Others	% (95% CI)	0.001 (0.000-0.01)	0.20 (0.17-0.24)	0.003 (0.001-0.01)	0.17 (0.15-0.21)	0.001 (0.000-0.004)	0.17 (0.15-0.21)	0.000 (0.000-0.002)	0.17 (0.15-0.19)
	n	2	148	4	141	1	146	1	339
	N	137,328	73,729	138,768	80,605	141,708	83,684	223,501	203,531

MDCD=Medicaid Database

Supplementary Table 14: Annual incidence of diagnosed fibromyalgia (≥1 diagnosis code) by age groups

Age	Statistic	2017		2018		2019		2020	
		CCAE+MDCR	MDCD	CCAE+MDCR	MDCD	CCAE+MDCR	MDCD	CCAE+MDCR	MDCD
All ages	% (95% CI)	0.24 (0.24-0.24)	0.23 (0.22-0.23)	0.22 (0.22-0.23)	0.23 (0.23-0.24)	0.21 (0.20-0.21)	0.22 (0.22-0.22)	0.18 (0.18-0.18)	0.20 (0.20-0.21)
	n	39,270	16,518	34,936	14,604	28,084	13,330	21,703	13,792
	N	16,418,267	7,310,523	15,542,874	6,221,065	13,693,718	6,049,978	12,072,441	6,822,550
0-<2	% (95% CI)	0.00 (0.00-0.06)	0.00 (0.00-0.50)	0.00 (0.00-0.06)	0.00 (0.00-0.06)	0.00 (0.00-0.06)	0.00 (0.00-0.06)	0.00 (0.00-0.08)	0.00 (0.00-0.06)
	n	0	0	0	0	0	0	0	0
	N	5,922	732	6,310	6,150	5,771	6,210	4,847	5,814
2-<6	% (95% CI)	0.00 (0.00-0.00)	0.00 (0.00-0.00)	0.00 (0.00-0.00)	0.00 (0.00-0.00)	0.00 (0.00-0.00)	0.00 (0.00-0.00)	0.00 (0.00-0.00)	0.00 (0.00-0.00)
	n	8	7	9	5	1	1	4	7
	N	645,085	1,069,098	635,731	873,454	544,061	834,187	485,027	883,002
6-<12	% (95% CI)	0.00 (0.00-0.00)	0.00 (0.00-0.00)	0.00 (0.00-0.00)	0.00 (0.00-0.00)	0.00 (0.00-0.00)	0.00 (0.00-0.00)	0.00 (0.00-0.00)	0.00 (0.00-0.00)
	n	30	33	28	28	27	16	9	11
	N	1,174,511	1,661,581	1,141,988	1,366,346	988,067	1,307,641	879,669	1,394,210
0-<12	% (95% CI)	0.00 (0.00-0.00)	0.00 (0.00-0.00)	0.00 (0.00-0.00)	0.00 (0.00-0.00)	0.00 (0.00-0.00)	0.00 (0.00-0.00)	0.00 (0.00-0.00)	0.00 (0.00-0.00)
	n	38	40	37	33	28	17	13	18
	N	1,825,518	2,731,411	1,784,029	2,245,950	1,537,899	2,148,038	1,369,543	2,283,026
12-<18	% (95% CI)	0.02 (0.02-0.02)	0.02 (0.01-0.02)	0.02 (0.02-0.02)	0.01 (0.01-0.02)	0.02 (0.01-0.02)	0.01 (0.01-0.01)	0.01 (0.01-0.01)	0.01 (0.01-0.01)
	n	257	229	284	182	193	157	130	98

Age	Statistic	2017		2018		2019		2020	
		CCAE+MDCR	MDCD	CCAE+MDCR	MDCD	CCAE+MDCR	MDCD	CCAE+MDCR	MDCD
	N	1,390,394	1,470,430	1,354,031	1,255,537	1,195,072	1,237,419	1,069,484	1,357,103
0-<18	% (95% CI)	0.01 (0.01-0.01)	0.01 (0.01-0.01)	0.01 (0.01-0.01)	0.01 (0.01-0.01)	0.01 (0.01-0.01)	0.01 (0.00-0.01)	0.01 (0.00-0.01)	0.00 (0.00-0.00)
	n	295	269	321	215	221	174	143	116
	N	3,215,912	4,201,841	3,138,060	3,501,487	2,732,971	3,385,457	2,439,027	3,640,129
≥18	% (95% CI)	0.30 (0.29-0.30)	0.52 (0.51-0.53)	0.28 (0.28-0.28)	0.53 (0.52-0.54)	0.25 (0.25-0.26)	0.49 (0.49-0.50)	0.22 (0.22-0.23)	0.43 (0.42-0.44)
	n	38,975	16,249	34,615	14,389	27,863	13,156	21,560	13,676
	N	13,202,355	3,108,682	12,404,814	2,719,578	10,960,747	2,664,521	9,633,414	3,182,421
18-<40	% (95% CI)	0.13 (0.12-0.13)	0.28 (0.27-0.29)	0.12 (0.12-0.13)	0.27 (0.26-0.28)	0.11 (0.11-0.11)	0.24 (0.23-0.25)	0.10 (0.10-0.10)	0.21 (0.21-0.22)
	n	6,087	5,725	5,815	4,764	4,713	4,163	3,771	4,295
	N	4,818,634	2,058,883	4,737,653	1,782,119	4,341,227	1,748,194	3,830,397	2,004,207
40-<50	% (95% CI)	0.33 (0.32-0.34)	0.98 (0.95-1.01)	0.33 (0.33-0.34)	1.00 (0.97-1.03)	0.30 (0.29-0.30)	0.92 (0.89-0.95)	0.26 (0.25-0.27)	0.77 (0.74-0.79)
	n	8,341	4,493	8,212	4,037	6,584	3,634	5,066	3,739
	N	2,540,403	457,212	2,466,538	404,336	2,211,576	396,514	1,936,117	488,369
50-<60	% (95% CI)	0.42 (0.42-0.43)	1.12 (1.09-1.16)	0.41 (0.40-0.42)	1.14 (1.11-1.18)	0.37 (0.36-0.38)	1.14 (1.11-1.18)	0.34 (0.33-0.35)	0.93 (0.90-0.96)
	n	12,657	4,357	11,660	3,933	9,287	3,753	7,512	3,764
	N	2,986,078	387,829	2,844,782	343,606	2,496,940	328,231	2,205,186	404,621
60-<70	% (95% CI)	0.43 (0.42-0.43)	0.88 (0.83-0.92)	0.39 (0.38-0.40)	0.92 (0.88-0.97)	0.37 (0.36-0.38)	0.88 (0.84-0.93)	0.31 (0.30-0.32)	0.72 (0.69-0.76)
	n	8,463	1,583	7,042	1,587	5,627	1,523	4,263	1,704

Age	Statistic	2017		2018		2019		2020	
		CCAE+MDCR	MDCD	CCAE+MDCR	MDCD	CCAE+MDCR	MDCD	CCAE+MDCR	MDCD
	N	1,987,732	180,585	1,797,609	171,663	1,535,449	173,035	1,374,971	236,126
70-<80	% (95% CI)	0.48 (0.46-0.50)	0.55 (0.44-0.69)	0.41 (0.39-0.43)	0.50 (0.38-0.64)	0.52 (0.49-0.55)	0.58 (0.46-0.73)	0.38 (0.36-0.41)	0.47 (0.40-0.56)
	n	2,458	79	1,392	63	1,222	77	697	141
	N	513,474	14,328	340,028	12,606	234,657	13,203	181,478	29,919
≥80	% (95% CI)	0.27 (0.26-0.29)	0.12 (0.06-0.21)	0.23 (0.21-0.25)	0.10 (0.03-0.22)	0.31 (0.28-0.34)	0.11 (0.04-0.24)	0.24 (0.21-0.27)	0.17 (0.12-0.24)
	n	969	12	494	5	430	6	251	33
	N	356,034	9,845	218,204	5,248	140,898	5,344	105,265	19,179

CCAE=Commercial Claims and Encounters database; MDCR= Medicare Supplemental and Coordination of Benefits Database; MDCD=Medicaid Database

Supplementary Table 15: Annual incidence of diagnosed fibromyalgia (≥1 diagnosis code) by age groups and sex

Age	Sex	Statistic	2017		2018		2019		2020	
			CCAE+MDCR	MDCD	CCAE+MDCR	MDCD	CCAE+MDCR	MDCD	CCAE+MDCR	MDCD
All ages	Male	% (95% CI)	0.06 (0.05-0.06)	0.05 (0.04-0.05)	0.05 (0.05-0.05)	0.04 (0.04-0.05)	0.04 (0.04-0.05)	0.04 (0.04-0.05)	0.04 (0.04-0.04)	0.04 (0.04-0.04)
		n	4,409	1,524	3,849	1,254	2,947	1,193	2,264	1,162
		N	7,938,844	3,261,866	7,563,992	2,800,795	6,732,714	2,699,865	5,929,771	3,055,014
	Female	% (95% CI)	43.95 (43.50-44.42)	41.06 (40.40-41.72)	41.69 (41.23-42.16)	43.91 (43.18-44.66)	39.29 (38.81-39.78)	40.73 (40.01-41.46)	33.89 (33.41-34.36)	35.48 (34.87-36.10)
		n	34,861	14,994	31,087	13,350	25,137	12,137	19,439	12,630
		N	8,479,423	4,048,657	7,978,882	3,420,270	6,961,004	3,350,113	6,142,670	3,767,536
	Female/Male	% (95% CI)	7.4	7.93	7.66	8.72	8.25	8.2	8.29	8.81
0-<2	Male	% (95% CI)	0.00 (0.00-0.12)	0.00 (0.00-0.98)	0.00 (0.00-0.11)	0.00 (0.00-0.12)	0.00 (0.00-0.12)	0.00 (0.00-0.12)	0.00 (0.00-0.15)	0.00 (0.00-0.12)
		n	0	0	0	0	0	0	0	0
		N	3,077	376	3,207	3,118	2,983	3,154	2,487	2,952
	Female	% (95% CI)	0.00 (-)	0.00 (-)	0.00 (-)	0.00 (-)	0.00 (-)	0.00 (-)	0.00 (-)	0.00 (-)
		n	0	0	0	0	0	0	0	0
		N	2,845	356	3,103	3,032	2,788	3,056	2,360	2,862
	Female/Male	% (95% CI)								
2-<6	Male	% (95% CI)	0.00 (0.00-0.00)	0.00 (0.00-0.00)	0.00 (0.00-0.00)	0.00 (0.00-0.00)	0.00 (0.00-0.00)	0.00 (0.00-0.00)	0.00 (0.00-0.00)	0.00 (0.00-0.00)
		n	3	4	4	2	0	1	2	2

Age	Sex	Statistic	2017		2018		2019		2020	
			CCAE+MDCR	MDCD	CCAE+MDCR	MDCD	CCAE+MDCR	MDCD	CCAE+MDCR	MDCD
		N	330,287	547,187	325,398	447,164	278,007	427,637	248,257	452,529
	Female	% (95% CI)	0.17 (0.07-0.41)	0.06 (0.02-0.19)	0.17 (0.07-0.41)	0.08 (0.02-0.24)	0.04 (0.01-0.29)	0.00 (-)	0.09 (0.02-0.36)	0.12 (0.05-0.29)
		n	5	3	5	3	1	0	2	5
		N	314,798	521,911	310,333	426,290	266,054	406,550	236,770	430,473
	Female/Male	% (95% CI)	1.75	0.79	1.31	1.57		0	1.05	2.63
6-<12	Male	% (95% CI)	0.00 (0.00-0.00)	0.00 (0.00-0.00)	0.00 (0.00-0.00)	0.00 (0.00-0.00)	0.00 (0.00-0.00)	0.00 (0.00-0.00)	0.00 (0.00-0.00)	0.00 (0.00-0.00)
		n	14	14	10	9	15	10	3	6
		N	600,503	857,529	583,809	706,871	504,923	676,918	449,505	720,992
	Female	% (95% CI)	0.29 (0.18-0.48)	0.25 (0.16-0.40)	0.34 (0.21-0.54)	0.31 (0.20-0.49)	0.27 (0.15-0.47)	0.10 (0.05-0.23)	0.15 (0.07-0.33)	0.08 (0.03-0.19)
		n	16	19	18	19	12	6	6	5
		N	574,008	804,052	558,179	659,475	483,144	630,723	430,164	673,218
	Female/Male	% (95% CI)	1.2	1.45	1.88	2.26	0.84	0.64	2.09	0.89
0-<12	Male	% (95% CI)	0.00 (0.00-0.00)	0.00 (0.00-0.00)	0.00 (0.00-0.00)	0.00 (0.00-0.00)	0.00 (0.00-0.00)	0.00 (0.00-0.00)	0.00 (0.00-0.00)	0.00 (0.00-0.00)
		n	17	18	14	11	15	11	5	8
		N	933,867	1,405,092	912,414	1,157,153	785,913	1,107,709	700,249	1,176,473
	Female	% (95% CI)	0.25 (0.16-0.38)	0.18 (0.12-0.27)	0.28 (0.19-0.42)	0.22 (0.15-0.34)	0.19 (0.11-0.32)	0.06 (0.03-0.14)	0.13 (0.06-0.25)	0.09 (0.05-0.17)
		n	21	22	23	22	13	6	8	10
		N	891,651	1,326,319	871,615	1,088,797	751,986	1,040,329	669,294	1,106,553

Age	Sex	Statistic	2017		2018		2019		2020	
			CCAE+MDCR	MDCD	CCAE+MDCR	MDCD	CCAE+MDCR	MDCD	CCAE+MDCR	MDCD
	Female/Male	% (95% CI)	1.29	1.29	1.72	2.13	0.91	0.58	1.67	1.33
12-<18	Male	% (95% CI)	0.01 (0.00-0.01)	0.01 (0.00-0.01)	0.01 (0.01-0.01)	0.00 (0.00-0.01)	0.01 (0.00-0.01)	0.00 (0.00-0.01)	0.01 (0.00-0.01)	0.00 (0.00-0.00)
		n	45	39	50	22	33	26	29	11
		N	708,397	759,625	690,876	650,204	609,938	641,518	545,697	702,805
	Female	% (95% CI)	3.26 (2.85-3.74)	2.87 (2.49-3.31)	3.71 (3.26-4.21)	2.87 (2.46-3.35)	2.91 (2.49-3.40)	2.39 (2.01-2.83)	2.02 (1.66-2.46)	1.37 (1.11-1.70)
		n	212	190	234	160	160	131	101	87
		N	681,997	710,805	663,155	605,333	585,134	595,901	523,787	654,298
	Female/Male	% (95% CI)	4.89	5.21	4.88	7.81	5.05	5.42	3.63	8.5
0-<18	Male	% (95% CI)	0.00 (0.00-0.00)	0.00 (0.00-0.00)	0.00 (0.00-0.01)	0.00 (0.00-0.00)	0.00 (0.00-0.00)	0.00 (0.00-0.00)	0.00 (0.00-0.00)	0.00 (0.00-0.00)
		n	62	57	64	33	48	37	34	19
		N	1,642,264	2,164,717	1,603,290	1,807,357	1,395,851	1,749,227	1,245,946	1,879,278
	Female	% (95% CI)	1.56 (1.38-1.78)	1.12 (0.98-1.29)	1.77 (1.57-2.00)	1.17 (1.01-1.35)	1.39 (1.20-1.61)	0.91 (0.77-1.08)	0.96 (0.80-1.16)	0.57 (0.47-0.70)
		n	233	212	257	182	173	137	109	97
		N	1,573,648	2,037,124	1,534,770	1,694,130	1,337,120	1,636,230	1,193,081	1,760,851
	Female/Male	% (95% CI)	3.92	3.95	4.19	5.88	3.76	3.96	3.35	5.45
≥18	Male	% (95% CI)	0.07 (0.07-0.07)	0.13 (0.13-0.14)	0.06 (0.06-0.07)	0.12 (0.12-0.13)	0.05 (0.05-0.06)	0.12 (0.11-0.13)	0.05 (0.05-0.05)	0.10 (0.09-0.10)
		n	4,347	1,467	3,785	1,221	2,899	1,156	2,230	1,143

Age	Sex	Statistic	2017		2018		2019		2020	
			CCAE+MDCR	MDCD	CCAE+MDCR	MDCD	CCAE+MDCR	MDCD	CCAE+MDCR	MDCD
		N	6,296,580	1,097,149	5,960,702	993,438	5,336,863	950,638	4,683,825	1,175,736
	Female	% (95% CI)	53.76 (53.20-54.33)	83.77 (82.43-85.12)	51.36 (50.79-51.93)	88.63 (87.13-90.15)	48.47 (47.88-49.08)	81.06 (79.62-82.51)	41.99 (41.40-42.58)	67.24 (66.08-68.42)
		n	34,628	14,782	30,830	13,168	24,964	12,000	19,330	12,533
		N	6,905,775	2,011,533	6,444,112	1,726,140	5,623,884	1,713,883	4,949,589	2,006,685
	Female/Male	% (95% CI)	7.26	5.5	7.53	6.21	8.17	5.76	8.2	6.42
18-<40	Male	% (95% CI)	0.03 (0.03-0.04)	0.06 (0.05-0.07)	0.03 (0.03-0.03)	0.05 (0.05-0.06)	0.03 (0.02-0.03)	0.05 (0.05-0.06)	0.02 (0.02-0.03)	0.04 (0.03-0.04)
		n	806	408	725	331	553	308	473	277
		N	2,355,212	684,791	2,319,798	618,859	2,167,754	594,149	1,907,196	705,706
	Female	% (95% CI)	23.38 (22.76-24.02)	44.48 (43.30-45.69)	22.96 (22.34-23.60)	44.71 (43.41-46.04)	21.27 (20.63-21.92)	39.17 (37.95-40.42)	18.68 (18.05-19.32)	33.32 (32.30-34.36)
		n	5,281	5,317	5,090	4,433	4,160	3,855	3,298	4,018
		N	2,463,422	1,374,092	2,417,855	1,163,260	2,173,473	1,154,045	1,923,201	1,298,501
	Female/Male	% (95% CI)	6.26	6.49	6.74	7.12	7.5	6.44	6.91	7.88
40-<50	Male	% (95% CI)	0.08 (0.07-0.08)	0.24 (0.22-0.27)	0.07 (0.07-0.08)	0.24 (0.22-0.27)	0.06 (0.06-0.07)	0.22 (0.19-0.24)	0.05 (0.04-0.05)	0.17 (0.15-0.19)
		n	948	374	871	342	670	281	433	291
		N	1,202,404	155,431	1,172,013	140,318	1,068,158	130,683	931,348	169,236
	Female	% (95% CI)	58.47 (57.16-59.82)	153.65 (149.06-158.37)	60.04 (58.68-61.42)	161.15 (156.07-166.38)	55.65 (54.26-57.09)	144.40 (139.63-149.33)	48.82 (47.44-50.24)	115.24 (111.47-119.12)

Age	Sex	Statistic	2017		2018		2019		2020	
			CCAE+MDCR	MDCD	CCAE+MDCR	MDCD	CCAE+MDCR	MDCD	CCAE+MDCR	MDCD
		n	7,393	4,119	7,341	3,695	5,914	3,353	4,633	3,448
		N	1,337,999	301,781	1,294,525	264,018	1,143,418	265,831	1,004,769	319,133
	Female/Male	% (95% CI)	7.01	5.67	7.63	5.74	8.25	5.87	9.92	6.28
50-<60	Male	% (95% CI)	0.09 (0.09-0.10)	0.29 (0.26-0.31)	0.09 (0.08-0.09)	0.26 (0.23-0.29)	0.08 (0.07-0.08)	0.27 (0.25-0.30)	0.07 (0.07-0.08)	0.22 (0.19-0.24)
		n	1,270	480	1,218	384	929	377	769	374
		N	1,411,598	166,752	1,358,149	148,371	1,203,320	138,582	1,065,008	173,379
	Female	% (95% CI)	75.91 (74.54-77.32)	192.78 (186.87-198.89)	73.79 (72.39-75.21)	203.50 (196.98-210.23)	68.94 (67.49-70.43)	196.91 (190.44-203.60)	62.34 (60.87-63.84)	156.52 (151.38-161.83)
		n	11,387	3,877	10,442	3,549	8,358	3,376	6,743	3,390
		N	1,574,480	221,077	1,486,633	195,235	1,293,620	189,649	1,140,178	231,242
	Female/Male	% (95% CI)	8.04	6.09	7.83	7.02	8.37	6.54	8.19	6.8
60-<70	Male	% (95% CI)	0.10 (0.09-0.11)	0.24 (0.20-0.27)	0.09 (0.08-0.10)	0.20 (0.17-0.23)	0.08 (0.08-0.09)	0.22 (0.19-0.26)	0.07 (0.06-0.08)	0.17 (0.15-0.20)
		n	941	196	763	157	601	180	462	188
		N	939,115	82,726	862,038	79,875	734,406	80,858	657,657	110,998
	Female	% (95% CI)	78.55 (76.80-80.34)	160.27 (152.11-168.85)	73.84 (72.04-75.68)	179.56 (170.57-189.02)	70.23 (68.32-72.19)	165.09 (156.56-174.08)	58.59 (56.76-60.47)	134.70 (128.13-141.61)
		n	7,522	1,387	6,279	1,430	5,026	1,343	3,801	1,516
		N	1,048,617	97,859	935,571	91,788	801,043	92,177	717,314	125,128
	Female/Male	% (95% CI)	7.16	5.98	7.58	7.93	7.67	6.54	7.54	7.15

Age	Sex	Statistic	2017		2018		2019		2020	
			CCAE+MDCR	MDCD	CCAE+MDCR	MDCD	CCAE+MDCR	MDCD	CCAE+MDCR	MDCD
70-<80	Male	% (95% CI)	0.10 (0.09-0.12)	0.19 (0.09-0.36)	0.09 (0.08-0.11)	0.16 (0.06-0.33)	0.09 (0.08-0.11)	0.21 (0.10-0.39)	0.08 (0.06-0.10)	0.07 (0.03-0.15)
		n	250	9	145	7	99	10	63	8
		N	243,556	4,799	158,817	4,348	105,347	4,689	80,492	10,774
	Female	% (95% CI)	84.25 (80.82-87.82)	82.68 (65.47-104.40)	70.63 (66.83-74.65)	73.23 (56.41-95.06)	89.55 (84.48-94.92)	84.60 (66.65-107.38)	64.20 (59.41-69.38)	74.92 (63.25-88.75)
		n	2,208	70	1,247	56	1,123	67	634	133
		N	269,918	9,529	181,211	8,258	129,310	8,514	100,986	19,145
	Female/Male	% (95% CI)	7.97	3.92	7.54	4.21	9.24	3.69	8.02	9.36
≥80	Male	% (95% CI)	0.09 (0.08-0.11)	0.00 (0.00-0.14)	0.07 (0.05-0.09)	0.00 (0.00-0.22)	0.08 (0.06-0.11)	0.00 (0.00-0.22)	0.07 (0.05-0.10)	0.09 (0.03-0.21)
		n	132	0	63	0	47	0	30	5
		N	144,695	2,650	89,887	1,667	57,878	1,677	42,124	5,643
	Female	% (95% CI)	42.02 (39.28-44.96)	23.81 (13.53-41.90)	35.53 (32.33-39.04)	15.61 (6.50-37.48)	49.08 (44.42-54.24)	17.96 (8.07-39.94)	36.94 (32.39-42.14)	23.71 (16.38-34.33)
		n	837	12	431	5	383	6	221	28
		N	211,339	7,195	128,317	3,581	83,020	3,667	63,141	13,536
	Female/Male	% (95% CI)	4.34		4.79		5.68		4.91	2.33

CCAE=Commercial Claims and Encounters database; MDCR= Medicare Supplemental and Coordination of Benefits Database; MDCD=Medicaid Database

Supplementary Table 16: Annual incidence of diagnosed fibromyalgia (≥1 diagnosis code) by age groups and geographical regions in Commercial + Medicare databases

	2017			2018			2019			2020		
	CCAE+MDCR			CCAE+MDCR			CCAE+MDCR			CCAE+MDCR		
**Region**	**0-<18**	**≥18**	**All ages**	**0-<18**	**≥18**	**All ages**	**0-<18**	**≥18**	**All ages**	**0-<18**	**≥18**	**All ages**
Northeast Region	0.01 (0.01-0.01)	0.27 (0.26-0.27)	0.22 (0.22-0.23)	0.01 (0.01-0.02)	0.26 (0.26-0.27)	0.22 (0.21-0.22)	0.01 (0.01-0.01)	0.19 (0.18-0.20)	0.15 (0.15-0.16)	0.00 (0.00-0.00)	0.17 (0.17-0.18)	0.14 (0.14-0.15)
North Central Region	0.01 (0.01-0.01)	0.33 (0.33-0.34)	0.27 (0.26-0.28)	0.01 (0.01-0.01)	0.31 (0.30-0.31)	0.24 (0.24-0.25)	0.01 (0.01-0.01)	0.31 (0.30-0.31)	0.25 (0.24-0.25)	0.01 (0.00-0.01)	0.25 (0.25-0.26)	0.20 (0.20-0.21)
South Region	0.01 (0.01-0.01)	0.31 (0.30-0.31)	0.25 (0.25-0.25)	0.01 (0.01-0.01)	0.29 (0.28-0.29)	0.23 (0.23-0.24)	0.01 (0.01-0.01)	0.26 (0.26-0.27)	0.21 (0.21-0.22)	0.01 (0.00-0.01)	0.24 (0.23-0.24)	0.19 (0.19-0.19)
West Region	0.01 (0.01-0.01)	0.24 (0.23-0.25)	0.19 (0.18-0.19)	0.01 (0.01-0.01)	0.24 (0.24-0.25)	0.19 (0.19-0.20)	0.01 (0.01-0.01)	0.22 (0.21-0.22)	0.17 (0.17-0.18)	0.01 (0.01-0.01)	0.19 (0.18-0.19)	0.15 (0.14-0.15)
Unknown Region	0.00 (0.00-0.02)	0.24 (0.19-0.29)	0.16 (0.13-0.20)	0.01 (0.00-0.04)	0.24 (0.19-0.30)	0.17 (0.13-0.21)	0.01 (0.00-0.03)	0.22 (0.18-0.27)	0.16 (0.13-0.19)	0.02 (0.00-0.09)	0.20 (0.15-0.26)	0.16 (0.12-0.21)

CCAE=Commercial Claims and Encounters database; MDCR= Medicare Supplemental and Coordination of Benefits Database; MDCD=Medicaid Database

Supplementary Table 17: Annual incidence of diagnosed fibromyalgia by age groups and race/ethnicity in the Medicaid database

		MDCD		MDCD		MDCD		MDCD	
		**2017**		**2018**		**2019**		**2020**	
		**0-<18 years**	**≥18 years**	**0-<18 years**	**≥18 years**	**0-<18 years**	**≥18 years**	**0-<18 years**	**≥18 years**
Missing	% (95% CI)	0.01 (0.00-0.01)	0.58 (0.55-0.60)	0.01 (0.00-0.01)	0.62 (0.59-0.65)	0.01 (0.01-0.01)	0.66 (0.63-0.69)	0.00 (0.00-0.00)	0.52 (0.49-0.55)
	n	23	2,067	18	1,424	23	1,391	5	1,201
	N	404,541	357,628	231,482	229,753	239,955	210,946	260,916	232,043
White	% (95% CI)	0.01 (0.01-0.01)	0.71 (0.70-0.72)	0.01 (0.01-0.01)	0.73 (0.71-0.74)	0.01 (0.01-0.01)	0.67 (0.65-0.68)	0.01 (0.00-0.01)	0.59 (0.58-0.60)
	n	187	10,849	163	10,092	115	8,780	87	8,896
	N	1,923,407	1,525,714	1,681,032	1,390,684	1,558,037	1,314,467	1,571,982	1,513,948
Black	% (95% CI)	0.00 (0.00-0.00)	0.26 (0.25-0.27)	0.00 (0.00-0.00)	0.27 (0.26-0.28)	0.00 (0.00-0.00)	0.25 (0.24-0.26)	0.00 (0.00-0.00)	0.22 (0.21-0.23)
	n	43	2,744	24	2,514	29	2,347	12	2,158
	N	1,369,120	1,054,683	1,177,359	945,111	1,139,153	950,707	1,147,880	994,487
Hispanic	% (95% CI)	0.00 (0.00-0.01)	0.34 (0.30-0.38)	0.00 (0.00-0.00)	0.16 (0.13-0.19)	0.00 (0.00-0.00)	0.38 (0.34-0.42)	0.00 (0.00-0.00)	0.34 (0.32-0.37)
	n	13	327	2	118	5	396	8	819
	N	367,569	96,928	272,943	73,425	306,633	104,717	435,850	238,412
Others	% (95% CI)	0.00 (0.00-0.01)	0.36 (0.31-0.40)	0.01 (0.00-0.01)	0.30 (0.26-0.34)	0.00 (0.00-0.01)	0.29 (0.25-0.33)	0.00 (0.00-0.00)	0.30 (0.27-0.32)
	n	3	262	8	241	2	242	4	602
	N	137,328	73,729	138,768	80,605	141,708	83,684	223,501	203,531

MDCD=Medicaid Database

Supplementary Table 18: Disease severity among adults with fibromyalgia (≥2 diagnosis codes)

		CCAE+MDCR	MDCD	CCAE+MDCR	MDCD	CCAE+MDCR	MDCD
**Variable**		**12 months FU N=41,802**	**12 months FU N=23,758**	**24 months FU N=24,527**	**24 months FU N=15,965**	**36 months FU N=13,340**	**36 months FU N=10,134**
Severe fibromyalgia	n (%)	12,884 (30.8)	8,975 (37.8)	7,151 (29.2)	6,288 (39.4)	4,019 (30.1)	4,223 (41.7)
>Y* diagnostic codes/claims within X months of first diagnosis	n (%)	8,263 (19.8)	5,732 (24.1)	3,075 (12.5)	2,871 (18.0)	1,253 (9.4)	1,481 (14.6)
Incident events of disability in patients with fibromyalgia	n (%)	795 (1.9)	856 (3.6)	757 (3.1)	821 (5.1)	503 (3.8)	674 (6.7)
Incident events of hospitalization at pain clinics in patients with fibromyalgia	n (%)	76 (0.2)	3 (0.0)	3,079 (12.6)	5 (0.0)	40 (0.3)	5 (0.0)
Incident events of patients seen at pain clinics	n (%)	4,007 (9.6)	396 (1.7)	2,034 (8.3)	364 (2.3)	1,998 (15.0)	260 (2.6)
Number of concomitant medications (>=5) with pregabalin, duloxetine, milnacipran	n (%)	2,473 (5.9)	3,942 (16.6)		3,839 (24.0)	1,441 (10.8)	2,917 (28.8)

*Y=5 for X=12 months, Y=10 for X=24 months and Y=15 for X=36 months

CCAE=Commercial Claims and Encounters database; FU=Follow up; MDCR= Medicare Supplemental and Coordination of Benefits Database; MDCD=Medicaid Database

Supplementary Table 19: Disease severity among adolescents (12-<18 years) with fibromyalgia (≥2 diagnosis codes)

		CCAE+MDCR	MDCD	CCAE+MDCR	MDCD	CCAE+MDCR	MDCD
**Variable**		**12 months N=356**	**12 months N=276**	**24 months N=213**	**24 months N=209**	**36 months N=118**	**36 months N=133**
Severe fibromyalgia	n (%)	137 (38.5)	93 (33.7)	69 (32.4)	52 (24.9)	36 (30.5)	35 (26.3)
>Y* diagnostic codes/claims within X months of first diagnosis	n (%)	123 (34.6)	77 (27.9)	52 (24.4)	34 (16.3)	22 (18.6)	19 (14.3)
Incident events of disability in patients with fibromyalgia	n (%)	14 (3.9)	11 (4.0)	14 (6.6)	11 (5.3)	7 (5.9)	10 (7.5)
Incident events of hospitalization at pain clinics in patients with fibromyalgia	n (%)	0 (0.0)	0 (0.0)	0 (0.0)	0 (0.0)	0 (0.0)	0 (0.0)
Incident events of patients seen at pain clinics	n (%)	10 (2.8)	3 (1.1)	11 (5.2)	4 (1.9)	9 (7.6)	2 (1.5)
Number of concomitant medications (≥5) listed in suppl table 1 with pregabalin, duloxetine, milnacipran	n (%)	7 (2.0)	10 (3.6)	7 (3.3)	8 (3.8)	5 (4.2)	7 (5.3)

*Y=5 for X=12 months, Y=10 for X=24 months and Y=15 for X=36 months
CCAE=Commercial Claims and Encounters database; MDCR= Medicare Supplemental and Coordination of Benefits Database; MDCD=Medicaid Database

Supplementary Table 20: Disease severity among adults with fibromyalgia (≥1 diagnosis code)

		CCAE+MDCR	MDCD	CCAE+MDCR	MDCD	CCAE+MDCR	MDCD
**Variable**		**12 months FU N=41,802**	**12 months FU N=23,758**	**24 months FU N=24,527**	**24 months FU N=15,965**	**36 months FU N=13,340**	**36 months FU N=10,134**
Severe fibromyalgia	n (%)	16,013 (21.8)	11,020 (28.3)	9,647 (22.5)	8,247 (32.0)	5,668 (24.5)	5,761 (35.9)
>Y* diagnostic codes/claims within X months of first diagnosis	n (%)	8,263 (11.3)	5,732 (14.7)	3,132 (7.3)	2,916 (11.3)	1,300 (5.6)	1,536 (9.6)
Incident events of disability in patients with fibromyalgia	n (%)	1,173 (1.6)	1,313 (3.4)	1,119 (2.6)	1,244 (4.8)	755 (3.3)	1,036 (6.5)
Incident events of hospitalization at pain clinics in patients with fibromyalgia	n (%)	115 (0.2)	3 (0.0)	93 (0.2)	7 (0.0)	51 (0.2)	7 (0.0)
Incident events of patients seen at pain clinics	n (%)	6,154 (8.4)	605 (1.6)	4,712 (11.0)	552 (2.1)	3,066 (13.2)	402 (2.5)
Number of concomitant medications (≥5) listed in suppl table 1 with pregabalin, duloxetine, milnacipran	n (%)	3,289 (4.5)	5,442 (14.0)	2,771 (6.5)	5,301 (20.6)	1,977 (8.5)	4,069 (25.3)

*Y=5 for X=12 months, Y=10 for X=24 months and Y=15 for X=36 months

CCAE=Commercial Claims and Encounters database; FU=Follow up; MDCR= Medicare Supplemental and Coordination of Benefits Database; MDCD=Medicaid Database

Supplementary Table 21: Disease severity among adolescents (12-<18 years) with fibromyalgia (≥1 diagnosis code)

		CCAE+MDCR	MDCD	CCAE+MDCR	MDCD	CCAE+MDCR	MDCD
**Variable**		**12 months N=356**	**12 months N=276**	**24 months N=213**	**24 months N=209**	**36 months N=118**	**36 months N=133**
Severe fibromyalgia	n (%)	154 (25.9)	100 (19.5)	85 (23.9)	62 (15.8)	43 (22.4)	43 (18.1)
>Y* diagnostic codes/claims within X months of first diagnosis	n (%)	123 (20.7)	77 (15.0)	54 (15.2)	34 (8.7)	22 (11.5)	19 (8.0)
Incident events of disability in patients with fibromyalgia	n (%)	19 (3.2)	14 (2.7)	20 (5.6)	15 (3.8)	9 (4.7)	14 (5.9)
Incident events of hospitalization at pain clinics in patients with fibromyalgia	n (%)	0 (0.0)	0 (0.0)	0 (0.0)	1 (0.3)	0 (0.0)	0 (0.0)
Incident events of patients seen at pain clinics	n (%)	21 (3.5)	5 (1.0)	18 (5.1)	7 (1.8)	12 (6.3)	4 (1.7)
Number of concomitant medications (≥5) listed in suppl table 1 with pregabalin, duloxetine, milnacipran	n (%)	9 (1.5)	12 (2.3)	11 (3.1)	12 (3.1)	7 (3.6)	9 (3.8)

*Y=5 for X=12 months, Y=10 for X=24 months and Y=15 for X=36 months

CCAE=Commercial Claims and Encounters database; MDCR= Medicare Supplemental and Coordination of Benefits Database; MDCD=Medicaid Database

Supplementary Table 22: Comorbidities among adults with fibromyalgia (≥2 diagnosis codes)

	CCAE+MDCR	MDCD	CCAE+MDCR	MDCD	CCAE+MDCR	MDCD	CCAE+MDCR	MDCD
**Variable**	**12 months BL N=41,802, n (%)**	**12 months BL N=23,758, n (%)**	**12 months FU N=41,802, n (%)**	**12 months FU N=23,758, n (%)**	**24 months FU N=24,527, n (%)**	**24 months FU N=15,965, n (%)**	**36 months FU N=13,340, n (%)**	**36 months FU N=10,134, n (%)**
Other and unspecific soft tissue disorders, not elsewhere classified	18,232 (43.6)	12,076 (50.8)	41,802 (100)	23,758 (100)	24,527 (100)	15,965 (100)	13,340 (100)	10,134 (100)
Dorsalgia*	22,347 (53.5)	15,091 (63.5)	24,420 (58.4)	16,493 (69.4)	17,073 (69.6)	12,841 (80.4)	10,200 (76.5)	8,689 (85.7)
Other joint disorder, not elsewhere classified	21,050 (50.4)	13,039 (54.9)	22,763 (54.5)	14,057 (59.2)	16,445 (67.0)	11,656 (73.0)	9,847 (73.8)	8,092 (79.9)
Encounter for screening for malignant neoplasms	18,058 (43.2)	7,118 (30.0)	18,811 (45.0)	7,613 (32.0)	15,538 (63.4)	7,684 (48.1)	9,650 (72.3)	5,859 (57.8)
Abdominal and pelvic pain*	12,702 (30.4)	9,901 (41.7)	13,209 (31.6)	10,592 (44.6)	11,178 (45.6)	9,422 (59.0)	7,335 (55.0)	6,847 (67.6)
Other anxiety disorders*	13,231 (31.7)	12,079 (50.8)	16,009 (38.3)	14,051 (59.1)	11,628 (47.4)	11,155 (69.9)	7,064 (53.0)	7,678 (75.8)
Malaise and fatigue*	13,844 (33.1)	7,422 (31.2)	15,419 (36.9)	8,525 (35.9)	12,033 (49.1)	7,805 (48.9)	7,679 (57.6)	5,777 (57.0)
Essential (primary) hypertension	16,016 (38.3)	10,692 (45.0)	17,910 (42.8)	11,828 (49.8)	12,027 (49.0)	9,171 (57.4)	6,961 (52.2)	6,258 (61.8)

	**CCAE+MDCR**	**MDCD**	**CCAE+MDCR**	**MDCD**	**CCAE+MDCR**	**MDCD**	**CCAE+MDCR**	**MDCD**
**Variable**	**12 months BL N=41,802, n (%)**	**12 months BL N=23,758, n (%)**	**12 months FU N=41,802, n (%)**	**12 months FU N=23,758, n (%)**	**24 months FU N=24,527, n (%)**	**24 months FU N=15,965, n (%)**	**36 months FU N=13,340, n (%)**	**36 months FU N=10,134, n (%)**
Pain, not elsewhere classified*	10,408 (24.9)	10,493 (44.2)	13,486 (32.3)	12,802 (53.9)	10,241 (41.8)	10,455 (65.5)	6,354 (47.6)	7,319 (72.2)
Disorders of lipoprotein metabolism and other lipidemias	15,094 (36.1)	7,701 (32.4)	16,778 (40.1)	8,980 (37.8)	12,154 (49.6)	7,659 (48.0)	7,291 (54.7)	5,483 (54.1)
Gastro-esophageal reflux disease	10,401 (24.9)	8,138 (34.3)	12,667 (30.3)	9,886 (41.6)	10,019 (40.8)	8,572 (53.7)	6,318 (47.4)	6,130 (60.5)
Pain in throat and chest	8,831 (21.1)	7,737 (32.6)	9,672 (23.1)	8,539 (35.9)	8,518 (34.7)	8,064 (50.5)	5,722 (42.9)	5,995 (59.2)
Sleep disorders*	10,622 (25.4)	6,342 (26.7)	13,299 (31.8)	7,930 (33.4)	9,897 (40.4)	6,869 (43.0)	6,233 (46.7)	5,021 (49.5)
Abnormalities of breathing	8,031 (19.2)	7,034 (29.6)	9,487 (22.7)	7,993 (33.6)	8,162 (33.3)	7,563 (47.4)	5,480 (41.1)	5,719 (56.4)
Cough	8,060 (19.3)	6,074 (25.6)	8,485 (20.3)	6,467 (27.2)	8,101 (33.0)	6,684 (41.9)	5,652 (42.4)	5,178 (51.1)
Major depressive disorder, single episode*	7,945 (19.0)	8,191 (34.5)	10,213 (24.4)	9,807 (41.3)	7,913 (32.3)	8,506 (53.3)	5,015 (37.6)	6,107 (60.3)
Overweight and obesity	9,250 (22.1)	7,261 (30.6)	11,322 (27.1)	8,715 (36.7)	9,042 (36.9)	7,525 (47.1)	5,751 (43.1)	5,317 (52.5)
Vitamin D deficiency	9,800 (23.4)	5,045 (21.2)	11,963 (28.6)	6,498 (27.4)	9,442 (38.5)	6,016 (37.7)	6,030 (45.2)	4,495 (44.4)

	CCAE+MDCR	MDCD	CCAE+MDCR	MDCD	CCAE+MDCR	MDCD	CCAE+MDCR	MDCD
**Variable**	**12 months BL N=41,802, n (%)**	**12 months BL N=23,758, n (%)**	**12 months FU N=41,802, n (%)**	**12 months FU N=23,758, n (%)**	**24 months FU N=24,527, n (%)**	**24 months FU N=15,965, n (%)**	**36 months FU N=13,340, n (%)**	**36 months FU N=10,134, n (%)**
Spondylosis	8,207 (19.6)	6,084 (25.6)	9,785 (23.4)	7,350 (30.9)	7,743 (31.6)	6,611 (41.4)	4,983 (37.4)	4,936 (48.7)
Headache*	6,950 (16.6)	5,875 (24.7)	7,583 (18.1)	6,402 (26.9)	6,693 (27.3)	6,279 (39.3)	4,575 (34.3)	4,871 (48.1)
Rheumatoid arthritis	4,364 (10.4)	1,737 (7.3)	5,767 (13.8)	2,503 (10.5)	3,949 (16.1)	2,069 (13.0)	2,365 (17.7)	1,469 (14.5)
Systemic lupus erythematosus	1,591 (3.8)	869 (3.7)	2,011 (4.8)	1,136 (4.8)	1,344 (5.5)	907 (5.7)	843 (6.3)	637 (6.3)
Ankylosing spondylitis	350 (0.8)	113 (0.5)	535 (1.3)	166 (0.7)	409 (1.7)	155 (1.0)	250 (1.9)	125 (1.2)
Psoriatic arthritis	640 (1.5)	200 (0.8)	890 (2.1)	290 (1.2)	628 (2.6)	250 (1.6)	394 (3.0)	170 (1.7)
Charlson co-morbidity index (CCI) categories								
Mild (1-2)	16,405 (39.2)	10,612 (44.7)	17,782 (42.5)	11,017 (46.4)	11,170 (45.5)	7,301 (45.7)	6,182 (46.3)	4,448 (43.9)
Moderate (3-4)	3,283 (7.9)	2,970 (12.5)	3,995 (9.6)	3,658 (15.4)	3,163 (12.9)	3,146 (19.7)	2,084 (15.6)	2,243 (22.1)
Severe (5)	469 (1.1)	568 (2.4)	680 (1.6)	697 (2.9)	626 (2.6)	739 (4.6)	412 (3.1)	552 (5.4)
Very severe (>=6)	722 (1.7)	830 (3.5)	1,087 (2.6)	1,185 (5.0)	962 (3.9)	1,297 (8.1)	633 (4.7)	1,151 (11.4)

BL=Baseline; CCAE=Commercial Claims and Encounters database; CCI= Charlson co-morbidity index; FU=Follow up; MDCR= Medicare Supplemental and Coordination of Benefits Database; MDCD=Medicaid Database

Top 20 comorbidities along with predefined rheumatic comorbidities (rheumatic arthritis, systemic lupus erythematosus, ankylosing spondylitis and psoriatic arthritis) among individuals with incident fibromyalgia were described

*=These will be considered as symptoms of fibromyalgia rather than comorbidities

CCI categories: Mild (1-2), moderate (3-4), severe (5), very severe (≥6)

Charlson comorbidity with assigned weights included acute myocardial infarction (1), congestive heart failure (1), peripheral vascular disease (1), cerebrovascular disease (1), dementia (1), chronic pulmonary disease (1), rheumatoid disease (1), peptic ulcer disease (1), mild (1) and moderate/severe liver disease (3), diabetes mellitus with (2) and without complications (1), hemiplegia/paraplegia (2), renal disease (2), any malignancy, including leukemia and lymphoma (2) and metastatic solid tumors (6), Acquired immunodeficiency syndrome/Human immunodeficiency virus (AIDS/HIV) (6).

Supplementary Table 23: Comorbidities among adults with fibromyalgia (≥1 diagnosis code)

	CCAE+MDCR	MDCD	CCAE+MDCR	MDCD	CCAE+MDCR	MDCD	CCAE+MDCR	MDCD
**Variable**	**12 months BL N=73,323**	**12 months BL N=38,920**	**12 months FU N=73,323**	**12 months FU N=38,920**	**24 months FU N=42,832**	**24 months FU N=25,773**	**36 months FU N=23,147**	**36 months FU N=16,054**
Other and unspecific soft tissue disorders, not elsewhere classified	29,807 (40.7)	18,868 (48.5)	73,323 (100)	38,920 (100)	42,832 (100)	25,773 (100)	23,147 (100)	16,054 (100)
Dorsalgia*	37,199 (50.7)	24,033 (61.7)	39,874 (54.4)	25,515 (65.6)	28,245 (65.9)	19,999 (77.6)	16,875 (72.9)	13,438 (83.7)
Other joint disorder, not elsewhere classified	34,734 (47.4)	20,425 (52.5)	36,552 (49.9)	21,379 (54.9)	26,940 (62.9)	17,912 (69.5)	16,446 (71.1)	12,371 (77.1)
Encounter for screening for malignant neoplasms	31,521 (43.0)	11,387 (29.3)	32,718 (44.6)	11,946 (30.7)	26,836 (62.7)	12,031 (46.7)	16,629 (71.8)	9,036 (56.3)
Abdominal and pelvic pain*	21,163 (28.9)	15,914 (40.9)	21,828 (29.8)	16,446 (42.3)	18,533 (43.3)	14,660 (56.9)	12,154 (52.5)	10,535 (65.6)
Other anxiety disorders*	21,712 (29.6)	19,285 (49.6)	25,876 (35.3)	21,940 (56.4)	19,029 (44.4)	17,427 (67.6)	11,622 (50.2)	11,806 (73.5)
Malaise and fatigue*	22,641 (30.9)	11,629 (29.9)	24,712 (33.7)	12,871 (33.1)	19,667 (45.9)	11,917 (46.2)	12,609 (54.5)	8,801 (54.8)
Essential (primary) hypertension	28,059 (38.3)	17,872 (45.9)	31,135 (42.5)	19,476 (50.0)	20,874 (48.7)	14,892 (57.8)	11,957 (51.7)	10,003 (62.3)
Pain, not elsewhere classified*	16,672 (22.7)	16,648 (42.8)	21,033 (28.7)	19,554 (50.2)	16,202 (37.8)	16,001 (62.1)	10,106 (43.7)	11,116 (69.2)

	CCAE+MDCR	MDCD	CCAE+MDCR	MDCD	CCAE+MDCR	MDCD	CCAE+MDCR	MDCD
**Variable**	**12 months BL N=73,323**	**12 months BL N=38,920**	**12 months FU N=73,323**	**12 months FU N=38,920**	**24 months FU N=42,832**	**24 months FU N=25,773**	**36 months FU N=23,147**	**36 months FU N=16,054**
Disorders of lipoprotein metabolism and other lipidemias	26,801 (36.6)	12,857 (33.0)	29,567 (40.3)	14,578 (37.5)	21,235 (49.6)	12,349 (47.9)	12,614 (54.5)	8,650 (53.9)
Gastro-esophageal reflux disease	17,648 (24.1)	13,102 (33.7)	21,062 (28.7)	15,396 (39.6)	16,792 (39.2)	13,312 (51.7)	10,617 (45.9)	9,412 (58.6)
Pain in throat and chest	14,871 (20.3)	12,448 (32.0)	15,969 (21.8)	13,283 (34.1)	14,145 (33.0)	12,551 (48.7)	9,485 (41.0)	9,313 (58.0)
Sleep disorders*	17,487 (23.8)	10,167 (26.1)	21,411 (29.2)	12,127 (31.2)	16,053 (37.5)	10,475 (40.6)	10,101 (43.6)	7,565 (47.1)
Abnormalities of breathing	13,393 (18.3)	11,332 (29.1)	15,360 (20.9)	12,449 (32.0)	13,315 (31.1)	11,828 (45.9)	8,936 (38.6)	8,832 (55.0)
Cough	13,472 (18.4)	9,671 (24.8)	14,384 (19.6)	10,141 (26.1)	13,535 (31.6)	10,441 (40.5)	9,516 (41.1)	8,009 (49.9)
Major depressive disorder, single episode*	12,944 (17.7)	12,944 (33.3)	16,038 (21.9)	14,947 (38.4)	12,656 (29.5)	13,030 (50.6)	8,047 (34.8)	9,295 (57.9)
Overweight and obesity	15,464 (21.1)	11,576 (29.7)	18,590 (25.4)	13,443 (34.5)	14,907 (34.8)	11,587 (45.0)	9,447 (40.8)	8,183 (51.0)
Vitamin D deficiency	16,553 (22.6)	7,848 (20.2)	19,521 (26.6)	9,716 (25.0)	15,615 (36.5)	9,104 (35.3)	9,933 (42.9)	6,770 (42.2)
Spondylosis	13,504 (18.4)	9,655 (24.8)	15,492 (21.1)	11,036 (28.4)	12,303 (28.7)	9,895 (38.4)	7,935 (34.3)	7,351 (45.8)
Headache*	11,319 (15.4)	9,427 (24.2)	12,192 (16.6)	9,795 (25.2)	10,790 (25.2)	9,611 (37.3)	7,428 (32.1)	7,390 (46.0)
Rheumatoid arthritis	6,860 (9.4)	2,613 (6.7)	8,710 (11.9)	3,588 (9.2)	5,959 (13.9)	2,968 (11.5)	3,547 (15.3)	2,120 (13.2)
Systemic lupus erythematosus	2,426 (3.3)	1,295 (3.3)	3,036 (4.1)	1,641 (4.2)	2,062 (4.8)	1,301 (5.0)	1,276 (5.5)	907 (5.6)

	CCAE+MDCR	MDCD	CCAE+MDCR	MDCD	CCAE+MDCR	MDCD	CCAE+MDCR	MDCD
**Variable**	**12 months BL N=73,323**	**12 months BL N=38,920**	**12 months FU N=73,323**	**12 months FU N=38,920**	**24 months FU N=42,832**	**24 months FU N=25,773**	**36 months FU N=23,147**	**36 months FU N=16,054**
Ankylosing spondylitis	567 (0.8)	178 (0.5)	798 (1.1)	256 (0.7)	597 (1.4)	230 (0.9)	381 (1.6)	170 (1.1)
Psoriatic arthritis	1,033 (1.4)	312 (0.8)	1,380 (1.9)	429 (1.1)	976 (2.3)	356 (1.4)	590 (2.5)	246 (1.5)
Charlson comorbidity index categories								
Mild (1-2)	27,953 (38.1)	16,996 (43.7)	29,953 (40.9)	17,709 (45.5)	18,896 (44.1)	11,648 (45.2)	10,522 (45.5)	6,984 (43.5)
Moderate (3-4)	5,577 (7.6)	4,992 (12.8)	6,634 (9.0)	5,817 (14.9)	5,245 (12.2)	4,940 (19.2)	3,409 (14.7)	3,485 (21.7)
Severe (5)	795 (1.1)	928 (2.4)	1,089 (1.5)	1,115 (2.9)	977 (2.3)	1,154 (4.5)	625 (2.7)	872 (5.4)
Very severe (>=6)	1,236 (1.7)	1,460 (3.8)	1,765 (2.4)	1,959 (5.0)	1,581 (3.7)	2,126 (8.2)	1,037 (4.5)	1,821 (11.3)

BL=Baseline; CCAE=Commercial Claims and Encounters database; FU=Follow up; MDCR= Medicare Supplemental and Coordination of Benefits Database; MDCD=Medicaid Database

Top 20 comorbidities along with predefined rheumatic comorbidities (rheumatic arthritis, systemic lupus erythematosus, ankylosing spondylitis and psoriatic arthritis) among individuals with incident fibromyalgia were described

*=These will be considered as symptoms of fibromyalgia rather than comorbidities

CCI categories: Mild (1-2), moderate (3-4), severe (5), very severe (≥6)

Charlson comorbidity with assigned weights included acute myocardial infarction (1), congestive heart failure (1), peripheral vascular disease (1), cerebrovascular disease (1), dementia (1), chronic pulmonary disease (1), rheumatoid disease (1), peptic ulcer disease (1), mild (1) and moderate/severe liver disease (3), diabetes mellitus with (2) and without complications (1), hemiplegia/paraplegia (2), renal disease (2), any malignancy, including leukemia and lymphoma (2) and metastatic solid tumors (6), Acquired immunodeficiency syndrome/Human immunodeficiency virus (AIDS/HIV) (6).
